# Comparative microbiome diversity in root-nodules of three *Desmodium* species used in push-pull cropping system

**DOI:** 10.3389/fmicb.2024.1395811

**Published:** 2024-06-20

**Authors:** Isack H. Adan, George Ochieng Asudi, Saliou Niassy, Abdul A. Jalloh, Johnstone Mutiso Mutua, Frank Chidawanyika, Fathiya Khamis, Zeyaur Khan, Sevgan Subramanian, Thomas Dubois, Daniel Munyao Mutyambai

**Affiliations:** ^1^International Centre of Insect Physiology and Ecology, Nairobi, Kenya; ^2^Department of Biochemistry, Microbiology, and Biotechnology, Kenyatta University, Nairobi, Kenya; ^3^Inter-African Phytosanitary Council of the African Union, Yaoundé, Cameroon; ^4^Department of Zoology and Entomology, University of Pretoria, Pretoria, South Africa; ^5^Department of Zoology and Entomology, University of Free State, Bloemfontein, South Africa; ^6^Department of Life Science, South Eastern Kenya University, Kitui, Kenya

**Keywords:** 16S and ITS, Amplicon sequencing, *Desmodium* species, root-nodules microbiome, push-pull cropping system

## Abstract

**Background:**

*Desmodium* species used as intercrops in push-pull cropping systems are known to repel insect-pests, suppress *Striga* species weeds, and shift soil microbiome. However, the mechanisms through which *Desmodium* species impact the soil microbiome, either through its root exudates, changes in soil nutrition, or shading microbes from its nodules into the rhizosphere, are less understood. Here, we investigated the diversity of root-nodule microbial communities of three *Desmodium* species- *Desmodium uncinatum* (SLD), *Desmodium intortum* (GLD), and *Desmodium incanum* (AID) which are currently used in smallholder maize push-pull technology (PPT).

**Methods:**

*Desmodium* species root-nodule samples were collected from selected smallholder farms in western Kenya, and genomic DNA was extracted from the root-nodules. The amplicons underwent paired-end Illumina sequencing to assess bacterial and fungal populations.

**Results:**

We found no significant differences in composition and relative abundance of bacterial and fungal species within the root-nodules of the three *Desmodium* species. While a more pronounced shift was observed for fungal community compositions compared to bacteria, no significant differences were observed in the general diversity (evenness and richness) of fungal and bacterial populations among the three *Desmodium* species. Similarly, beta diversity was not significantly different among the three *Desmodium* species. The root-nodule microbiome of the three *Desmodium* species was dominated by *Bradyrhizobium* and *Fusarium* species. Nevertheless, there were significant differences in the proportion of marker gene sequences responsible for energy and amino acid biosynthesis among the three *Desmodium* species, with higher sequence proportions observed in SLD.

**Conclusion:**

There is no significant difference in the microbial community of the three *Desmodium* species used in PPT. However, root-nodule microbiome of SLD had significantly higher marker gene sequences responsible for energy and amino acid biosynthesis. Therefore, it is likely that the root-nodules of the three *Desmodium* species host similar microbiomes and influence soil health, consequently impacting plant growth and agroecosystem functioning.

## 1 Introduction

Africa grapples with the escalating challenge of feeding its rapidly growing population, leading to high rates of hunger and poverty (OECD/FAO, 2016). Addressing this crisis necessitates enhancing agricultural productivity to curb hunger and poverty and ensure food security (Tadele, 2017). One promising approach involves synergizing perennial and feed legumes in agroecological systems, such as push-pull technology (PPT), offering an alternative for elevating agricultural sustainability (Khan et al., 2014; Tadele, 2017). Since its inception in the mid-1990s, PPT has been recognized as a pathway for sustainable cereal crop intensification against lepidopteran pests, parasitic *Striga* weeds, and biodiversity restoration (Khan et al., 2014; Mutyambai et al., 2023). The strategy provides additional ecosystem services such as soil phytoremediation, moisture content regulation, and shifts in soil microbial and physico-chemical properties (Drinkwater et al., 2021; Jalloh et al., 2024). PPT involves intercropping perennial *Desmodium* species alongside maize while bordering the plantings with Napier or *Brachiaria* grass (Khan et al., 2014). The ‘pull' plant (*Brachiaria*/Napier grass) produces a gummy substance in reaction to the penetration of the stemborer larvae, which causes the death of early instar larvae (Khan and Pickett, 2004). On the other hand, the “push” plant (*Desmodium* species) emits repellent volatiles against crop pests, limiting oviposition preference of female moths (Cheruiyot et al., 2018; Mutyambai et al., 2019; Peter et al., 2023). *Desmodium* species also prevent *Striga hermonthica* parasitism by inducing allelopathic suicidal germination, thereby providing a novel means of *in situ* reductions of the *Striga* seed bank in soil (Tsanuo et al., 2003). Despite the existence of several *Desmodium* species worldwide, only a few species have been utilized in PPT.

Globally, 350 *Desmodium* species have been delineated, of which only 39 species have been identified in Africa (Ma et al., 2011). Of these *Desmodium* species, PPT has utilized three, including silverleaf desmodium (SLD) [*Desmodium uncinatum* (Jacq.) DC] in the conventional PPT (Khan et al., 2000), greenleaf desmodium (GLD) [*D. intortum*; (Mill.) Urb.] in the climate-smart PPT (Khan et al., 2016a), and African desmodium (AID) (*D. incanum* DC.) in the third-generation PPT (Cheruiyot et al., 2021). In addition, *Desmodium* species have specialized interactions with host-specific endophytic microbes such as rhizobia, forming atmospheric N_2_-fixing root-nodules that have been reported to yield up to 90 kg N ha^−1^ seasonally (Gu et al., 2007; Ojiem et al., 2007; Xu et al., 2016). Moreover, *Desmodium* plants enhance the availability of major nutrients such as phosphorus and carbon in agricultural soils (Drinkwater et al., 2021; Ndayisaba et al., 2022, 2023). AID also effectively remediates petroleum-degraded soils, favoring the multiplication of rhizospheric microbiota (Kitamura and Maranho, 2016). However, the mechanisms through which *Desmodium* plants affect the below-ground microbial communities have been largely unknown.

Beneath the surface, plant roots engage with a diverse soil microbiome, shaping a localized plant-soil feedback mechanism (Mutyambai et al., 2019; Hannula et al., 2021). This interaction, guided by microbial communities, positively influences soil nutrient cycling, fostering beneficial microbe s survival and plant growth and defense against insects (Muthini et al., 2020; Hannula et al., 2021; Mutyambai et al., 2023; Jalloh et al., 2024). The plant root microbiome is shaped by microbe historical contingency (Carlström et al., 2019), interspecies microbial competition, rhizodeposit signaling cues (flavonoids), and numerous symbiosis (*Nod*) genes (Curtis et al., 2002; Shi et al., 2011; Oldroyd, 2013). Plants cannot assimilate nitrogen, the primary limiting nutrient in agriculture, due to their inability to break down existing complex bonds (Oldroyd, 2013). However, rhizobia colonizes legume plant roots, forming an endosymbiotic relationship that ensures nitrogen is converted to ammonia via biological nitrogen fixation (BNF) (Jalloh et al., 2020; Wekesa et al., 2022). This happens in specialized organs known as root-nodules, which are formed via an intricate association with rhizobia (Oldroyd, 2013). While nodulation has long been studied as a two-member system (legume plant and rhizobia), recent research highlights complex and distinct microbes residing in separate ecological microniches (Hartman et al., 2017).

Rhizobia, the nitrogen-fixing bacteria crucial for nodulation, operate in harmony with other representative microbes, forming a balanced association with non-rhizobial endophytes (NREs) like *Azospirillum, Devosia, Bacillus, Pseudomonas*, and *Streptomyces* (Mayhood and Mirza, 2021). While information on nodule-colonizing fungi remains limited, various genera, such as *Aspergillus, Glomus, Penicillium, Trichoderma, Macrophomina, Fusarium*, and *Rhizoctonia*, have been isolated from legume root-nodules using culture techniques (Muthini et al., 2020; Mayhood and Mirza, 2021). These bacterial and fungal endophytes facilitate nutrient cycling and acquisition, siderophore production, antibiotic resistance, phosphate solubilization, bio-defense against pathogens, regulation of osmotic pressure, hydrolytic enzyme production and secretion of metabolites (Rajendran et al., 2008; Dakora, 2015; Hartman et al., 2017; Drinkwater et al., 2021; Jalloh et al., 2024). These NREs have also been proven to positively impact plant growth when co-inoculated with rhizobia (Mayhood and Mirza, 2021). Consequently, this legume-microbe association holds significant environmental benefits by reducing the overreliance on/and disproportionate use of synthetic chemical fertilizers, which are known contributors to human and environmental hazards (Lupini et al., 2023).

The existing body of research has extensively examined and documented plant-plant and plant-insect interactions within PPT (Khan et al., 2016b; Mutyambai et al., 2016, 2019, 2023). However, the domain of below-ground interactions, particularly focusing on microbial communities inhabiting *Desmodium* root-nodules and their potential ecological benefits, remains largely unexplored. In this study, we hypothesized that *Desmodium* species root-nodules serve as habitats for diverse fungal and bacterial communities. Exploring these belowground interactions is anticipated to uncover novel dimensions that could significantly contribute to enhancing the sustainability and productivity of PPT.

## 2 Materials and methods

### 2.1 Sampling site

**Figure 1:**
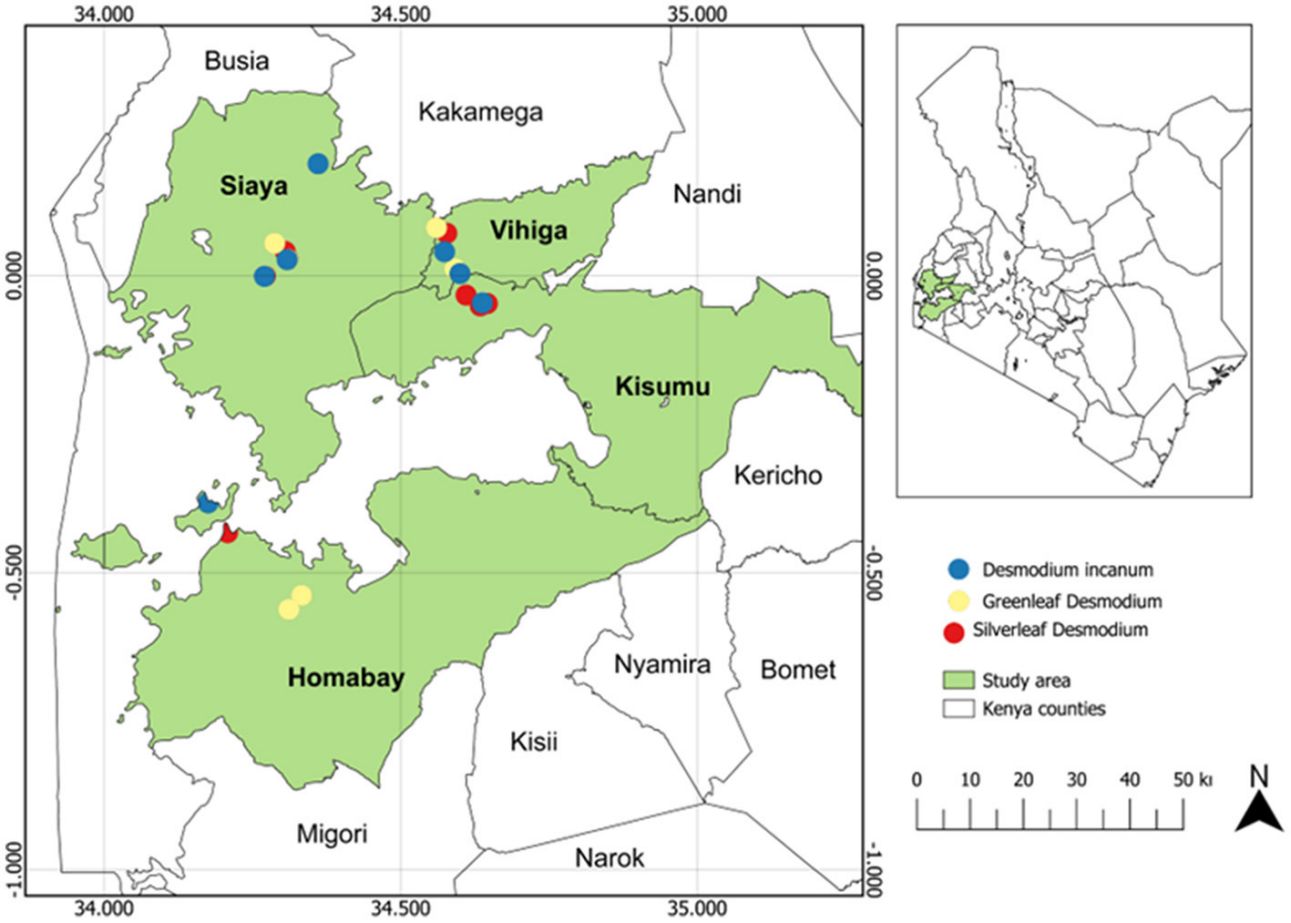
Map of Kenya showing maize push-pull farms where *Desmodium* root-nodules samples were collected.

The study was carried out in western Kenya across four counties: Homabay, Siaya, Vihiga and Kisumu (Figure 1; Supplementary Table S1). Root-nodule samples of *Desmodium* species were collected from 24 smallholder maize PPT farmers' fields, with eight PPT farms for each *Desmodium* species that were already established. Western Kenya soils are predominantly ferralsol, cambisols, nitisols, and acrisols and have varying physicochemical properties based on agronomic practices (Mutyambai et al., 2019; Ndayisaba et al., 2023). Climate conditions in the study areas vary, with a mean annual temperature of 25 ± 2°C and a bimodal annual rainfall averaging 1,395 – 1,500 mm per annum (p.a). Vihiga receives 1,800 – 2,000 mm of rainfall p.a, an average of 24°C, and altitudes ranging from 1,300 to 1,500 m above sea level (masl). Siaya experiences an average rainfall of 800 – 2,000 mm p.a, with a mean annual temperature of 21°C, and altitudes ranging from 1,140–1,400 masl (Odendo et al., 2010). Homabay receives an average rainfall of 1,000–1,250 mm pa, with a mean annual temperature of 15–30°C, with an average altitude of 1,146 masl (Ogenga, 2021). Kisumu records an average of 1,362 mm p.a., temperatures ranging from 18–34°C, and altitudes ranging from 1,100 to 1,131 masl (Nyberg et al., 2020). Soil temperature and relative humidity were obtained from Open-Meteo (https://open-meteo.com/) using their Python Application Programming Interface (API) for the year 2022 (Table 1; Supplementary Table S1).

**Table 1:** Comparative analysis of weather data for relative humidity and soil temperature across the study counties.

**Variable**	**Soil depth (cm)**	**Studied counties**				***F*-value _(2, 21)_**	***P*-value**
		**Vihiga**	**Siaya**	**Homabay**	**Kisumu**		
Soil temperature	7-28	21.7 ± 0.512^a^	22.1 ± 0.309^a^	22.8 ± 0.512^a^	22.1 ± 0.458^a^	0.7785	0.5198
Relative humidity	-	76.0 ± 0.436 ^bc^	77.3 ± 0.263 ^c^	72.8 ± 0.436^a^	75.2 ± 0.390^b^	28	0.001

Mean ± SE. Different letters across the rows indicate a significant difference (P < 0.05) according to Tukeys honest significance (HSD) test.

All sampled smallholder farmers' fields practiced PPT with *Brachiaria* (*Brachiaria* cv mulato II) as the border crop. The age of PPT farms ranged from 5 to 20 years, relying on seasonal rainfall without any synthetic chemical inputs throughout the cultivation period (Mutyambai et al., 2019; Jalloh et al., 2024).

### 2.2 Sample collection

*Desmodium* species root-nodule samples were collected during the short rain season from November to December 2022 when *Desmodium* plants were at the flowering stage (Supplementary Table S1). A 70% ethanol solution was used to sterilize the spade, which was used to detach the rooting system from five healthy *Desmodium* plants selected from five separate rows in each smallholder maize PPT field. Afterwards, 10 intact root-nodules were detached from each of the five plants after shaking the *Desmodium* plant roots to remove adhering rhizospheric soil, followed by careful washing under running water. These root-nodules were subjected to drying using sterile adsorbent paper, and finally placed in closed-cap 10 mL sterile centrifuge tubes (Thermo Fischer Scientific, Wilmington, USA). The collected root-nodule samples were transported in a cooler box to the International Center of Insect Physiology and Ecology (*icipe*), Nairobi, Kenya, and stored at 4°C before further analysis.

### 2.3 Root-nodules genomic DNA extraction, library preparation and sequencing

Genomic DNA (gDNA) extraction was carried out using a modified protocol of Fukuda et al. (2022). Four healthy nodules were selected from each *Desmodium* plant based on consistency in size and shape making a total of 20 *Desmodium* root-nodules per farm for each *Desmodium* species which were pooled together during analysis. After that, they were sterilized by washing with 70% ethanol and 1% sodium hypochlorite for 2 min each to remove air bubbles from the tissues and reduce surface tension. This was followed by six-time washes with sterile distilled water, as previously described by Da Silva et al. (2021). To validate sterility, the distilled water from the last rinsing step was plated on yeast extract mannitol agar (YEMA) and potato dextrose agar (PDA) (Oxoid Ltd, Basingstoke Hampshire RG24, UK), and incubated at 28 ± 2°C for 48 h and 25 ± 2°C for 72 h for bacteria and fungi, respectively (Vincent, 1970; Jing et al., 2022; Jalloh et al., 2024). The nodules were air-dried on sterile blotting paper in a sterilized laminar flow cabinet and aseptically crushed using a sterile mortar and pestle in liquid nitrogen. Total gDNA was extracted from confirmed surface-sterilized nodules using an Isolate II plant DNA kit (Bioline, London UK) according to the manufacturer's protocol. The extracted DNA was visualized using 1.5% (wt/vol) agarose gel and quantified using a NanoDrop^TM^ 2000 UV-Vis spectrophotometer (Thermo Fischer Scientific Inc., California, USA). Samples with good quality DNA ranging from 1.7 to 2.1 based on A260/A280 nm were selected for 16S rDNA (bacteria) and internal transcribed spacer gene (ITS, fungi) sequencing.

Library preparation, PCR and sequencing were conducted at Macrogen Europe (Amsterdam, The Netherlands). Amplicon sequencing targeted the 16S rDNA gene (V3–V4; bacteria) and ITS gene regions (ITS1–ITS2; fungi). Fragment libraries were prepared using Herculase II Fusion DNA Polymerase Nextera XT Index V2 Kit. For 16S rDNA gene amplicon library preparation, we used the primer pair 341-F (CCTACGGGNGGCWGCAG) and 805-R (GACTACHVGGGTATCTAATCC) that flanks the V3–V4 dual region (Herlemann et al., 2011; Jalloh et al., 2024). The ITS gene region was amplified using the primer pair ITS1F (5′-CTTGGTCATTTAGAGGAAGTAA-3′) and ITS2R (5′-GCTGCGTTCTTCATCGATGC-3′) (Gardes and Bruns, 1993). Paired-end (2 × 300 bp) sequencing was performed using the MiSeq v3 sequencing kit (Illumina) and run on a MiSeq Illumina sequencing platform (Illumina, San Diego, USA), following the manufacturer's instructions.

### 2.4 Bioinformatics analyses

FASTQC (v0.11.6) was used to assess the quality of raw sequence reads (Wingett and Andrews, 2018). All subsequent analyses were conducted in R v4.3.0, using the RStudio v2023.06.0 interface (R Core Team, 2023). The sequencing libraries underwent further processing using the Divisive Amplicon Denoising Algorithm, DADA2 (v1.28.0), following the workflow proposed by Callahan et al. (2016) and Jalloh et al. (2024). Bacterial and fungal reads were trimmed and filtered with specific customized parameters, including a fixed length of 250 from 3′-end and 230 from 5'-end, respectively, using the “filterAndTrim” function with custom parameters maxN = 0, maxEE = c(2,5), truncQ = 2, (Schloss, 2020). Removal of low-quality DNA sequences enhanced merging accuracy in DADA2. Dereplication was carried out using the “derepfastq” function, and high-quality sequences were inferred from their associated unique amplicon sequence variants (ASVs), including singletons. Forward and reverse overlapping reads were merged using the “mergepairs” function. Spurious and chimeric ASVs were eliminated using “removebimeradenovo” function.

Taxonomy was assigned against pre-trained databases, including the SILVA database v138.1 (Quast et al., 2013) and UNITE ITS database v9.0 (Nilsson et al., 2019) using the “assigntaxonomy” function in a sequence table output (seqtab.nochim). Further analyses, including diversity assessment, visualization, and manipulation of the data, were performed using phyloseq v1.44.0 (McMurdie and Holmes, 2013), Tidyverse package v2.0.0 (Wickham et al., 2019), and Janitor package v2.2.0 (Firke, 2021). The taxonomy and ASV tables created two phyloseq objects for both bacteria and fungi. Bacterial and fungal ASVs were subjected to prevalence taxonomic subsetting to eliminate undesired sequences including plant-associated chloroplast, taxonomically unassigned ASVs at the genus level (NAs) (28 out of 514 which is ~5.08% for 16S rDNA and 206 out of 799, ~25.78% for ITS), mitochondrial sequences and singletons before downstream analysis using the “subset_taxa” function of phyloseq. Subsequent taxa were filtered using the “prune_taxa” function across all samples to retain only the most abundant ones. The two phyloseq objects were merged with the sample metadata, transformed into data frames, and underwent cumulative sum scaling using the metagmisc package (Mikryukov, 2020). The top 30 genera and species relative abundance bar plots were generated using the ggplot2 package v3.4.2 (Wickham, 2016). Relative abundance count data for the dominant genera and species were transformed into proportions and analyzed using the compositional total sum scaling (TSS) linear regression model (*P* < 0.05).

Alpha diversity (α-diversity) was calculated using the Microbiota process v1.9.3 based on sample ASV richness/profiles from a rarefied phyloseq object using; “*Chao1*,” “*Evenness*,” and “*Shannon*” diversity predictors (Xu et al., 2022; Jalloh et al., 2024). The resulting indices were subjected to a Shapiro-Wilk test to assess normality and visualized with boxplots using the “ggpubr” package v0.6.0 (Kassambara, 2020). Beta diversity (β-diversity) was computed using generalized Unifrac distances based on phylogeny, using the “phyloseq:ordinate” function (Lozupone and Knight, 2005; Chen et al., 2012). Resulting distances were clustered and ordinated using principal coordinates analysis (PCoA) biplots using the Vegan package v2.6.4 (Oksanen et al., 2012). Venn diagrams were generated using the “Venn Diagram” package v1.7.3 (Chen and Boutros, 2011; Jalloh et al., 2024) to illustrate shared and unique ASVs as influenced by study locations and the three *Desmodium* species at the species level. Permutational multivariate analysis of variance (PERMANOVA) was used to compare the microbial differences between the three *Desmodium* species and sample locations using the “adonis” function implemented in the “vegan” package v2.6.4 (Oksanen et al., 2012). Additionally, relative metabolic functional abundances were predicted for bacterial communities using phylogenetic investigation of communities by reconstruction of unobserved states (PICRUSt2 pipeline; v2.4.1) with metacyc as the reference database (https://metacyc.org/). The results were visualized through a heatmap generated using the “gplots” package v3.1.3 (Warnes et al., 2016) with unsupervised hierarchical clustering employing both Pearson and Spearman correlation coefficients. Lastly, statistical analysis of the obtained functions was performed using statistical analysis of taxonomic and functional profiles (STAMP; v2.1.3) software (Parks et al., 2014) and visualized using boxplots.

## 3 Results

### 3.1 Relative abundance of root-nodule microbiome in three *Desmodium* species

While all 24 16S rDNA samples passed the quality check, three ITS samples (one from each *Desmodium* species) were excluded due to low reads. Fungal profiling of amplicon sequencing of root-nodules from the three *Desmodium* species yielded 1,036,518 high-quality sequence reads, with a median of 43,236 and a mean of 43,188. Bacterial amplicon sequencing of the three *Desmodium* species root-nodules yielded 874,794 reads, with a median of 36,865 and a mean of 36,449. Bacterial reads ranged from 29,286 to 44,115, whereas fungal reads ranged from 33,196 to 60,966. From the 16S reads, 514 ASVs were identified, whereas 799 ASVs were obtained from the ITS reads. However, after quality control, filtering, and chimera removal (excluding non-fungal and non-bacterial sequences), we identified 393 fungal and 367 bacterial ASVs in the root-nodule samples of the three *Desmodium* species.

### 3.2 Composition and structure of the root-nodule microbiome across different *Desmodium* species and sampling locations

**Figure 2:**
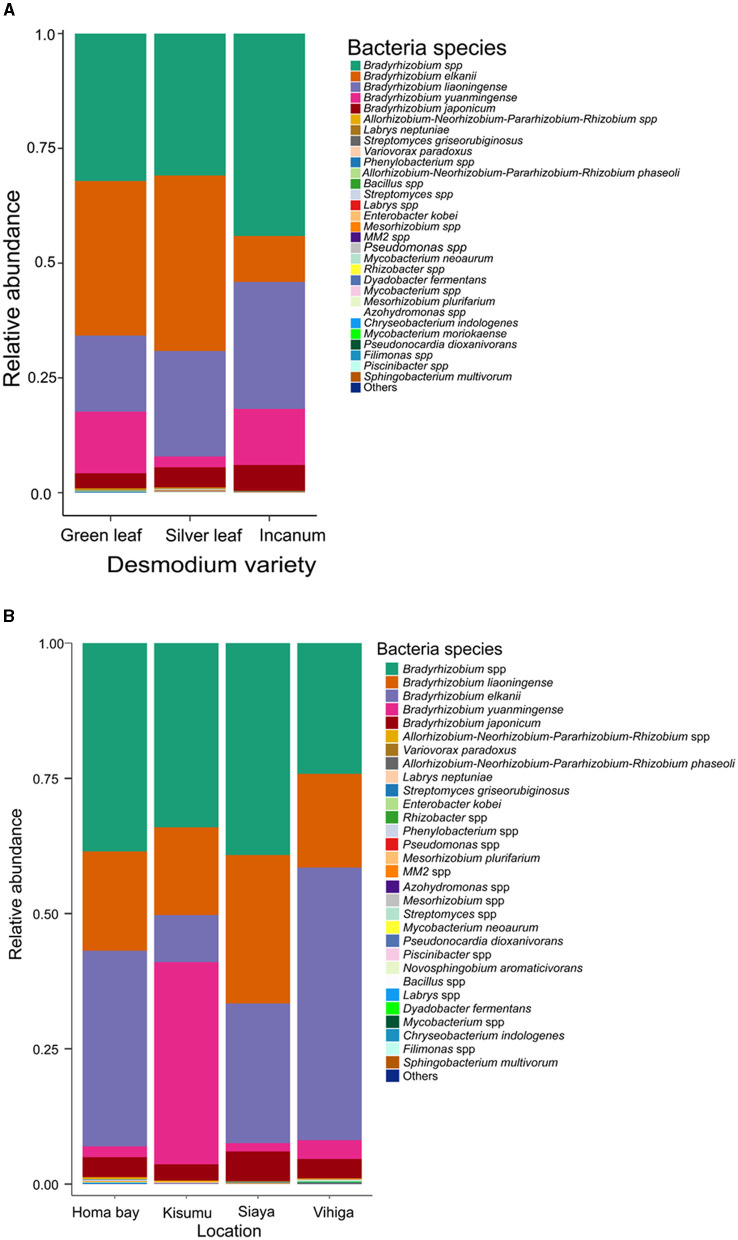
Barplots representing the relative abundance of the predominant bacterial amplicon sequence variants (ASVs) at the species level **(A)** across all the three *Desmodium* species; **(B)**, Barplots representing the relative abundance of the predominant bacterial amplicon sequence variants (ASVs) at the species level across all the four sampling locations. *Desmodium intortum* (Green leaf), *Desmodium uncinatum* (Silver leaf) and *Desmodium incanum* (incanum).

Over 98% of the sequences were identified as belonging to *Bradyrhizobium* species across all *Desmodium* species and locations (Supplementary Figures S1, S2 and Supplementary Tables S2, S3). Other bacterial species accounted for < 2% of the sequences obtained, although their distribution and presence varied among treatments. Within the *Bradyrhizobium* genus, several species were detected, namely *Bradyrhizobium elkanii, B*. *liaoningense, B*. *yuanmingense* and *B*. *japonicum* with varying abundances (Figure 2A, Supplementary Table S4). Interestingly, we only detected *Phenylobacterium* in AID, *Streptomyces* in GLD and SLD, *Bacillus* in GLD and *Enterobacter* in SLD (Supplementary Figure S1 and Supplementary Table S2). Moreover, the bacterial species *Mycobacterium neoaurum, Enterobacter kobei, Streptomyces griseorubiginosus* and *Variovorax paradoxus* were only observed in SLD. *Streptomyces griseorubiginosus, V. paradoxus, M. neoaurum*, and *E. kobei* were unique to SLD whereas *Labrys neptuniae* was only detected in GLD (Figure 2A, Supplementary Table S4). Besides, we only found *Acidothermus* and *Phenylobacterium* in Homabay, and *Variovorax* and *Mesorhizobium* in Siaya County whereas *Pseudomonas* and *Enterobacter* were unique to Vihiga County (Supplementary Figure S2 and Supplementary Table S4). However, some species were exclusively detected in some locations. For example, *Chryseobacterium indologenes* and *Dyadobacter fermentans* were only found in Vihiga, *Mycobacterium neoaurum* was only detected in Homabay whereas *Enterobacter kobei* was only found in Siaya County (Figure 2B, Supplementary Table S5).

Pairwise TSS analysis revealed no significant differences in the relative abundances of bacterial communities across the three *Desmodium* species (*P* > 0.05) (Supplementary Tables S6, S7). However, we found significant differences in the relative abundances of some bacteria genera across the sampling sites. For instance, *Bradyrhizobium* was more abundant in Vihiga than in Homabay (*P* = 0.018), while Homabay was composed of relatively more *Streptomyces* than Vihiga county (*P* = 0.015) (Supplementary Table S8). On the other hand, significant differences emerged in bacterial species across study locations, particularly in *B. japonicum* and *B. liaoningense* which were more abundant in Siaya than Homabay (*P* = < 0.001 and *P* = < 0.001 respectively) and Homabay than Vihiga (*P* = 0.005, *P* = 0.004) counties, respectively (Supplementary Table S9).

**Figure 3:**
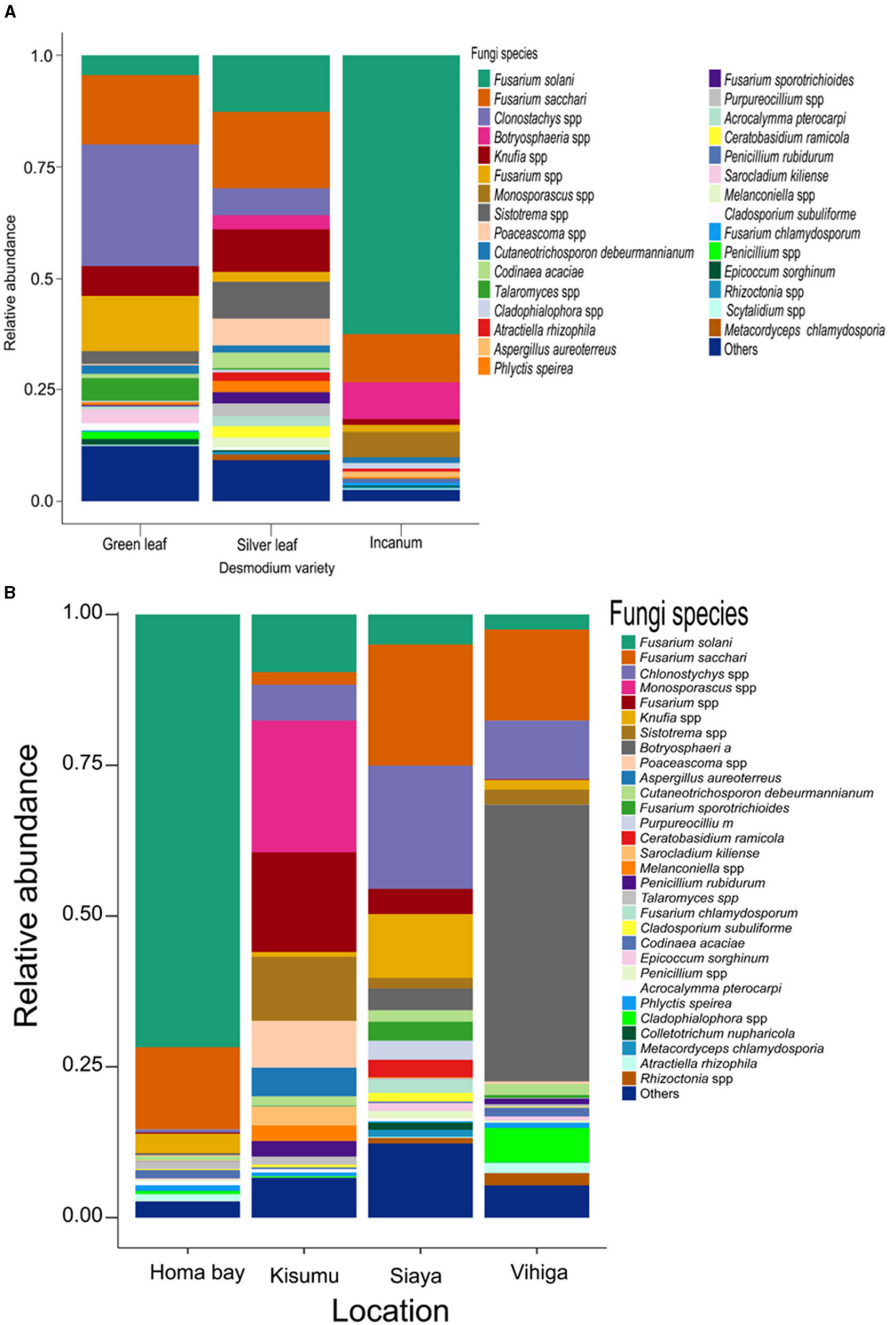
Barplots representing the relative abundance of the predominant fungal amplicon sequence variants (ASVs) at the species level **(A)** across all the three *Desmodium* species; **(B)**, Barplots representing the relative abundance of the predominant fungal amplicon sequence variants (ASVs) at the species level across all the four sampling locations. *Desmodium intortum* (Green leaf), *Desmodium uncinatum* (Silver leaf) and *Desmodium incanum* (incanum).

Analysis of root nodule mycobiome revealed *Fusarium* as the most abundant fungal genus in although with varying abundances across the treatments (33.40%;, 34.60%, and 76.10% respectively for GLD, SLD and AID and 85.40%, 28.30% and 35.00% respectively for Homabay, Kisumu and Siaya counties except in Vihiga where *Botryosphaeria* dominated at 45.40%) (Supplementary Figures S3, S4 and Supplementary Tables S10, S11). Other fungal genera such as *Penicillium, Talaromyces, Cladosporium*, and *Knufia* were detected at varying abundances across the three *Desmodium* species. Notably, *Sistotrema* and *Clonostachys* were only found in SLD and GLD, *Monosporascus* and *Cadophora* were only present in AID, *Purpureocillium* and *Scytalidium* were unique to SLD whereas *Sarocladium* was exclusively found in GLD (Supplementary Figure S3 and Supplementary Table S10). At the species level, *Fusarium solani, F*. *sacchari, Phlyctis speirea*, and *Epicoccum sorghinum* were present in all *Desmodium* species. Remarkedly, *Penicillium rubidurum* and *Aspergillus aureoterreus* were only found in AID whereas *Sarocladium kiliense* was only detected in GLD (Figure 3A, Supplementary Table S12). Fungal genera including *Phlyctis, Penicillium, Cladophialophora*, and *Knufia* were present in all counties at varying relative abundances. Interestingly, *Botryosphaeria, Atractiella, Epicoccum*, and *Purpureocillium* were abundant in all counties except Kisumu whereas *Sistotrema* was found in all counties except Homabay. Moreover, *Colletotrichum* and *Saitozyma* were only found in Siaya, while *Sarocladium* (mainly *Sarocladium kiliense*) was only detected in Kisumu (Supplementary Table S11). Fungal species such as *F*. *saccari, F*. *solari, Chlonostychys spp* and *Knufia spp* were variably abundant across all counties. However, *Penicillium rubidurum* was only found in Vihiga and Kisumu counties (Figure 3B, Supplementary Table S13).

Pairwise (TSS) log2 linear regression analysis indicated that *Clonostachys* was significantly more abundant in SLD than AID (*P* = 0.002) and GLD vs. AID (*P* = 0.003). *Botryosphaeria* was also significantly more abundant in SLD than GLD (*P* = 0.015) (Supplementary Table S14). Similar results were found at the species level where *Clonostachys* spp. was significantly more abundant in SLD (*P* = 0.002) and in GLD (*P* = 0.003) compared to AID (Supplementary Table S15). Across sampling locations, significant differences were recorded in the relative abundances of *Codinae* which was more abundant in Homabay than both Siaya (*P* = 0.045) and Kisumu (*P* = < 0.001) and also in Vihiga than Kisumu (*P* = 0.041). *Atractiella* was also more abundant in Homabay than in Kisumu (*P* = 0.043), and higher in Vihiga as compared to Kisumu (*P* = 0.022) (Supplementary Table S16). At the species level, *Codinaea acaciae* was more enriched in Vihiga than Homabay (*P* = < 0.001), while *Atractiella rhizophila* was relatively abundant in Vihiga than Kisumu (*P* = 0.022). *Fusarium* spp. was more abundant in Kisumu (*P* = 0.003) and Siaya (*P* = 0.022) than in Homabay County. Homabay had more *Fusarium* spp. than Vihiga (*P* = 0.005), while *Cladophialophora* spp. was significantly more abundant in Homabay than in Kisumu (*P* = 0.037) (Supplementary Table S17).

### 3.3 Alpha and beta diversity

**Figure 4:**
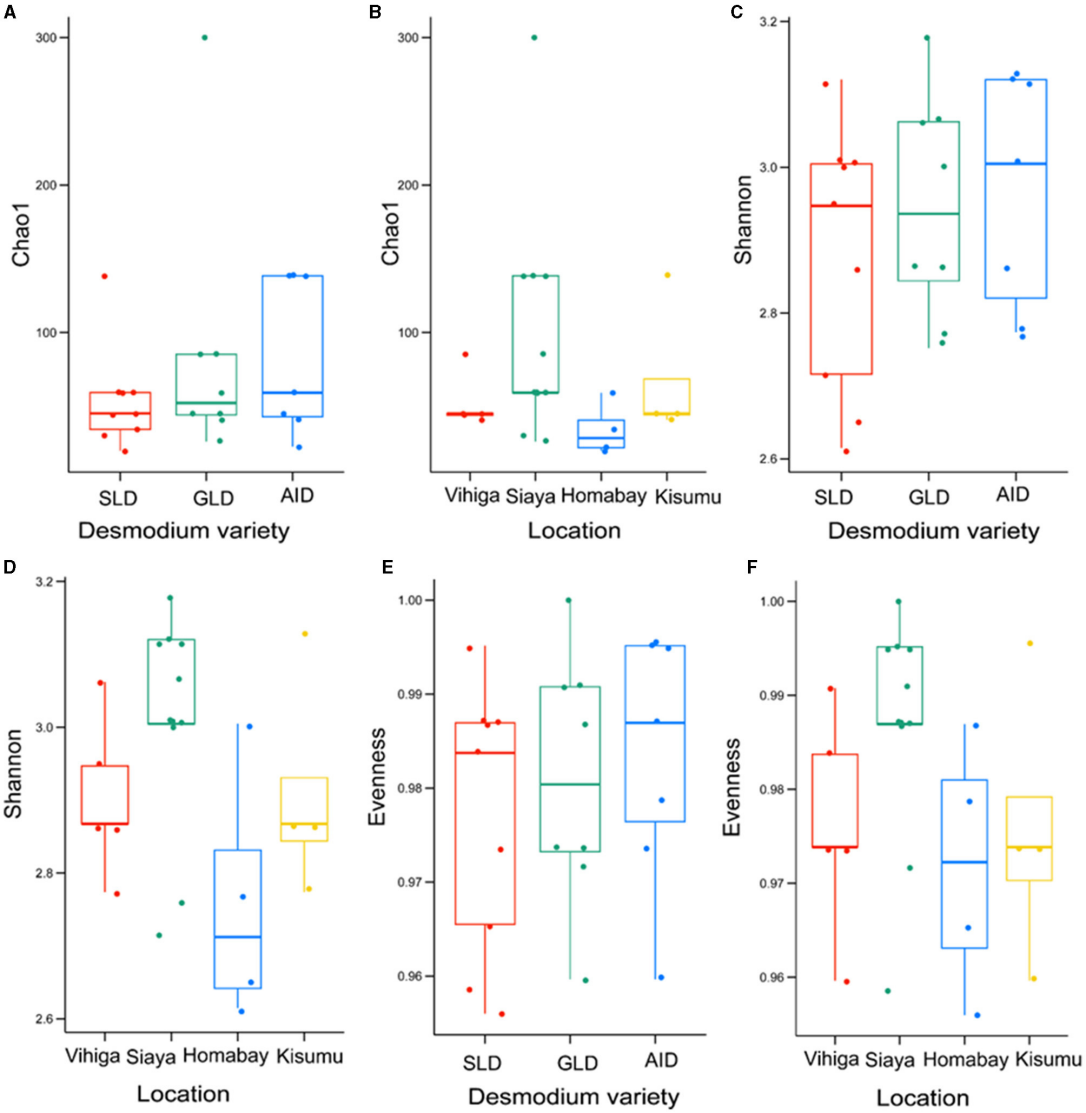
Alpha diversity estimates for bacterial species are represented using box plots, with **(A)** *Chao1* index for the three *Desmodium* species, Silverleaf desmodium (SLD), *Desmodium uncinatum*; Greenleaf desmodium (GLD), *Desmodium intortum*; and and African desmodium (AID), *Desmodium incanum*: **(B)** Influence of sampling locations on the *Chao1* index; **(C)** *Shannon* diversity index for the three *Desmodium* species; **(D)** Impact of sampling locations on the *Shannon* index; **(E)** *Evenness* of microbial diversity within the three *Desmodium* species; **(F)** Influence of sampling locations on microbial *Evenness*. In each plot, boxes represent the interquartile range (IQR) between the first and third quartiles, and the median is indicated by a horizontal line inside the box. Whiskers represent the lowest and highest values within the first and third quartiles, respectively.

There were no significant differences in bacterial species communities among the three *Desmodium* species as indicated by *Chao1, evenness* and *Shannon indices* (*Chao1 estimator, P* > 0.05 and *Shannon index P* > 0.05) (Figures 4A, C, E, Supplementary Table S18). Similarly, there were no significant differences in alpha diversity of bacteria ASVs across all studied locations, except for Homabay Vs Siaya (*Chao1 estimator, P* = 0.039 and *Shannon index, P* = 0.039) (Figures 4B, D, F, Supplementary Table S19).

**Figure 5:**
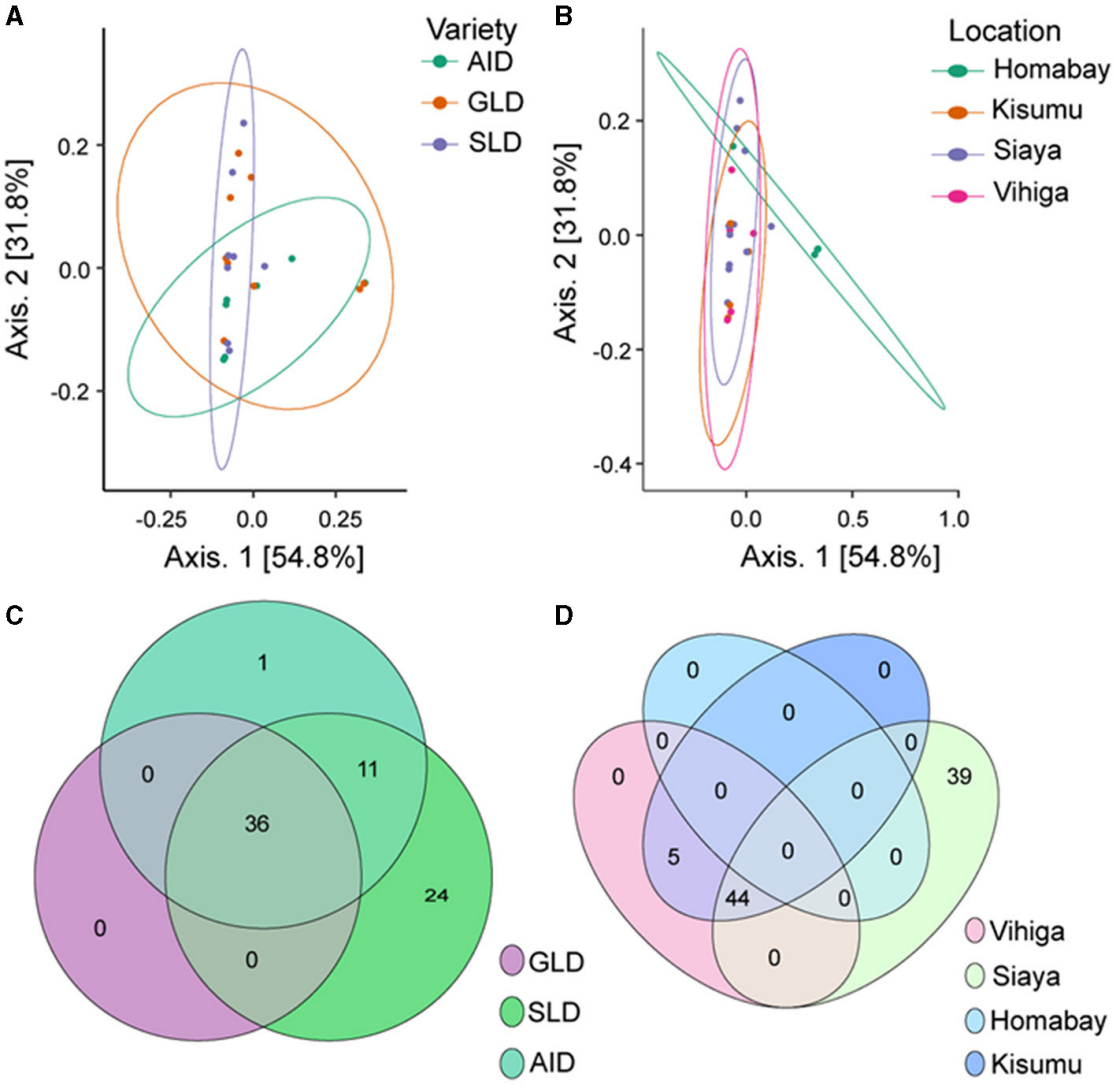
Beta diversity of bacterial species communities is presented through various visualization: **(A)** Principal coordinates analysis (PCoA) plot illustrating the influence of the three Desmodium species on bacterial species distribution; **(B)** PCoA plot depicting the impact of sampling location on bacterial communities; and **(C, D)** Venn diagrams showcasing the unique and commonly observed bacterial ASVs among the three *Desmodium* species and different sampling locations. In the Venn diagram, the number within the overlapping circles denotes the count of the shared ASVs. The three *Desmodium* species include *Desmodium intortum* (Greenleaf desmodium), GLD; *Desmodium uncinatum* (Silverleaf desmodium), SLD; and *Desmodium incanum* (African desmodium), AID.

β-diversity also showed that *Desmodium* species and study locations did not significantly influence the diversity of root-nodule bacterial communities, as they all clustered together. However, we observed a subtle clustering by *D. uncinatum* along axis 1 and Homabay County in axis 2, suggesting that both had unique bacterial communities (Figures 5A, B). The Venn diagram illustrated that 36 bacteria ASVs were commonly shared across all the three *Desmodium* species. SLD root-nodules had 24 unique bacterial ASVs, while AID had only one ASV and GLD had none (Figure 5C). AID and SLD had 11 uniquely shared bacterial ASVs, while SLD, GLD, and AID had no overlapping bacterial ASVs. Based on the sampling location, Siaya county had the most unique ASVs (39), whereas 44 ASVs were shared between Siaya and Kisumu counties (Figure 5D). PERMANOVA results showed a significant effect of sampling location on the root-nodule bacterial communities (*R*^2^ = 1.676, *P* = 0.003). However, *Desmodium* species did not significantly affect these bacterial communities (*R*^2^ = 0.299; *P* = 0.456) (Supplementary Table S20).

**Figure 6:**
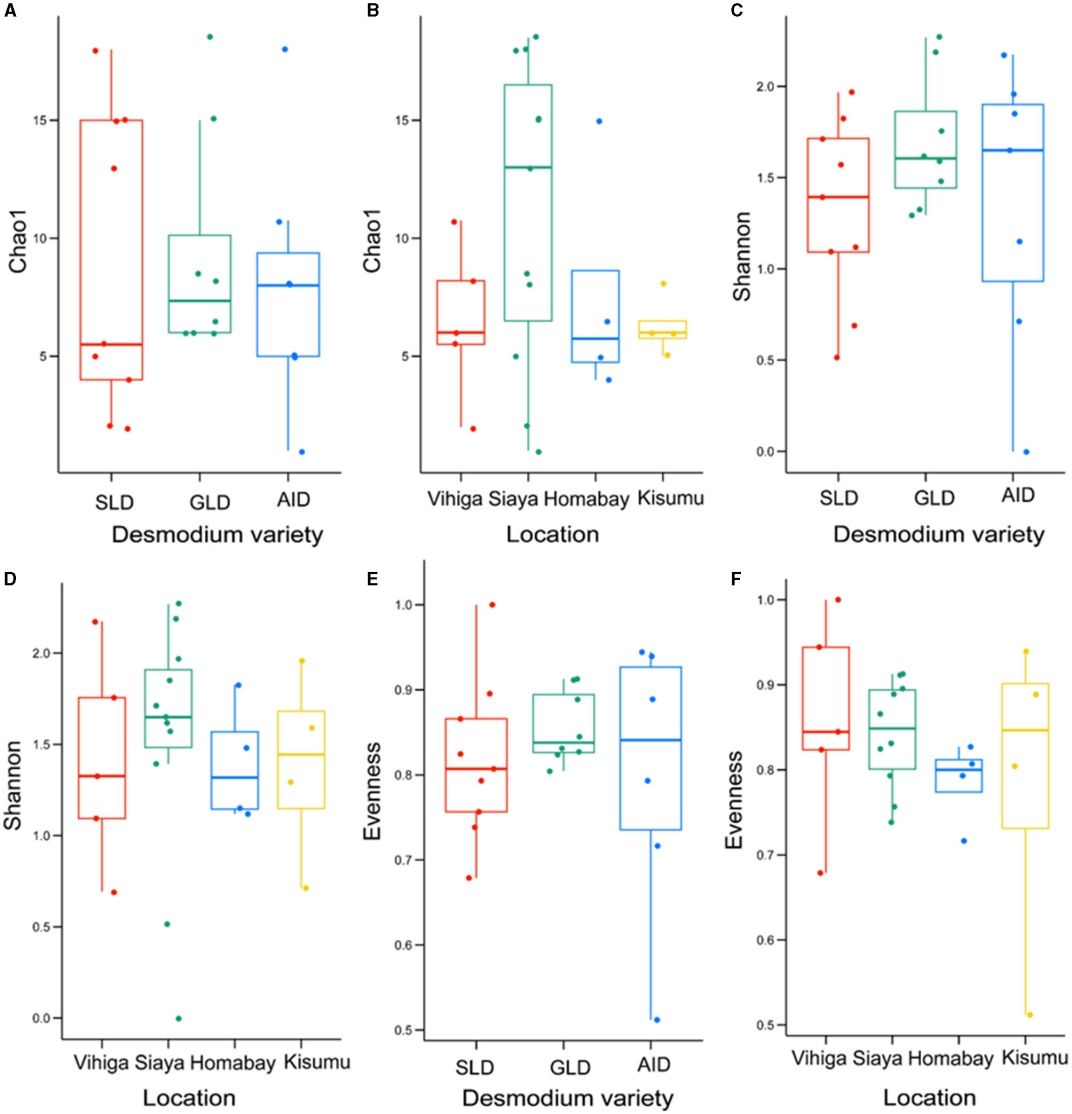
Alpha diversity estimates for fungal species are visually depicted using box-plots, with **(A)** *Chao1* index of the three *Desmodium* species, Silverleaf desmodium (SLD), *Desmodium uncinatum*; Greenleaf desmodium (GLD), *Desmodium intortum*; and African desmodium (AID), *Desmodium incanum*: **(B)** influence of sampling locations on the *Chao1* index; **(C)** *Shannon* diversity index of the three *Desmodium* species; **(D)** *Shannon* index as influenced by sampling location; **(E)** *Evenness* of microbial diversity within the three *Desmodium* species; **(F)** *Evenness* of microbial diversity on sampling locations. In each plot, boxes represent the interquartile range (IQR) between the first and third quartiles, and the median is indicated by a horizontal line inside the box. Whiskers represent the lowest and highest values within the first and third quartiles, respectively.

Fungal species did not significantly vary in *richness* and *evenness* among the three *Desmodium* species and sampling locations (*P* > 0.05) (Figure 6; Supplementary Tables S21, S22).

**Figure 7:**
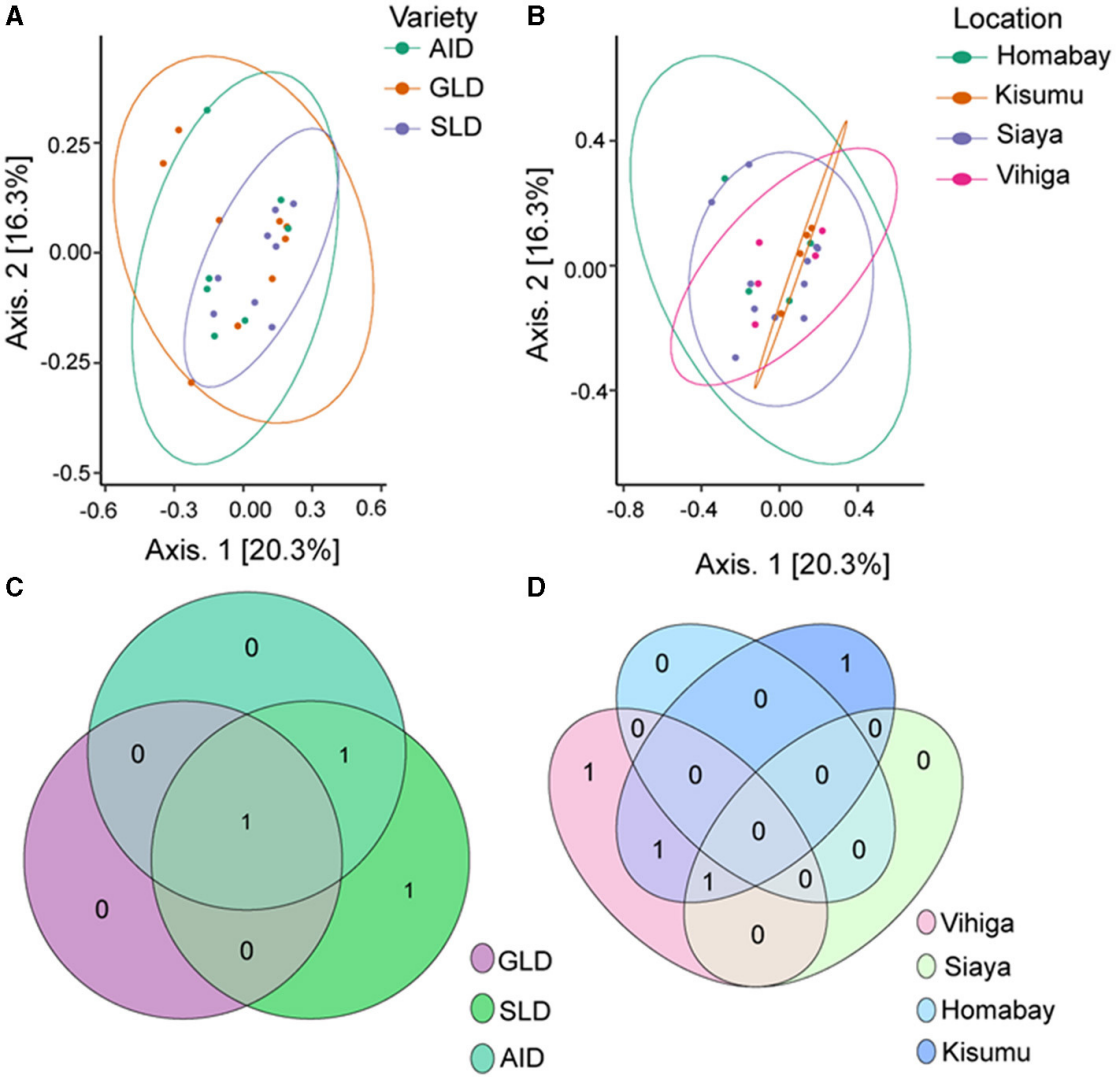
Beta diversity of fungal species communities is presented through several visualization: **(A)** Principal Coordinates Analysis (PCoA) plot of fungal species as influenced by the three *Desmodium* species; **(B)** PCoA plot depicting the impact of sampling locations on fungal communities; **(C, D)** Venn diagrams showcasing the unique and commonly observed fungal ASVs among the three *Desmodium* species and different sampling locations. The value in the overlapping circle represents the number of ASVs shared. The three *Desmodium* species include *Desmodium intortum* (Greenleaf desmodium), GLD; *Desmodium uncinatum* (Silverleaf desmodium), SLD; and *Desmodium incanum* (African desmodium), AID.

PERMANOVA analysis showed no significant effect of the three *Desmodium* species on fungal β-diversity (*R*^2^ = 0.908; *P* = 0.091) nor sampling location (*R*^2^ = 1.277; *P* = 0.118; Supplementary Table S23). Principal component analysis (PCoA) results showed clear clustering of root-nodule fungal communities in all the three *Desmodium* and sampling locations (Figures 7A, B). The Venn diagram revealed that SLD and AID had the most unique ASVs compared to GLD. However, one unique fungal ASV was shared amongst the three *Desmodium* species (Figure 7C). Kisumu and Vihiga counties each had only one unique fungal ASV compared to the rest, while only one fungal ASV was shared between Kisumu and Siaya counties (Figure 7D).

### 3.4 Predicted metabolic functions of bacterial occupants

**Figure 8:**
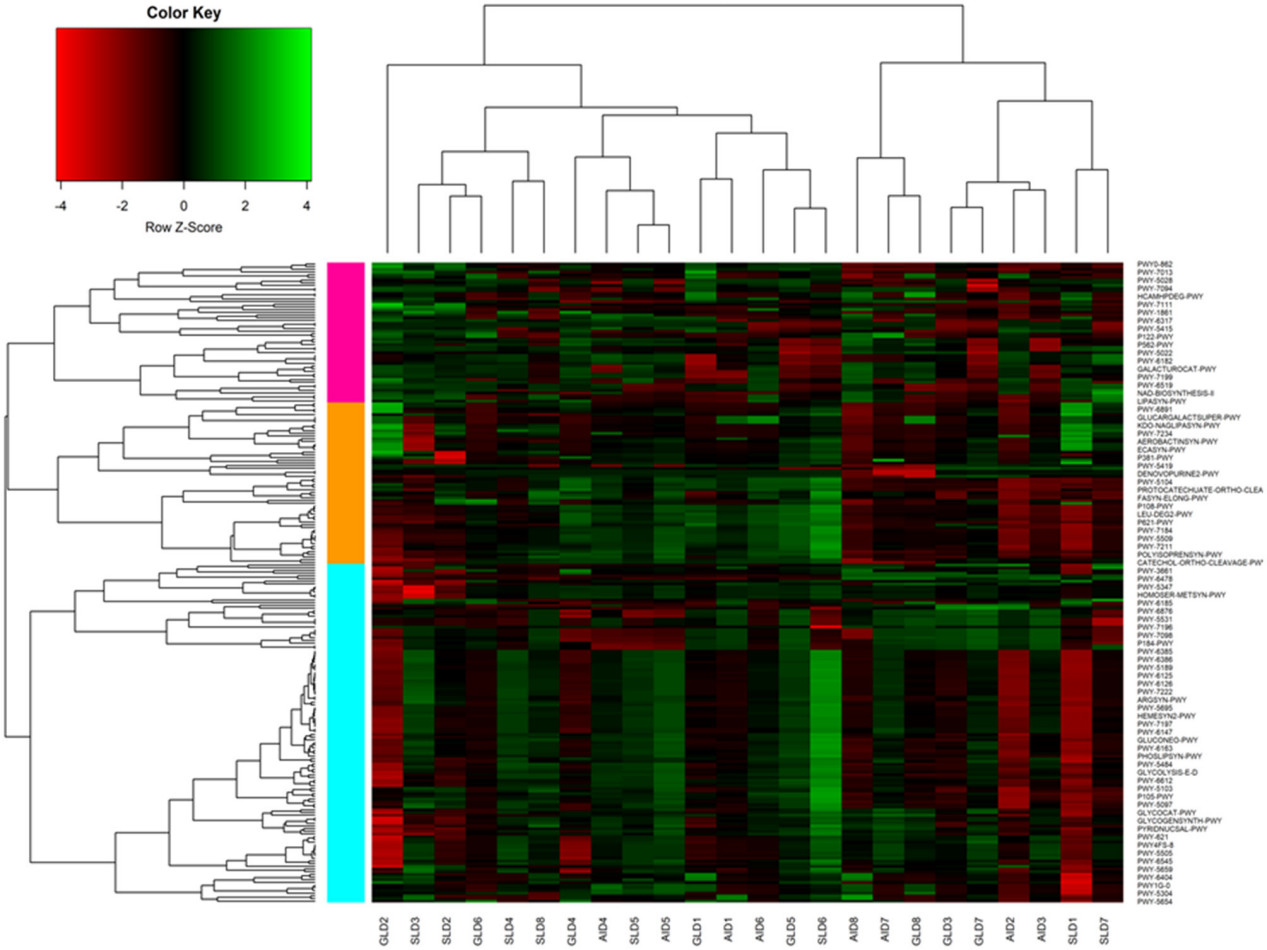
A cluster heatmap was generated to display the normalized relative abundances of moderately estimated functional profiles of root-nodule associated bacteria from the three *Desmodium* species. All functional categories were predicted using PICRUSt2. In the heatmap, colors reflect the z-score of the normalized relative abundances, with red indicating−4, and green indicating +4. The three *Desmodium* species include *Desmodium intortum* (Greenleaf desmodium), GLD; *Desmodium uncinatum* (Silverleaf desmodium), SLD; and *Desmodium incanum* (African desmodium), AID.

**Figure 9:**
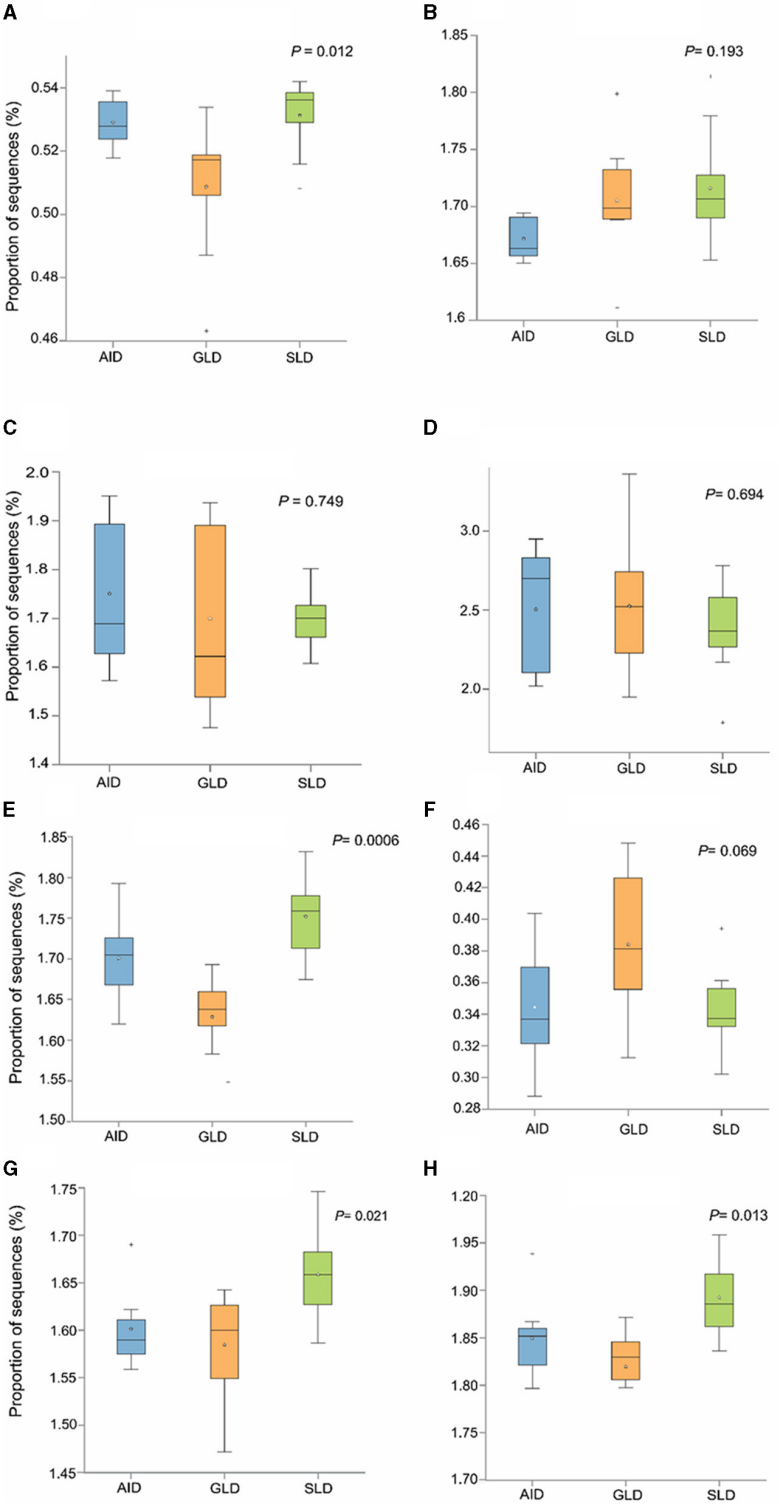
The abundance of bacterial sequences influencing selected metabolic pathways was assessed in relation to the three *Desmodium* species; *Desmodium intortum* (Greenleaf desmodium), GLD; *Desmodium uncinatum* (Silverleaf desmodium), SLD; and *Desmodium incanum* (African desmodium), AID): **(A)** the glycolysis-E-D pathway (Entner–Doudoroff pathway); **(B)** the TCA cycle (tricarboxylic acid cycle); **(C)** PENTOSE-P-PWY (pentose phosphate pathway); **(D)** protocatechuate-ortho-cleavage-PWY (protocatechuate degradation); **(E)** gluconeo-PWY (gluconeogenesis I); **(F)** leu-deg2-PWY (L-leucine degradation); **(G)** thresyn-PWY (superpathway of L-threonine biosynthesis); and (**H)** PWY-3001 **(**L-isoleucine biosynthesis).

Prediction of marker gene sequences associated with significant functional metabolic pathways (Figure 8) in the different root-nodules revealed that the essential pathways included fatty acid biosynthesis, phospholipid biosynthesis, the meta-cleavage pathway of catechol, carbon/energy biosynthesis, osmoregulation, carbohydrate biosynthesis, nitrogen related pathways, in particular, nucleoside and nucleotide salvage/biosynthesis, purine degradation pathways, amino acid biosynthesis, carrier/coenzyme/cofactor synthesis, vitamin synthesis, siderophore biosynthesis, and heme/leghaemoglobin production. Based on hierarchical clustering, the three *Desmodium* species root-nodule samples and study locations were grouped into two main clades. The first clade consisted of three subclades; the first subclade consisted of GLD only, the second subclade was predominantly associated with SLD and one GLD replicate, and the third subclade consisted of the three species, although AID was dominant. The second clade consisted of two major subclades, with the first subclade consisting of AID and GLD, whereas the second one depicted a patern distribution of the three *Desmodium* species. Eight metabolic pathways related to the metabolism of amino acid synthesis were selected. Despite the notable differences and shifts in important metabolic pathways among the three *Desmodium* species, significant differences were only observed in the proportion of sequences responsible for energy biosynthesis (glycolysis-E-D and gluconeo-PWY, *P* < 0.05) (Figure 9). A relatively higher proportion of sequences responsible for energy and amino acid biosynthesis were recorded in SLD (Figures 9A, B, E, H).

## 4 Discussion

The findings of this study revealed diverse NREs within the root-nodules of three *Desmodium* species, highlighting notable distinctions among them. The observation indicates that *Desmodium* plants may adapt to soils beyond their native habitat in South America, supported by the recruitment of diverse microbial communities. The diversity of microbes inhabiting the root-nodules of the three *Desmodium* species showed no significant correlation with the study location. However, variations in bacterial and fungal abundances across sampling locations could result from recurrent differences in climatic factors, including average annual temperatures, humidity and rainfall patterns, as reported by Pang et al. (2021). Remarkably, there was a shift in fungal communities compared to that of bacterial communities. The absence of significant differences in α and β diversity suggest that the overall composition of the *Desmodium* species root-nodules microbiome among the species was random and not influenced by either the *Desmodium* species or sampling location. Therefore, these findings contribute to our understanding of the relationships between *Desmodium* plants and their root-nodule microbiomes, offering insights into potential adaptations and ecological dynamics in diverse soil environments.

Our findings revealed that rhizobia, mainly belonging to the *Bradyrhizobium* genus, predominated as the primary occupants of *Desmodium* species root-nodules. This aligns with similar studies on pulse legumes such as *Trifolium* spp., *Pisum sativum, Cicer arietinum, Arachis hypogaea* and *Glycine max* (Hartman et al., 2017; Sharaf et al., 2019; Ilyas et al., 2022). The prevalence of *Bradyrhizobium* in *Desmodium* species root-nodules is noteworthy, considering its well-documented robust nitrogen-fixing capability in legumes within tropical and subtropical regions (Hurse and Date, 1992). This genus is a common microsymbiont in major pulses like soybean (*Glycine max*), peanuts (*Arachis hypogaea*), common bean (*Phaseolus vulgaris*), cowpea (*Vigna unguiculata*), and lima beans (*Phaseolus lunatus*) (Ormeño-Orrillo and Martínez-Romero, 2019; Muthini et al., 2020), with reported associations in various regions, including sub-Saharan Africa (Wekesa et al., 2022), China (Gu et al., 2007), Argentina (Toniutti et al., 2017) and Mexico (Parker, 2002). Our findings, however, differ from a study by Xu et al. (2016), which highlighted a higher occurrence of fast-growing rhizobia isolates (*Rhizobium, Mesorhizobium*, and *Pararhizobium* spp.) over *Bradyrhizobium* in wild *Desmodium sequax, Desmodium elegans, Desmodium gangeticum* and *Desmodium oldhamii*. Importantly, our study did not identify *Bradyrhizobium denitrificans*, previously reported in AID from Argentina (Toniutti et al., 2017), suggesting potential regional endemism among major rhizobial occupants (Xu et al., 2016). Beyond fixing nitrogen, *Bradyrhizobium* exhibits other beneficial traits, including acetylene reduction (Parker, 2002), antibiotic resistance (Hurse and Date, 1992), denitrification, and defense against fungal pathogens from the *Macrophomina* and *Sclerotium* genera (Chan, 2009). *Bacillus* spp., the second most abundant NRE known for enhancing heat and freezing tolerance, nodulation and phosphorous solubilization, was exclusively observed in GLD (Xu et al., 2014; Khalifa and Almalki, 2015; Tiwari et al., 2017). This endophyte, when co-inoculated with rhizobia, also serves as a biocontrol agent against *Rhizoctonia solani* and *Sclerotium rolfsii* by producing β-1, 3-glucanase enzymes that degrades their cell walls (Compant et al., 2005).

Another notable finding was the association of *Labrys neptuniae* with *Desmodium* species root-nodule, a bacterium belonging to the family Xanthobacteraceae. While this is the first report of *Labrys neptuniae* in *Desmodium* species, it has been previously isolated from root-nodules of wild *Acaciella* spp. in Mexico and *Entada phaseoloides* in Japan (Chou et al., 2007; Bai et al., 2011; Chávez-Ramírez et al., 2022). *Variovarax*, a genus capable of producing a broad spectrum of hydrolytic enzymes (like lipase, cellulase, and protease) and demonstrating various beneficial traits such as phosphorous solubilization, indoleacetic acid (IAA) synthesis, and heavy metal phytoremediation, was detected in SLD root-nodules (Aserse et al., 2013; De Almeida Lopes et al., 2016; Bessadok et al., 2020; Khan et al., 2023). However, the low occurrence of *Variovarax* in the studied *Desmodium* species suggests a lack of specific mechanisms to select these endophytes, limiting their potential role in enhancing plant growth (Mayhood and Mirza, 2021).

The study identified phytopathogenic bacteria, including *Enterobacter kobei, Macrophomina neoaurum, Pseudomonas* spp. and *Dyadobacter fermentans*, exclusively associated with SLD root-nodules. *Enterobacter kobei*, previously isolated from the root-nodules of wild *Hedysarum* genera, was reported to inhibit anthracnose causing *Colletotrichum musae* in bananas and improve zinc and phosphate solubilization in *Barleria lupulina* and lentils (Muresu et al., 2010; Damasceno et al., 2019). *Macrophomina neoaurum*, known for enhancing *in vitro* iron acquisition under iron-deficiency conditions via exochelin secretion, was also identified by Chan (2009). *Pseudomonas* spp. and *Chryseobacterium indologenes* reported as phosphate-solubilizing plant growth-promoting rhizobacteria (PGPR), IAA synthesis and biocontrol agents against *Phytophthora*, were associated with SLD root-nodules (Chelius and Triplett, 2001; Sang et al., 2018).

Fungal abundance within *Desmodium* species root-nodules uncovered a diverse assembly of filamentous fungi coexisting with bacterial occupants. These fungi, identified across all *Desmodium* species, colonize root-nodules by developing abruptly ending, thin-walled hyphae within cortical cells (Russell et al., 2002). Notably, pathogenic and non-pathogenic fungi have been identified in asymptomatic legumes, with potential mitigating effects by cohabiting bacteria (Da Silva et al., 2021). *Talaromyces* spp. was found across all three *Desmodium* species with higher abundance in GLD. This fungus is an antagonist against *Sclerotinia sclerotiorum* solubilizes phosphorous and produces IAA (Sahu et al., 2019). *Fusarium* was more abundant in the root-nodules of AID compared to SLD and GLD, a contrast to previous reports identifying it as a major fungal genus in the roots of forage legume Sainfoin (*Onobrychis viciifolia*) (Slabbert et al., 2023). While *Fusarium* has been isolated from various plant roots, including *Medicago* species and chickpeas (*Cicer arietinum* L.) (Lamprecht et al., 1988; Moparthi et al., 2021), its presence as *Desmodium* species root-nodule occupants is novel. SLD has also been reported to mediate the inhibition of *Fusarium oxysporum* growth (Were et al., 2022). Interestingly, the pathogen was not observed in all the *Desmodium* species, indicating that the mediation effect could be present in all three *Desmodium* species. *Fusarium solani* and *F. chlamydosporum* were previously isolated from the roots and nodules of *Medicago* species (Lamprecht et al., 1988), while *F. solani* and *F. sporotrichioides* were also isolated and identified from chickpeas (*Cicer arietinum*), dry pea (*Pisum sativum*), lentil (*Lens culinaris*) and *Axonopus compressus* roots (Moparthi et al., 2021). SLD and GLD exhibited enrichment of phytopathogenic microbes, including *F. sporotrichioides* and *F. sacharri*, known to cause root rot, lag-leaf sheath spot, wilt, and blights in legume plants, rice and potatoes compared to AID (Moparthi et al., 2021).

*Clonostachys* spp., observed in GLD and SLD, is recognized as a mycoparasitic fungus and biocontrol agent against pathogens, including *Rosellinia* root rot of cocoa by *Clonostachys byssicola* and banana crown rot by *C. byssicola* (Krauss et al., 2013). It also acts as an antagonist to *Phytophthora palmivora* (Krauss et al., 2013), *Moniliophthora roreri* (Harry et al., 2003), and *F. graminearum* (Nygren et al., 2018). *Clonostachys rosea*, secretes secondary metabolites with biotic activity against *Sclerotinia sclerotiorum* (Cabrera et al., 2011). *Monosporascus* spp., reported in higher relative abundance in GLD, has been previously isolated from the roots of xerophytic shrubs and as an endophyte of *Astragalus adsurgens*, contributing to soil organic carbon accumulation (Zuo et al., 2022). *Sistotrema* spp. also found in GLD and SLD, has conferred resilience to nutrient deficiency and drought stress in blueberries (*Vaccinium corymbosum*) (Ye et al., 2023). *Metacordyceps chlamydosporia*, a nematophagous fungal endophyte isolated from soybean root tips, (Strom et al., 2020) was only detected in SLD and GLD. *Acrocalymma, Atractiella rhizophila, Serendipita, Cadophora* and *Melanconiella* were selectively identified in the three *Desmodium* species root-nodules. *Acrocalymma* and *Melanconiella* were only observed in GLD and SLD, *Atractiella rhizophila* only observed in SLD and AID, and *Cadophora* only observed in AID. These microbes are classified as dark septate root endophytes (DSEs), which, through strain-dependent symbiotic associations with plants, enhance host plant tolerance to various environmental stresses, such as water deficit and salt stress (Farias et al., 2020; He et al., 2021). *Melanconiella* spp. and *A. rhizophila* have been reported to improve nitrogen and phosphorous nutrition in cowpeas under elevated salinity (Bonito et al., 2017; Farias et al., 2020; Gama et al., 2020; Muazzam and Darah, 2020). To the best of our knowledge, this is the first report of DSEs in *Desmodium* species root-nodules, although their agroecological services still need further study.

*Penicillium rubidurum, Cladosporium subuliforme, Ceratobasidium ramicola* and *Sarocladium kiliense* had varied relative abundance across the root-nodules of the three *Desmodium* species. *Ceratobasidium ramicola*, which exhibits both phytopathogenic and endophytic characteristics and confers antibiotic resistance to plants (Muazzam and Darah, 2020), was only reported in SLD. *Sarocladium kiliense* is only observed in GLD and has been reported as a *Brachiaria* endophyte conferring resistance against pathogenic *Sclerotinia sclerotiorum* (Gama et al., 2020). *Cladosporium subuliforme*, which was more relatively abundant in GLD than SLD and AID, has been identified as a *Diaphorina citri* entomopathogen (Wang et al., 2023). *Penicillium rubidurum*, observed only in AID, has been isolated from soil in Korea (Adhikari et al., 2017) and Colorado cropping soils, respectively, with biocontrol activity against phytopathogenic *Penicillium expansum* and in the roots of *Artemisia annua* (Yuan et al., 2011).

The root-nodules in our study displayed a notable potential for robust nitrogen fixation, attributable to the higher abundance of rhizobia. However, diverse NREs, suggested potential metabolic functional variances that warrant further exploration. In the context of legume symbiosis, substantial energy inputs are essential, with a critical reliance on carbon sources supplied by legume host cells for effective nodulation and nitrogen fixation (Makoudi et al., 2018). Here, we identified upregulated metabolic functions for rhizosphere fitness, including carbon metabolism, carbohydrate metabolism, and amino acid biosynthesis. Carbon metabolism is vital as it facilitates the transfer of photosynthates, primarily carbohydrates, from plant leaves to root-nodules for cellular respiration (Liu et al., 2018). Our findings indicate the involvement of sucrose invertase in sucrose degradation, as well as the aromatic degradation of protocatechuate and a meta-cleavage pathway of catechol, vanillin and vanillate (C1 Compounds). These processes are identified as characteristic features of *Bradyrhizobium japonicum* USDA110, and *Rhizobium leguminosarum* in pea (*Pisum sativum*) (Sudtachat et al., 2009; Garcia-Fraile et al., 2015; Jalloh et al., 2024). Moreover, the super pathway of D-glucarate and D-galactarate catabolism was reportedly enhanced in *Burkholderia*-*Caballeronia*-*Paraburkholderi* spp. Following this, carbon sources are transported across the bacteroid and peribacteroid membranes, entering the tricarboxylic acid cycle (TCA), glycolysis, and Entner-Doudoroff pathways for glucose catabolism (Liu et al., 2018). The Entner-Doudoroff pathway also aids *Rhizobium* spp. in enduring sulfur-abundant soils by breaking down abundant phototroph-derived carbohydrates (Li et al., 2020).

In terms of siderophore biosynthesis, we observed higher upregulation in SLD compared to AID and GLD, potentially indicating the presence of more microorganisms in SLD producing high-affinity Fe (III)-chelating compounds. Fatty acid and lipid biosynthesis pathways play a role in generating fatty acids and other lipids while converting nutrient-derived carbons into fatty acids (Chandel, 2021). Amino acid biosynthesis regulates ammonium assimilation and asparagine synthesis (Okumoto and Pilot, 2011). Vitamin biosynthesis is essential for various metabolic processes inside root-nodules and transporting selected decarboxylases, carboxylases and transcarboxylases. Examples of vitamins include thiamine, vitamin B12, and biotin production by *R. leguminosarum* and *R. etli* (Guillén-Navarro et al., 2005). Additionally, protocatechuate catabolism has been shown to increase the fitness of *R. leguminosarum* in the rhizosphere by providing a constant supply of dicarboxylates to the bacteroids (Strodtman et al., 2018). PICRUSt predicts functions of multiple 16S genes within genera using defined marker-gene metagenomic reference data. However, the lack of direct metagenome data on root-nodule microbiomes limits comprehensive prediction of functional gene categories within the root nodule microbiomes. Therefore, further research is necessary to delineate the 16S root-nodule metagenome to enable comprehensive prediction of functional gene categories within the root-nodule microbiomes.

## 5 Conclusion

This study unveiled diverse microbial communities co-inhabiting root-nodules of three *Desmodium* species used in push-pull cropping systems with *Bradyrhizobium* spp. bacterial symbionts being predominant within the plant organ. Additionally, it reveals the coexistence of diazotrophic bacteria with non-nodulating fungi within *Desmodium* root-nodules, although their precise functions remain unclear warranting further investigation. Our findings further reveal no apparent differences in the microbiomes of AID, SLD, and GLD reflecting similarities in symbiotic partner selection and adaptability among the three *Desmodium* species to the local environment, leading to convergence in their root-nodule microbiomes. Microbiome diversity was generally higher in root-nodules collected from Siaya county compared to those collected from Homabay, Vihiga, and Kisumu counties. Further assessment of soil geochemical properties and temporal climatic fluctuations from all sampling locations is necessary to determine if and how these abiotic factors contribute to the observed variation in microbial diversity. The findings of our study also provide insights into other potential functions of *Desmodium* root-nodule-associated microbiome such as carbon metabolism and amino acid biosynthesis. These functions are potentially involved in the endosymbiotic relationship while also conferring rhizosphere fitness. Thus, we recommend that future research investigates and harnesses the microbes responsible for these novel functions for improved soil health, plant growth and agroecosystem functioning.

## Data availability statement

The raw metagenome amplicon sequences data from the root-nodules of the three Desmodium species were submitted to NCBI Sequence Read Archives (SRA) under BioProject accession number PRJNA1102675 for the 16S dataset. Specifically, the 16S (V3-V4) metagenome data were assigned Biosample accession numbers SAMN41026867-SAMN41026890, while the ITS (ITS1-ITS2) metagenome data were registered under BioProject PRJNA1102689 with accession numbers SAMN41027037-SAMN41027060. GPS coordinates was provided in the Additional file 1. Additionally, we also provided the R scripts for data analysis along with all the necessary input files, Additional files 2A, B.

## Author contributions

IA: Data curation, Formal analysis, Investigation, Methodology, Writing – original draft, Writing – review & editing. GA: Data curation, Formal analysis, Methodology, Writing – review & editing. SN: Conceptualization, Investigation, Writing – review & editing. AJ: Data curation, Formal analysis, Methodology, Writing – review & editing. JM: Data curation, Formal analysis, Investigation, Methodology, Writing – review & editing. FC: Investigation, Writing – review & editing. FK: Writing – review & editing. ZK: Supervision, Writing – review & editing. SS: Funding acquisition, Supervision, Writing – review & editing. TD: Supervision, Writing – review & editing. DM: Conceptualization, Data curation, Formal analysis, Investigation, Methodology, Writing – original draft, Writing – review & editing.

## References

[B1] Adhikari M. Kim S. Kim H. Lee H. Lee Y. (2017). Sixteen new records of Ascomycetes from crop field soil in Korea. Kor. J. Mycol. 44, 271–288. 10.4489/KJM.2016.44.4.271

[B2] Aserse A. A. Räsänen L. A. Aseffa F. Hailemariam A. Lindström K. (2013). Diversity of sporadic symbionts and nonsymbiotic endophytic bacteria isolated from nodules of woody, shrub, and food legumes in Ethiopia. Appl. Microbiol. Biotechnol. 97, 10117–10134. 10.1007/s00253-013-5248-4 24196581

[B3] Bai Y. D'Aoust F. Smith D. L. Driscoll B. T. (2011). Isolation of plant-growth-promoting *Bacillus* strains from soybean root nodules. Can. J. Microbiol. 48, 230–238. 10.1139/w02-014 11989767

[B4] Bessadok K. Navarro-Torre S. Pajuelo E. Mateos-Naranjo E. Redondo-Gómez S. Caviedes M. Á.. (2020). The acc-deaminase producing bacterium *Variovorax* sp. ct7.15 as a tool for improving *Calicotome villosa* nodulation and growth in arid regions of Tunisia. Microorganisms 8:541. 10.3390/microorganisms8040541 32283666 PMC7232455

[B5] Bonito G. Hameed K. Toome-Heller M. Healy R. Reid C. Liao H. L.. (2017). *Atractiella rhizophila*, sp. nov., an endorrhizal fungus isolated from the Populus root microbiome. Mycologia 109, 18–26. 10.1080/00275514.2016.1271689 28402786

[B6] Cabrera G. Gozzo F. C. Eberlin M. N. Godeas A. (2011). *Clonostachys rosea* BAFC3874 as a *Sclerotinia sclerotiorum* antagonist: mechanisms involved and potential as a biocontrol agent. J. Appl. Microbiol. 110, 1177–1186. 10.1111/j.1365-2672.2011.04970.x 21385290

[B7] Callahan B. J. Sankaran K. Fukuyama J. A. McMurdie P. J. Holmes S. P. (2016). Bioconductor workflow for microbiome data analysis: from raw reads to community analyses. F1000 Res. 5:1492. 10.12688/f1000research.8986.2 27508062 PMC4955027

[B8] Carlström C. I. Field C. M. Bortfeld-Miller M. Müller B. Sunagawa S. Vorholt J. A.. (2019). Synthetic microbiota reveal priority effects and keystone strains in the *Arabidopsis phyllosphere*. Nat. Ecol. Evol. 3, 1445–1454. 10.1038/s41559-019-0994-z 31558832 PMC6774761

[B9] Chan K. G. (2009). Exochelin production in *Mycobacterium neoaurum*. Int. J. Mol. Sci. 10, 345–353. 10.3390/ijms10010345 19333449 PMC2662466

[B10] Chandel N. S. (2021). Lipid metabolism. Cold Spring Harbor Perspectives in Biol. 13:40576. 10.1101/cshperspect.a040576 34470787 PMC8411952

[B11] Chávez-Ramírez B. Larios-Serrato V. Estrada-de los Santos P. (2022). Draft genome sequence of *Labrys okinawensis*, Isolated from *Acaciella* sp. nodules in Mexico. Microbiol. Res. Announc. 11, 10–11. 10.1128/mra.00732-22 36342286 PMC9753675

[B12] Chelius M. K. Triplett E. W. (2001). The diversity of archaea and bacteria in association with the roots of *Zea mays* L. Microb. Ecol. 41, 252–263. 10.1007/s002480000087 11391463

[B13] Chen H. Boutros P. C. (2011). Venn diagram: a package for the generation of highly-customizable Venn and Euler diagrams in R. BMC Bioinf. 12:35. 10.1186/1471-2105-12-35 21269502 PMC3041657

[B14] Chen J. Bittinger K. Charlson E. S. Hoffmann C. Lewis J. Wu G. D.. (2012). Associating microbiome composition with environmental covariates using generalized UniFrac distances. Bioinformatics 28, 2106–2113. 10.1093/bioinformatics/bts342 22711789 PMC3413390

[B15] Cheruiyot D. Chidawanyika F. Midega C. A. Pittchar J. O. Pickett J. A. Khan Z. R.. (2021). Field evaluation of a new third generation push-pull technology for control of *Striga* weed, stemborers, and fall armyworm in western Kenya. Exp. Agric. 57, 301–315. 10.1017/S0014479721000260

[B16] Cheruiyot D. Midega C. Van den Berg J. Pickett J. Khan Z. (2018). Suitability of *Brachiaria* grass as a trap crop for management of *Chilo partellus*. Entomol. Exp. Appl. 166:12651. 10.1111/eea.12651

[B17] Chou Y. J. Elliott G. N. James E. K. Lin K. Y. Chou J. H. Sheu S. Y.. (2007). *Labrys neptuniae* sp. nov., isolated from root nodules of the aquatic legume *Neptunia oleracea*. Int. J. Syst. Evol. Microbiol. 57, 577–581. 10.1099/ijs.0.64553-0 17329788

[B18] Compant S. Duffy B. Nowak J. Clément C. Barka E. A. (2005). Use of plant growth-promoting bacteria for biocontrol of plant diseases: principles, mechanisms of action, and future prospects. Appl. Environ. Microbiol. 71, 4951–4959. 10.1128/AEM.71.9.4951-4959.2005 16151072 PMC1214602

[B19] Core Team R. (2023). R: A Language and Environment for Statistical Computing. Vienna: R Foundation for Statistical Computing.

[B20] Curtis T. P. Sloan W. T. Scannell J. W. (2002). Estimating prokaryotic diversity and its limits. Proc. Natl. Acad. Sci. USA. 99, 10494–10499. 10.1073/pnas.142680199 12097644 PMC124953

[B21] Da Silva D. Bomfim V. B. Sena C. S. G. Santos P. T. S. Mattos J. C. S. Gava W.. (2021). *Vigna* spp. root-Nodules harbor potentially pathogenic fungi controlled by co-habiting bacteria. Curr. Microbiol. 78, 1835–1845. 10.1007/s00284-021-02455-3 33772620

[B22] Dakora F. D. (2015). Lumichrome: a bacterial signal molecule influencing plant growth. Biol. Nitrogen Fixation 2, 389–396. 10.1002/9781119053095.ch3817953546

[B23] Damasceno C. L. Duarte E. A. A. dos Santos L. B. P. R. Oliveira de Jesus T. A. S. de Oliveira F. N. Soares A. C. F.. (2019). Postharvest biocontrol of anthracnose in bananas by endophytic and soil rhizosphere bacteria associated with sisal (*Agave sisalana*) in Brazil. Biol. Control 137:104016. 10.1016/j.biocontrol.2019.104016

[B24] De Almeida Lopes D. Carpentieri-Pipolo K. B. Oro V. Stefani Pagliosa T. H. E. Degrassi G. (2016). Culturable endophytic bacterial communities associated with field-grown soybean. J. Appl. Microbiol. 120:13046. 10.1111/jam.13046 26744016

[B25] Drinkwater L. E. Midega C. A. Awuor R. Nyagol D. Khan Z. R. (2021). Perennial legume intercrops provide multiple belowground ecosystem services in smallholder farming systems. Agric. Ecosyst. Environ. 320:107566. 10.1016/j.agee.2021.107566

[B26] Farias G. C. Nunes K. G. Soares M. A. de Siqueira D. Lima K. A. Neves W. C.. (2020). Dark septate endophytic fungi mitigate the effects of salt stress on cowpea plants. Braz. J. Microbiol. 51, 243–253. 10.1007/s42770-019-00173-4 31656023 PMC7058810

[B27] Firke S. (2021). Simple Tools for Examining and Cleaning Dirty Data. Available online at: https://CRAN.R-project.org/package=janitor (accessed February 10, 2024).

[B28] Fukuda D. Ohnuki N. Ohnuki T. (2022). Bacterial diversity of root nodule and rhizosphere soil samples of green soybean (Edamame) in Japan. Microbiol. Res. Announc. 11, e01114–e01121. 10.1128/mra.01114-21 35112903 PMC8812318

[B29] Gama D. D. S. Santos Í. A. F. M. De Abreu D. De Medeiros L. M. Duarte F. H. V. W. F. Cardoso P. G. (2020). Endophytic fungi from *Brachiaria* grasses in Brazil and preliminary screening of *Sclerotinia sclerotiorum* antagonists. Sci. Agric. 77:210. 10.1590/1678-992x-2018-0210

[B30] Garcia-Fraile P. Seaman J. C. Karunakaran R. Edwards A. Poole P. S. Downie J. A.. (2015). Arabinose and protocatechuate catabolism genes are important for growth of *Rhizobium leguminosarum biovar* viciae in the pea rhizosphere. Plant Soil 390, 251–264. 10.1007/s11104-015-2389-5 26166901 PMC4495286

[B31] Gardes M. Bruns T. D. (1993). ITS primers with enhanced specificity for basidiomycetes-application to the identification of mycorrhizae and rusts. Mol. Ecol. 2, 113–118. 10.1111/j.1365-294X.1993.tb00005.x 8180733

[B32] Gu J. Wang E. T. Chen W. X. (2007). Genetic diversity of rhizobia associated with *Desmodium* species grown in China. Lett. Appl. Microbiol. 44, 286–292. 10.1111/j.1472-765X.2006.02071.x 17309506

[B33] Guillén-Navarro K. Encarnación S. Dunn M. F. (2005). Biotin biosynthesis, transport and utilization in rhizobia. FEMS Microbiol. Lett. 246, 159–165. 10.1016/j.femsle.2005.04.020 15899401

[B34] Hannula S. E. Heinen R. Huberty M. Steinauer K. Long D. Jongen J. R.. (2021). Persistence of plant-mediated microbial soil legacy effects in soil and inside roots. Nat. Commun. 12:5686. 10.1038/s41467-021-25971-z 34584090 PMC8478921

[B35] Harry C. Keith A. Sarah E. (2003). Endophytes and mycoparasites associated with an indigenous forest tree, *Theobroma gileri*, in Ecuador and a preliminary assessment of their potential as biocontrol agents of cocoa diseases. Mycol. Prog. 2, 149–160. 10.1007/s11557-006-0053-4

[B36] Hartman K. van der Heijden M. G. A. Roussely-Provent V. Walser J. C. Schlaeppi K. (2017). Deciphering composition and function of the root microbiome of a legume plant. Microbiome 5, 1–13. 10.1186/s40168-016-0220-z 28095877 PMC5240445

[B37] He C. Wang W. Hou J. Li X. (2021). Dark septate endophytes isolated from wild licorice roots grown in the desert regions of northwest China enhance the growth of host plants under water deficit stress. Front. Microbiol. 12:522449. 10.3389/fmicb.2021.522449 34248857 PMC8260703

[B38] Herlemann D. P. Labrenz M. Jürgens K. Bertilsson S. Waniek J. J. Andersson A. F.. (2011). Transitions in bacterial communities along the 2000 km salinity gradient of the Baltic Sea. ISME J. 5, 1571–1579. 10.1038/ismej.2011.41 21472016 PMC3176514

[B39] Hurse L. S. Date R. A. (1992). Competitiveness of indigenous strains of *Bradyrhizobium* on *Desmodium intortum* CV greenleaf in three soils of South East Queensland. Soil Biol. Biochem. 24, 41–50. 10.1016/0038-0717(92)90240-X

[B40] Ilyas N. Yang Y. Zhang C. Singh R. P. Yu Q. Yuan Y.. (2022). Temporal dynamics and variation in the alfalfa root nodule and rhizosphere microbial communities of coastal sand and lawn soil. J. Plant Interact. 17, 173–182. 10.1080/17429145.2021.2024899

[B41] Jalloh A. A. Khamis F. M. Yusuf A. A. Subramanian S. Mutyambai D. M. (2024). Long-term push–pull cropping system shifts soil and maize-root microbiome diversity paving way to resilient farming system. BMC Microbiol. 24:92. 10.1186/s12866-024-03238-z 38500045 PMC10946131

[B42] Jalloh A. A. Njeru E. M. Maingi J. M. (2020). Potential of native rhizobia isolates to improve production of legume crops in small holder farms. Biosci. Res. 17, 1498–1510. Available online at: https://ssrn.com/abstract=3653170

[B43] Jing L. Jia-Min A. Xiao-Dong L. Ying-Ying J. Chao-Chao Z. Rui-Hua Z.. (2022). Environmental filtering drives the establishment of the distinctive rhizosphere, bulk, and root nodule bacterial communities of *Sophora davidii* in hilly and gully regions of the Loess Plateau of China. Front. Microbiol. 13:945127. 10.3389/fmicb.2022.945127 35935225 PMC9355530

[B44] Kassambara A. (2020). ggpubr: ‘ggplot2' Based Publication Ready Plots. R package version 0.4.0. Available online at: https://CRAN.R-project.org/package=ggpubr

[B45] Khalifa A. Y. Z. Almalki M. A. (2015). Isolation and characterization of an endophytic bacterium, *Bacillus megaterium* BMN1, associated with root-nodules of *Medicago sativa* L. growing in Al-Ahsaa region, Saudi Arabia. Ann. Microbiol. 65, 1017–1026. 10.1007/s13213-014-0946-4

[B46] Khan N. Humm E. A. Jayakarunakaran A. Hirsch A. M. (2023). Reviewing and renewing the use of beneficial root and soil bacteria for plant growth and sustainability in nutrient-poor, arid soils. Front. Plant Sci. 14:1147535. 10.3389/fpls.2023.1147535 37089637 PMC10117987

[B47] Khan Z. Midega C. A. Hooper A. Pickett J. (2016b). Push-pull: chemical ecology-based integrated pest management technology. J. Chem. Ecol. 42, 689–697. 10.1007/s10886-016-0730-y 27392788

[B48] Khan Z. R. Midega C. A. O. Pittchar J. O. Murage A. W. Birkett M. A. Bruce T. J. A.. (2014). Achieving food security for one million sub-Saharan African poor through push-pull innovation by 2020. Philosophical Transactions of the Royal Society B: Biological Sciences 369(1639). 10.1098/rstb.2012.0284 24535391 PMC3928888

[B49] Khan Z. R. Pickett J. A. (2004). The ‘push-pull'strategy for stemborer management: a case study in exploiting biodiversity and chemical ecology. Ecol. Eng. Pest Manage. Adv. Habitat Manip. Arthropods 22, 155–164. 10.1079/9780851999036.015536007395

[B50] Khan Z. R. Pickett J. A. Berg J. V. D. Wadhams L. J. Woodcock C. M. (2000). Exploiting chemical ecology and species diversity: stem borer and striga control for maize and sorghum in Africa. Pest Manage. Sci. Formerly Pesticide Sci. 56, 957–962. 10.1002/1526-4998(200011)56:11andlt;957::AID-PS236andgt;3.0.CO;2-T

[B51] Khan Z. R. Pickett J. A. Midega C. Pittchar J. (2016a). “Climate-smart push-pull-a conservation agriculture technology for food security and environmental sustainability in Africa,” in Conservation Agriculture for Africa: Building Resilient Farming Systems in a Changing Climate, 151–166. 10.1079/9781780645681.015134745184

[B52] Kitamura R. Maranho L. (2016). Phytoremediation of petroleum hydrocarbons-contaminated soil using *Desmodium incanum* DC., Fabaceae. Rev Latinoam Biotecnol Ambient Algal 7:1. 10.7603/s40682-016-0001-1

[B53] Krauss U. Rees R. Stirrup T. Argyle T. George A. Arroyo C.. (2013). Mycoparasitism by *Clonostachys byssicola* and *Clonostachys rosea* on *Trichoderma* spp. from cocoa (*Theobroma cacao*) and implication for the design of mixed biocontrol agents. Biol. Control 67, 317–327. 10.1016/j.biocontrol.2013.09.011

[B54] Lamprecht S. C. Knox-Davies P. S. Marasas W. F. O. (1988). Fungi associated with root rot of annual *Medicago* spp. in South Africa. Phytophylactica 20, 281–286.

[B55] Li J. Epa R. Scott N. E. Skoneczny D. Sharma M. Snow A. J. D.. (2020). A sulfoglycolytic entner-doudoroff pathway in *Rhizobium leguminosarum* bv. trifolii SRDI565. Appl. Environ. Microbiol. 86, 1–15. 10.1128/AEM.00750-20 32444469 PMC7376563

[B56] Liu A. Contador C. A. Fan K. Lam H. M. (2018). Interaction and regulation of carbon, nitrogen, and phosphorus metabolisms in root nodules of legumes. Front. Plant Sci. 9:1860. 10.3389/fpls.2018.01860 30619423 PMC6305480

[B57] Lozupone C. Knight R. (2005). UniFrac: a new phylogenetic method for comparing microbial communities. Appl. Environ. Microbiol. 71, 8228-8235. 10.1128/AEM.71.12.8228-8235.2005 16332807 PMC1317376

[B58] Lupini S. Nguyen H. N. Morales D. House G. L. Paudel S. Chain P. S. G.. (2023). Diversity of fungal microbiome obtained from plant rhizoplanes. Sci. Total Environ. 892:164506. 10.1016/j.scitotenv.2023.164506 37295515

[B59] Ma X. Zheng C. Hu C. Rahman K. Qin L. (2011). The genus *Desmodium* (Fabaceae)-traditional uses in Chinese medicine, phytochemistry and pharmacology. J. Ethnopharmacol. 138, 314–332. 10.1016/j.jep.2011.09.053 22004895

[B60] Makoudi B. Kabbadj A. Mouradi M. Amenc L. Domergue O. Blair M.. (2018). Phosphorus deficiency increases nodule phytase activity of faba bean–rhizobia symbiosis. Acta Physiol. Plantarum 40, 1–10. 10.1007/s11738-018-2619-6

[B61] Mayhood P. Mirza B. S. (2021). Soybean root nodule and rhizosphere microbiome: distribution of rhizobial and non-rhizobial endophytes. Appl. Environ. Microbiol. 87:20. 10.1128/AEM.02884-20 33674438 PMC8117765

[B62] McMurdie P. J. Holmes S. (2013). Phyloseq: An R package for reproducible interactive analysis and graphics of microbiome census data. PLoS ONE 8:e61217. 10.1371/journal.pone.0061217 23630581 PMC3632530

[B63] Mikryukov V. (2020). metaMisc: Miscellaneous Functions for Metagenomic Analysis. R-Package (v.0.04).

[B64] Moparthi S. Burrows M. Mgbechi-ezeri J. Agindotan B. (2021). *Fusarium* spp. associated with root rot of pulse crops and their cross-pathogenicity to cereal crops in Montana. Plant Dis. 105, 548–557. 10.1094/PDIS-04-20-0800-RE 32870113

[B65] Muazzam K. A. A. R. Darah I. (2020). The evaluation of antibacterial activity of fungal endophyte *Ceratobasidium ramicola* IBRLCM127 colonizing in rhizomes of medicinal plant, *Curcuma mangga Valeton* and Zijp. IOP Conf. Series: Earth Environ. Sci. 596:2083. 10.1088/1755-1315/596/1/012083

[B66] Muresu R. Maddau G. Delogu G. Cappuccinelli P. Squartini A. (2010). Bacteria colonizing root nodules of wild legumes exhibit virulence-associated properties of mammalian pathogens. Int. J. Gen. Mol. Microbiol. 97, 143–153. 10.1007/s10482-009-9396-6 19916054

[B67] Muthini M. Awino R. Kirui K. C. Koech K. Jalloh A. A. Njeru E. M.. (2020). Optimizing *Rhizobium*-legume symbiosis in smallholder agroecosystems. Sust. Agric. Rev. 45: Legume Agric. Biotechnol. 1, 159–177. 10.1007/978-3-030-53017-4_8

[B68] Mutyambai D. M. Bass E. Luttermoser T. Poveda K. Midega C. A. Khan Z. R.. (2019). More than “push” and “pull”? plant-soil feedbacks of maize companion cropping increase chemical plant defenses against herbivores. Front. Ecol. Evol. 7:217. 10.3389/fevo.2019.00217

[B69] Mutyambai D. M. Bruce T. J. A. Van den Berg J. Midega C. A. O. Pickett J. A. Khan Z. R.. (2016). An indirect defense trait mediated through egg-induced maize volatiles from neighbouring plants. PLoS ONE 11, 1–15. 10.1371/journal.pone.0158744 27392034 PMC4938388

[B70] Mutyambai D. M. Mutua J. M. Kessler A. Jalloh A. A. Njiru B. N. Chidawanyika F.. (2023). Push-pull cropping system soil legacy alter maize metabolism and fall armyworm, *Spodoptera frugiperda* (Lepidoptera: Noctuidae) resistance through tritrophic interactions. Plant Soil 12, 1–13. 10.21203/rs.3.rs-3223509/v1

[B71] Ndayisaba P. C. Kuyah S. Midega C. A. O. Mwangi P. N. Khan Z. R. (2022). Push-pull technology improves carbon stocks in rainfed smallholder agriculture in Western Kenya. Carbon Manage. 13, 127–141. 10.1080/17583004.2022.2035823

[B72] Ndayisaba P. C. Kuyah S. Midega C. A. O. Mwangi P. N. Khan Z. R. (2023). Push-pull technology enhances resilience to climate change and prevents land degradation: perceptions of adopters in western Kenya. Farming Syst. 1:100020. 10.1016/j.farsys.2023.100020

[B73] Nilsson R. H. Larsson K. H. Taylor A. F. S. Bengtsson-Palme J. Jeppesen T. S. Schigel D.. (2019). The UNITE database for molecular identification of fungi: Handling dark taxa and parallel taxonomic classifications. Nucleic Acids Res. 47, D259–D264. 10.1093/nar/gky1022 30371820 PMC6324048

[B74] Nyberg Y. Jonsson M. Ambjörnsson E. L. Wetterlind J. Öborn I. (2020). Smallholders' awareness of adaptation and coping measures to deal with rainfall variability in Western Kenya. Agroecol. Sust. Food Syst.ms 44, 1280–1308. 10.1080/21683565.2020.1782305

[B75] Nygren K. Dubey M. Zapparata A. Iqbal M. Tzelepis G. D. Brandström M.. (2018). The mycoparasitic fungus *Clonostachys rosea* responds with both common and specific gene expression during interspecific interactions with fungal prey. Evol. Appl. 11, 931–949. 10.1111/eva.12609 29928301 PMC5999205

[B76] Odendo M. Obare G. A. Salasya B. (2010). Determinants of the speed of adoption of soil fertility-enhancing technologies in Western Kenya (No,. 308-2016-5071). 10.22004/ag.econ.96192

[B77] OECD/FAO (2016). Agriculture in sub-Saharan Africa: prospects and challenges for the next decade. OECD-FAO Agric. Outlook 22, 59–95. 10.1787/agr_outlook-2016-5-en

[B78] Ogenga J. O. (2021). Analysis of seasonal rainfall variability in rainfed agriculture in Homa Bay County. Int. J. Sci. Res. Pub. 11:11132. 10.29322/IJSRP.11.03.2021.p11132

[B79] Ojiem J. O. Vanlauwe B. De Ridder D. Giller N. (2007). Niche-based assessment of contributions of legumes to the nitrogen economy of Western Kenya smallholder farms. Plant Soil 292, 119–135. 10.1007/s11104-007-9207-7

[B80] Oksanen J. Blanchet F. G. Kindt R. Legendre P. Minchin P. R. O'Hara R. B.. (2012). Vegan: Community Ecology Package. Software. Available online at: http://CRAN.R-project.org/package=vegan

[B81] Okumoto S. Pilot G. (2011). Amino acid export in plants: a missing link in nitrogen cycling. Mol. Plant, 4, 453–463. 10.1093/mp/ssr003 21324969 PMC3143828

[B82] Oldroyd G. E. D. (2013). Speak, friend, and enter: signalling systems that promote beneficial symbiotic associations in plants. Nat. Rev. Microbiol. 11, 252–263. 10.1038/nrmicro2990 23493145

[B83] Ormeño-Orrillo E. Martínez-Romero E. (2019). A genomotaxonomy view of the *Bradyrhizobium* genus. Front. Microbiol. 10:1334. 10.3389/fmicb.2019.01334 31263459 PMC6585233

[B84] Pang J. Palmer M. Sun H. J. Seymour C. O. Zhang L. Hedlund B. P.. (2021). Diversity of root nodule-associated bacteria of diverse legumes along an elevation gradient in the Kunlun Mountains, China. Front. Microbiol. 12:633141. 10.3389/fmicb.2021.633141 33664721 PMC7920992

[B85] Parker M. A. (2002). *Bradyrhizobia* from wild *Phaseolus, Desmodium*, and *Macroptilium* species in Northern Mexico. Appl. Environ. Microbiol. 68, 2044–2048. 10.1128/AEM.68.4.2044-2048.2002 11916730 PMC123864

[B86] Parks D. H. Tyson G. W. Hugenholtz P. Beiko R. G. (2014). STAMP: Statistical analysis of taxonomic and functional profles. Bioinformatics 30, 3123–3124. 10.1093/bioinformatics/btu494 25061070 PMC4609014

[B87] Peter E. Tamiru A. Sevgan S. Dubois T. Kelemu S. Kruger K.. (2023). Companion crops alter olfactory responses of the fall armyworm (*Spodoptera frugiperda*) and its larval endoparasitoid (*Cotesia icipe*). *Chem. Biol. Technol. Agric*. 10:61. 10.1186/s40538-023-00415-6

[B88] Quast C. Pruesse E. Yilmaz P. Gerken J. Schweer T. Yarza P.. (2013). The SILVA ribosomal RNA gene database project: improved data processing and web-based tools. Nucleic Acids Res. 41, D590–D596. 10.1093/nar/gks1219 23193283 PMC3531112

[B89] Rajendran G. Sing F. Desai A. J. Archana G. (2008). Enhanced growth and nodulation of pigeon pea by co-inoculation of *Bacillus* strains with *Rhizobium* spp. Bioresour. Technol. 99, 4544–4550. 10.1016/j.biortech.2007.06.057 17826983

[B90] Russell A. J. Bidartondo M. I. Butterfield B. G. (2002). The root nodules of the Podocarpaceae harbour arbuscular mycorrhizal fungi. New Phytol. 156, 283–295. 10.1046/j.1469-8137.2002.00504.x 33873271

[B91] Sahu S. Prakash A. Shende K. (2019). *Talaromyces trachyspermus*, an endophyte from *Withania somnifera* with plant growth promoting attributes. Environ. Sust. 2, 13–21. 10.1007/s42398-019-00045-5

[B92] Sang M. K. Jeong J. J. Kim J. Kim K. D. (2018). Growth promotion and root colonisation in pepper plants by phosphate-solubilising *Chryseobacterium* sp. strain ISE14 that suppresses *Phytophthora* blight. Annal. Appl. Biol. 172, 208–223. 10.1111/aab.12413

[B93] Schloss P. D. (2020). Removal of rare amplicon sequence variants from 16S rRNA gene sequence surveys biases the interpretation of community structure data. BioRxiv 11, 1–29. 10.1101/2020.12.11.422279

[B94] Sharaf H. Rodrigues R. R. Moon J. Zhang B. Mills K. Williams M. A.. (2019). Unprecedented bacterial community richness in soybean nodules vary with cultivar and water status. Microbiome 7, 1–18. 10.1186/s40168-019-0676-8 30992078 PMC6469096

[B95] Shi S. Richardson A. E. O'Callaghan M. DeAngelis K. M. Jones E. E. Stewart A.. (2011). Effects of selected root exudate components on soil bacterial communities. FEMS Microbiol. Ecol. 77, 600–610. 10.1111/j.1574-6941.2011.01150.x 21658090

[B96] Slabbert M. S. Kühn-institut J. Kühn-institut J. (2023). Preliminary study on seasonal diversity of root endophytic fungi and bacteria associated with Sainfoin (*Onobrychis viciifolia*) in South Africa. 10.21203/rs.3.rs-3128123/v1

[B97] Strodtman K. N. Frank S. Stevenson S. Thelen J. J. Emerich D. W. (2018). Proteomic characterization of *Bradyrhizobium diazoefficiens* bacteroids reveals a post-symbiotic, hemibiotrophic-like lifestyle of the bacteria within senescing soybean nodules. Int. J. Mol. Sci. 19:3947. 10.3390/ijms19123947 30544819 PMC6320959

[B98] Strom N. Hu W. Haarith D. Chen S. Bushley K. (2020). Corn and soybean host root endophytic fungi with toxicity toward the soybean cyst nematode. Phytopathology 110, 603–614. 10.1094/PHYTO-07-19-0243-R 31631807

[B99] Sudtachat N. Ito N. Itakura M. Masuda S. Eda S. Mitsui H.. (2009). Aerobic vanillate degradation and C1 compound metabolism in *Bradyrhizobium japonicum*. Appl. Environ. Microbiol. 75, 5012–5017. 10.1128/AEM.00755-09 19502448 PMC2725485

[B100] Tadele Z. (2017). Raising crop productivity in Africa through intensification. Agronomy 7:22. 10.3390/agronomy7010022

[B101] Tiwari S. Prasad V. Chauhan P. S. Lata C. (2017). *Bacillus amyloliquefaciens* confers tolerance to various abiotic stresses and modulates plant response to phytohormones through osmoprotection and gene expression regulation in rice. Front. Plant Sci. 8:1510. 10.3389/fpls.2017.01510 28900441 PMC5581838

[B102] Toniutti M. A. Fornasero L. V. Albicoro F. J. Martini M. C. Draghi W. Alvarez F.. (2017). Nitrogen-fixing rhizobial strains isolated from *Desmodium incanum* DC in Argentina: phylogeny, biodiversity and symbiotic ability. Syst. Appl. Microbiol. 40, 297–307. 10.1016/j.syapm.2017.04.004 28648724

[B103] Tsanuo M. K. Hassanali A. Hooper A. M. Khan Z. Kaberia F. Pickett J. A.. (2003). Isoflavanones from the allelopathic aqueous root exudate of *Desmodium uncinatum*. Phytochemistry 64, 265–273. 10.1016/S0031-9422(03)00324-8 12946425

[B104] Vincent J. (1970). A Manual for the Practical Study of Root Nodule Bacteria. Oxford: Blackwell Scientific Publications.

[B105] Wang N. Zhang S. Li Y. J. Song Y. Q. Lei C. Y. Peng Y. Y.. (2023). Novel isolate of *Cladosporium subuliforme* and its potential to control Asian citrus psyllid, *Diaphorina citri* Kuwayama (Hemiptera: Liviidae). *Egyptian J. Biol. Pest Control* 33:685. 10.1186/s41938-023-00685-0

[B106] Warnes G. R. Bolker B. Bonebakker L. Gentleman R. Huber W. Andy L.. (2016). Various R Programming Tools for Plotting Data. Available online at: https://cran.r-project.org/web/packages/gplots (accessed February 12, 2024).

[B107] Wekesa C. Jalloh A. A. Muoma J. O. Korir H. Omenge K. M. Maingi J. M.. (2022). Distribution, characterization and the commercialization of elite *Rhizobia* strains in Africa. Int. J. Mol. Sci. 23:6599. 10.3390/ijms23126599 35743041 PMC9223902

[B108] Were E. Schöne J. Viljoen A. Rasche F. (2022). Phenolics mediate suppression of *Fusarium oxysporum* f. sp. cubense TR4 by legume root exudates. Rhizosphere 21:100459. 10.1016/j.rhisph.2021.100459

[B109] Wickham H. (2016). ggplot2: Elegant Graphics for Data Analysis. New York, NY: Springer-Verlag.

[B110] Wickham H. Averick M. Bryan J. Chang W. McGowan L. François R.. (2019). Welcome to the Tidyverse. J. Open Source Software 4:1686. 10.21105/joss.01686

[B111] Wingett S. W. Andrews S. (2018). FastQ Screen: a tool for multi-genome mapping and quality control. F1000Research 7:1338. 10.12688/f1000research.15931.1 30254741 PMC6124377

[B112] Xu K. W. Zou L. Penttinen P. Zeng X. Liu M. Zhao K.. (2016). Diversity and phylogeny of rhizobia associated with *Desmodium* spp. in Panxi, Sichuan, China. Syst. Appl. Microbiol. 39, 33–40. 10.1016/j.syapm.2015.10.005 26654528

[B113] Xu L. Zhang Y. Wang L. Chen W. Wei G. (2014). Diversity of endophytic bacteria associated with nodules of two indigenous legumes at different altitudes of the Qilian Mountains in China. Syst. Appl. Microbiol. 37, 457–465. 10.1016/j.syapm.2014.05.009 24985194

[B114] Xu S. Li Z. Tang W. Dai Z. Zhou L. Feng T.. (2022). MicrobiotaProcess: A Comprehensive R Package for Managing and Analyzing Microbiome and Other Ecological Data Within the Tidy Framework. 10.21203/rs.3.rs-1284357/v136895758

[B115] Ye Y. Zhan X. Wang K. Zhong J. Liao F. Chen W.. (2023). A symbiotic fungus *Sistotrema* benefits blueberry rejuvenation and abiotic stress tolerance. J. Fungi 9:779. 10.3390/jof9070779 37504767 PMC10381331

[B116] Yuan Z. L. Chen Y. C. Ma X. J. (2011). All aspects of plant biology symbiotic fungi in roots of *Artemisia annua* with special reference to endophytic colonizers. Plant Biosyst. 145, 495–502. 10.1080/11263504.2010.544863

[B117] Zuo Y. L. Hu Q. N. Qin L. Liu J. Q. He X. L. (2022). Species identity and combinations differ in their overall benefits to *Astragalus adsurgens* plants inoculated with single or multiple endophytic fungi under drought conditions. Front. Plant Sci. 13:933738. 10.3389/fpls.2022.933738 36160950 PMC9490189

