# Supplementary material for: Comparative microbiome diversity in root-nodules of three Desmodium species used in push-pull cropping system
Source: Front Microbiol. 2024 Jun 20;15:1395811. doi: 10.3389/fmicb.2024.1395811 (PMC11222577; doi:10.3389/fmicb.2024.1395811)
Supplement: Supplementary file 2 [file Table_2.DOCX]

**##Setting Working Directory for ITS (Fungi)**

setwd("C:/Users/user/Desktop/RESULTS/Study group 2")

getwd()

**####Load required packages**

library (DECIPHER)

library (tidyverse)

library (ALDEx2)

library (ANCOMBC)

library(ape)

library (Cairo)

library(coin)

library(dada2)

library(data.table)

library(dendextend)

library(devtools)

library(dplyr)

library(eulerr)

library(extrafont)

library(ggplot2)

library(ggpubr)

library(ggrepel)

library(ggtree)

library(janitor)

library(microbiome)

library(microbiomeutilities)

library (NADA)

library(phyloseq)

library(readr)

library(readxl)

library(reshape)

library(reshape2)

library(vegan)

library (VennDiagram)

library(viridis)

library(viridisLite)

library(csv)

library(tidyr)

library(ggnewscale)

library(metagMisc)

library (MicrobiotaProcess)

library(patchwork)

library(ggtreeExtra)

**#####****Pre-processing of the raw reads**

list.files()

path<-list.files(path =" C:/Users/user/Desktop/RESULTS/Study group 2/data/nodules_ITS",

pattern = "fastq.gz",

full.names = "TRUE") #read in the data

path #view the data

**#####Filter and trim (Sort ensures forward/reverse reads are in same order)**

fnFs <- sort(list.files("C:/Users/user/Desktop/RESULTS/Study group 2/data /nodules_ITS ", pattern="_1_1.fastq.gz")) #read all the foward reads

fnRs <- sort(list.files("C:/Users/user/Desktop/RESULTS/Study group 2/data /nodules_ITS ", pattern="_1_2.fastq.gz")) #read all the reverse reads

**#### Extract sample names; filenames have format: SAMPLENAME_XXX.fastq**

fnFs

fnRs

**###split file name**

sample.names <- sapply(strsplit(fnFs, "_"), `[`, 1)

sample.names

# Specify the full path to the fnFs and fnRs

fnFs <- file.path("C:/Users/user/Desktop/RESULTS/Study group 2/data/nodules_ITS ", fnFs)

fnRs <- file.path("C:/Users/user/Desktop/RESULTS/Study group 2/data/nodules_ITS ", fnRs)

fnFs

fnRs

plotQualityProfile(fnFs[1:2])

plotQualityProfile(fnRs[1:2])

filt_path <- file.path("C:/Users/user/Desktop/RESULTS/Study group 2/data /nodules_ITS , "filtered") # Place filtered files in filtered/ subdirectory

filtFs <- file.path(filt_path, paste0(sample.names, "_F_filt.fastq.gz"))

filtRs <- file.path(filt_path, paste0(sample.names, "_R_filt.fastq.gz"))

filtRs

out <- filterAndTrim(fnFs, filtFs, fnRs, filtRs, truncLen=c(250,230),

maxN=0, maxEE=c(2,5), truncQ=2, rm.phix=TRUE,

compress=TRUE)

**###Filter the forward and reverse reads, truncate anythything with 240 in f and 160 r drop it and maximum number of sequences with n, drop and maximum error is 2,2**

head(out)

tail(out)

**####Learn error rates, and time the procedure**

system.time(errF <- learnErrors(filtFs, multithread=TRUE))

# Learn error rates, time the procedure

system.time(errR <- learnErrors(filtRs, multithread=TRUE))

plotErrors(errF, nominalQ=TRUE)

derepFs <- derepFastq(filtFs, verbose=TRUE)

derepRs <- derepFastq(filtRs, verbose=TRUE)

**###sample inferencing**

system.time(dadaFs <- dada(derepFs, err=errF, multithread=TRUE))

system.time(dadaRs <- dada(derepRs, err=errF, multithread=TRUE))

**##Inspecting the dada-class object returned by dada2**

dadaFs[[1]]

dadaRs[[1]]

dadaFs[[14]]

dadaRs[[14]]

**###Merge both the forward and reverse reads**

mergers <- mergePairs(dadaFs, derepFs, dadaRs, derepRs, verbose=TRUE)

head(mergers[[1]])

seqtab <- makeSequenceTable(mergers)

**#####The sequences being tabled vary in length.**

dim(seqtab)

table(nchar(getSequences(seqtab)))

**#####Inspect distribution of sequence lengths**

dim(seqtab)

hist(nchar(getSequences(seqtab)), main="Distribution of sequence lengths")

seqtab.nochim <- removeBimeraDenovo(seqtab, method="consensus", multithread=TRUE, verbose=TRUE**)**

**#####Remove chimeras/bimeras**

dim(seqtab.nochim)

sum(seqtab.nochim)/sum(seqtab)

getN <- function(x) sum(getUniques(x))

track <- cbind(out, sapply(dadaFs, getN), sapply(mergers, getN), rowSums(seqtab), rowSums(seqtab.nochim))

colnames(track) <- c("input", "filtered", "denoised", "merged", "tabled", "nonchim")

rownames(track) <- sample.names

head(track)

tail(track)

**#####Assigning taxonomy against the UNITE ref2 database**

taxtrain <-"C:/Users/user/Desktop/RESULTS/Study group 2/REF/ref2.FASTA"

taxa <- assignTaxonomy(seqtab.nochim, taxtrain, multithread=TRUE)

unname(head(taxa))

unname(tail(taxa))

taxa.print <- taxa

**#####Removing sequence row names for display only**

rownames(taxa.print) <- NULL

head(taxa.print)

tail(taxa.print)

taxa.print

sequences<-getSequences(seqtab.nochim)

names(sequences)<-sequences

alignment <- AlignSeqs(DNAStringSet(sequences), anchor=NA)

asv_headers <- vector(dim(seqtab.nochim)[2], mode="character")

for (i in 1:dim(seqtab.nochim)[2]) {

asv_headers[i] <- paste(">ASV", i, sep="_")

head(asv_headers)

asv_headers

#library(tidyverse)

#Generating sequence table

seqs <- getSequences(seqtab.nochim)

asv_fastaITS <- c(rbind(asv_headers, seqs))

head(asv_fastaITS)

tail(asv_fastaITS)

**####Writing the fasta file format of the sequences**

write(asv_fastaITS, "C:/Users/user/Desktop/RESULTS/Study group 2/Data_output.csv")

**###Creating a sequence table data frame**

names(seqs) <- sub(">", "", asv_headers)

seqs <- as.data.frame(seqs)

seqs <- seqs %>% rownames_to_column(var = "OTU")

**####Generating a feature table with newly defined row names**

count_asv_tab <- t(seqtab.nochim)

row.names(count_asv_tab) <- sub(">", "", asv_headers)

write.table(count_asv_tab, "C:/Users/user/Desktop/RESULTS/Study group 2/ASVs_counts.csv", sep=",", quote=F, col.names=NA)

**####Generating a taxonomy table with the newly defined row names**

rownames(taxa) <- gsub(pattern=">", replacement="", x=asv_headers)

head(taxa)

taxa

write.csv (taxa, file="C:/Users/user/Desktop/RESULTS/Study group 2/ASVs_taxonomy.csv")

**###############Loading the data**

**##### Import the tax table**

taxa<-read.csv ("C:/Users/user/Desktop/RESULTS/Study group 2/ASVs_taxonomy.csv", sep = ",", header = TRUE, row.names = 1)

head(taxa)

names(taxa)

names(taxa)<-c("OTU","X","Kingdom","Phylum","Class","Order","Family","Genus","Species")

dim(taxa)

taxa<-t(taxa)

taxa<-as.matrix(taxa) #converting tax dataframe to a matrix

#head(tax)

taxa<-(t(taxa))

taxa<-phyloseq::tax_table(taxa) #converting the taxa matrix to phyloseq object

#class(tax)

#head(tax)

**####Loading ASVs counts table (OTU table)**

OTU<-read.csv("C:/Users/user/Desktop/RESULTS/Study group 2/nodules.ASVs_counts.csv",

sep = ",",

header = TRUE,

row.names = 1)

head(OTU)

names(OTU)

names(OTU)<-c("10d","11d","12d","13d","14d","15d","16d","17d",

"18d","19d","1d","20d","21d","22d","23d","24d", "2d","3d","4d",

"5d","6d","7d","8d","9d")

dim(OTU)

OTU<-otu_table(OTU,taxa_are_rows = TRUE) #converting otu matrix to phyloseq object.

head(OTU)

**####Importing the sample metadata**

meta<-read.csv("C:/Users/user/Desktop/RESULTS/Study group 2/metadata.csv",

sep= ",",

header=TRUE,

row.names = 1)

head(meta)

names(meta)

meta<-sample_data(meta) #converting the metadata to a phyloseq object

**####merging the preceeding 3 objects.**

ps1<-phyloseq(taxa,OTU)

ps1

ps2 <-merge_phyloseq(ps1,meta)

ps2

**####filtering the unwanted/undesired sequences**

ps3<-subset_taxa(ps3, !is.na(Genus) & !Genus %in% c("", "NA")) ####eliminating N/As in the genus column

ntaxa(ps3)

**########Pruning (filtering out taxa having low total abundance across all samples)**

ps3

ps4 <- prune_taxa(taxa_sums(ps3) > 24, ps3)

ps4

library(metagMisc)

**####Extracting the filtered taxonomy and feature tables for barplot plotting**

sample_names(ps4)<-paste0("z_", sample_names(ps4))

tax_table <- phyloseq_to_df(ps4, addtax = T, addtot = F, addmaxrank = F)

cumulation <- tax_table %>% adorn_totals(c("col"))

cumulation <- cumulation [order (cumulation$Total, decreasing = TRUE),]

**####merging the blast taxonomic classification to blast abundance table**

merged_data <- tax_table

write.csv(merged_data, file="C:/Users/user/Desktop/RESULTS/Study group 2/ITS_Taxonomic_SpeciesClassification_Abudance.csv")

**#####create a new data frame (Featured_table) for species**

Featured_table <- merged_data[, c(8,9:32)] #change 7 to 8 to go to species

dim(merged_data)

merged_data$Gspecies<-merged_column <- paste (merged_data$Genus, merged_data$Species, sep = " ")

**#####creating a new column in the data frame called Gspecies**

names(merged_data)

dim(merged_data)

Featured_table <- merged_data[,c(33,9:32)]

group <- Featured_table %>%

group_by(Gspecies)%>%

summarise_if(is.numeric, sum)

**#######Grouping the data based on the Gspecies column**

group$Gspecies <- gsub("\\bNA\\b", "spp", group$Gspecies)

**######Replacing all N/As in the Gspecies column with spp.**

group$Gspecies

names(group)

dim(group)

names(group)

dim(group)

**#####Defining the treatments**

GLD<-group[,c(1,2,5,7,11,12, 19,24,25)]

SLD<-group[,c(1,3,6,9,13,16,17,20,21)]

AID<-group[,c(1,4,8,10,14,15,18,22,23)]

dim(GLD)

GLD_total <- GLD %>% adorn_totals(c("col"))

GLD_total <- mutate(GLD_total, GLD=rowSums(GLD_total[10])/9)

GLD_total <- GLD_total[,c(1,10)]

dim(SLD)

SLD_total <- SLD %>% adorn_totals(c("col"))

SLD_total <- mutate(SLD_total, SLD=rowSums(SLD_total[10])/9)

SLD_total <- SLD_total[,c(1,10)]

dim(AID)

AID_total <- AID %>% adorn_totals(c("col"))

AID_total <- mutate(AID_total, AID=rowSums(AID_total[10])/9)

AID_total <- AID_total[,c(1,10)]

**####Merging the totals for each group based on Gspecies**

merged <- Reduce(function(x,y) merge(x,y,by="Gspecies",all=TRUE),

list(GLD_total,SLD_total, AID_total))

names(merged)<-c('Gspecies','GLD','SLD','AID')

dim(merged)

**####Calculating the total abundance per genus and ordering from the most abundant to the lowest**

cumulation <- merged %>% adorn_totals(c("col"))

cumulation <- cumulation [order(cumulation$Total, decreasing = TRUE),]

cumulation$perc = cumulation$Total / sum(cumulation$Total) * 100

tired<-head(cumulation$Gspecies, n=30) ###this will only consider the

li2 <- append(tired,"Others")

print(li2)

genus_Rep <- li2

#### aggregating the data based on the Gspecies column

group <- aggregate(merged[-1], list(Gspecies = replace(merged$ Gspecies,!(merged$ Gspecies %in% genus_Rep), "Others")), sum)

dim(group)

All<- group[,c(1:4)]

**####Preparing the data for visualization**

bar_all <- bar_all %>%

gather(value = "abundance", key = "Infection", -Gspecies)

bar_all <- as.data.frame(gsub("\\(", " (", as.matrix(bar_all)))

**####Viewing sample diversity**

**####Converting the abundances into percentage**

bar_all <- adorn_percentages(All, denominator = "col", na.rm = T)

bar_all %>%

adorn_totals("row") %>%

adorn_pct_formatting()

dist_all<-bar_all %>%

adorn_totals("row") %>%

adorn_pct_formatting()

dist_all

write.csv(dist_all, "C:/Users/user/Desktop/RESULTS/Study group 2/ITS_Desmodium_root_nodule_Species_Percentage.csv")

**####Gathering the data**

bar_all <- bar_all %>%

gather(value = "abundance", key = "Infection", -Gspecies)

bar_all <- as.data.frame(gsub("\\(", " (", as.matrix(bar_all)))

**####coerce the dataframe columns into respective data type**

bar_all$Gspecies <- as.factor(bar_all$Gspecies)

bar_all$Infection<- as.character(bar_all$Infection)

bar_all$abundance <- as.numeric(bar_all$abundance)

**####Ordering the data for plotting**

bar_all$Gspecies <- reorder(bar_all$Gspecies, bar_all$abundance)

bar_all$Gspecies <- factor(bar_all$Gspecies, levels=rev(levels(bar_all$Gspecies)))

bar_all$Gspecies<- factor(bar_all$Gspecies,

levels=genus_Rep)

bar_all

**####Defining the color pallete**

myPalette <- c("#1B9E77", "#D95F02", "#7570B3", "#E7298A", "#99000D", "#E6AB02", "#A6761D", "#666666","#FDCDAC", "#1F78B4", "#B2DF8A", "#33A02C", "#CBD5E8", "#E31A1C", "#FDBF6F", "#FF7F00","#4A1486","#C0C0C0","#B3E2CD","#FFFF33", "#5172b2","#F4CAE4", "#E6F5C9", "#FCFBFD","#139BF1","#09FF00","#065535", "#1D91C0", "#C0FFEE","#B35806","#0C2C84","#D0ED0E","#092617","#499976","#4D5D53","#E48400","#6082B6","#316689","#CEFB02","#738678","#645452","#EEA47FFF", "#00539CFF", "#FC766AFF", "#42EADDFF", "#00A4CCFF", "#69B3BB", "#B589D6","#D1DFB7","#97BC62FF","#D198C5FF","#000000", "#CBCE91FF", "#616247FF", "#D64161FF","#435E55FF", "#DD4132FF","#CE4A7EFF", "#BD7F37FF","#FFA351FF","#185E57", "#FCE3E3", "#EF6C6C", "#EF6C9A", "#93103E", "#F7E3FC", "#D56CEF", "#BD19E6", "#8D6CEF", "#DBD1FA", "#BFC8F8", "#1531BC", "#9FA5F3", "#95C7F3", "#6CEFC8", "#6CEF84", "#91E619", "#B7CEEC", "#9AFEFF", "#57FEFF", "#78C7C7", "#46C7C7", "#00A36C", "#728C00", "#4E9268", "#6CC417", "#64E986", "#F5E216", "#FFCE44", "#8B8000", "#660000", "#610541", "#E56E94", "#F660AB", "#E3319D", "#FF77FF", "#C45AEC", "#6960EC", "#736AFF", "#F9B7FF", "#FCDFFF", "#D291BC", "#614051", "#FEA3AA", "#7D0541")

**###### Defining the group names**

guide_italics <- guides(fill = guide_legend(label.theme = element_text(size = 15, face = "italic", colour = "Black", angle = 0)))

**######Plotting the barplot**

p_all <- ggplot(bar_all,aes(x = fct_inorder(Infection), y = abundance), labs(fill= Gspecies), group=row.names(bar_all))+ xlab("Cropping system")+ ylab("%abundance") + geom_col(aes(fill = Gspecies),position = position_stack(reverse = FALSE))+

theme(axis.text.x = element_text(angle = 72,size = 15, hjust = 1, face = "italic", family = "Arial"))+

scale_fill_manual(values = myPalette)+

#guides(fill = guide_legend(reverse = FALSE))+

guide_italics+

theme(legend.text = element_text(size = 8, colour = "black", face = "italic", family = "Arial"), legend.text.align = 0)+

theme(axis.text.y = element_text(angle = 0, vjust = 0.5, size = 8, family = "Arial"))+

theme(axis.text = element_text(colour = "black", size = 8, family = "Arial"))+

theme(axis.line = element_line())+

theme(panel.background = element_rect(fill = "white"),plot.margin = margin(0.1, 0.1, 0.1, 0.1, "cm"), plot.background = element_rect(colour = NULL, size = 1))+

theme(axis.ticks.length.y = unit(.15, "cm"), axis.ticks.length.x = unit(.25, "cm"), axis.text.x = element_text(margin = margin(t = .3, unit = "cm")))+

theme(legend.position = "right", legend.justification = "top", legend.direction = "vertical", legend.text = element_text(size = 10))+

theme(legend.key = element_rect(fill = "white"))+

theme(legend.title = element_text(face = NULL, size = 8, family = "Arial"))+

theme(panel.background = element_blank(), axis.text = element_blank())+

theme(axis.text = element_text(colour = "black", size = 8, family = "Arial"))

###font_import

p_fmty_12s<-p_all + theme(text = element_text(size = 14))

p_fmty_12s

ggsave("C:/Users/user/Desktop/RESULTS/Study group 2/Top30_ITS_ Gspecies _Abundance.png",

width = 16, height = 12, dpi = 600)

ggsave("C:/Users/user/Desktop/RESULTS/Study group 2/Top30_ITS_ Gspecies _Abundance.svg",

width = 16, height = 12, dpi = 600)

ggsave("C:/Users/user/Desktop/RESULTS/Study group 2/Top30_ITS_ Gspecies _Abundance.tiff",

width = 16, height = 12, dpi = 600)

**####Alpha diversity**

ps4

#checking out the total read counts in the samples

reads <- sample_sums(ps4)

reads

summary(sample_sums(ps4))

**###Extracting the otu table from the phyloseq object and plotting the rarefaction curve**

otu_tab <- t(abundances(ps4))

p <- vegan::rarecurve(otu_tab,

step = 50, label = FALSE,

sample = min(rowSums(otu_tab),

col = "blue", cex = 0.6))

set.seed(9242)

**######Calculating an even sampling depth for all the samples**

rarefied <- rarefy_even_depth(ps4, sample.size = 24)

rarefied

**###Calculating the alpha diversity**

diversity <- alpha(rarefied, index = "all")

diversity <- rownames_to_column(diversity, "sample_id")

diversity

**###Extracting the sample metadata from the phyloseq object**

sdata1 <- meta(ps4)

sdata1 <- rownames_to_column(sdata1, "sample_id")

alphaobj <- get_alphaindex(rarefied)

head(as.data.frame(alphaobj))

as.data.frame(alphaobj)

tail(as.data.frame(alphaobj))

p_alpha_ty <- ggbox(alphaobj,indexNames=c("Observe","Chao1","ACE","Shannon"),

factorNames="Cropping_Type") +

scale_fill_manual(values=c("#D95F02","#1B9E77", "#0079FF"))+

theme(strip.background = element_rect(colour=NA, fill="grey"))

p_alpha_ty

ggsave("C:/Users/user/Desktop/RESULTS/Study group 2/ITS_Alpha_Diversity_PVALUES.jpeg",

width = 18, height = 12, dpi = 600)

ggsave("C:/Users/user/Desktop/RESULTS/Study group 2/ITS_Alpha_Diversity_PVALUES.png",

width = 18, height = 12, dpi = 600)

ggsave("C:/Users/user/Desktop/RESULTS/Study group 2/ITS_Alpha_Diversity_PVALUES.svg",

width = 18, height = 12, dpi = 600)

ggsave("C:/Users/user/Desktop/RESULTS/Study group 2/ITS_Alpha_Diversity_PVALUES.tiff",

width = 18, height = 12, dpi = 600)

**######Calculating alpha diversity based on sampling location**

alphaobj <- get_alphaindex(rarefied)

head(as.data.frame(alphaobj))

as.data.frame(alphaobj)

p_alpha_ty <- ggbox(alphaobj,indexNames=c("Observe","Chao1","ACE","Shannon"),

factorNames="Locations") +

scale_fill_manual(values=c("#D95F02","#1B9E77", "#0079FF", "#F4CE14"))+

theme(strip.background = element_rect(colour=NA, fill="grey"))

p_alpha_ty

ggsave("C:/Users/user/Desktop/RESULTS/Study group 2/ITS_Alpha_Diversity_PVALUES_location.jpeg",

width = 18, height = 12, dpi = 600)

ggsave("C:/Users/user/Desktop/RESULTS/Study group 2/ITS_Alpha_Diversity_PVALUES_location.png",

width = 18, height = 12, dpi = 600)

ggsave("C:/Users/user/Desktop/RESULTS/Study group 2/ITS_Alpha_Diversity_PVALUES_location.svg",

width = 18, height = 12, dpi = 600)

ggsave("C:/Users/user/Desktop/RESULTS/Study group 2/ITS_Alpha_Diversity_PVALUES_location.tiff",

width = 18, height = 12, dpi = 600)

**#####Visualizing Chao1 diversity estimates**

diversity

#Chao1 diversity estimates

chao1 <- diversity %>% dplyr::select(sample_id, Pielou)

chao_edited <- merge(chao1, sdata1, by = "sample_id", all = TRUE)

#chao_edited <- chao_edited[c(1:18, 62:86, 19:61),]

**###Confirming whether the chao1 indices are normally distributed**

shapiro.test(chao_edited$chao1)

#plotting chao1 distribution boxplot

Pchao <- ggboxplot(chao_edited, "Cropping_Type","chao1",

color = "Cropping_Type", palette =c("#F52100","#1B9E77", "#0079FF"),

add = "jitter", linetype = "solid", Family = "Palatino Linotype", add.params = list(),

error.plot = "pointrange", legand = NULL, size = NULL, width = 0.7, notch = FALSE, outlier.shape = 20, facet.by = NULL,

panel.labs = NULL, short.panel.labs = TRUE,bxp.errorbar = FALSE, bxp.errorbar.width = 0.4, ggtheme = theme_pubr())+

theme(axis.text.x = element_text(angle = 0, hjust = 0.5)) +

theme(legend.text = element_text(size = 10, colour = "black", face = "italic"), legend.text.align = 0)+

theme(axis.text.y = element_text(angle = 0, vjust = 0.5, size = 10))+

theme(axis.text = element_text(colour = "black", size = 10))+

theme(axis.line = element_line())+

theme(panel.background = element_rect(fill = "white"),plot.margin = margin(0.5, 0.5, 0.5, 0.5, "cm"), plot.background = element_rect(colour = NULL, size = 1))+

theme(axis.ticks.length.y = unit(.25, "cm"), axis.ticks.length.x = unit(.25, "cm"), axis.text.x = element_text(margin = margin(t = .3, unit = "cm")))+

theme(legend.justification = "top")+

theme(legend.position = "right")+

theme(legend.key = element_rect(fill = "white"))+

theme(legend.title = element_text(face = NULL, size = 10))+theme(panel.background = element_blank(), axis.text = element_blank())+

theme(axis.text = element_text(colour = "black", size = 10)+

theme(axis.line = element_line())+

theme(panel.background = element_rect(fill = "white"),plot.margin = margin(0.5, 0.5, 0.5, 0.5, "cm"), plot.background = element_rect(colour = "grey"))+

theme(axis.ticks.length.y = unit(.25, "cm"), axis.ticks.length.x = unit(.25, "cm"), axis.text.x = element_text(margin = margin(t = .3, unit = "cm")))+

theme(axis.title.y = element_text(size = 10, face = "plain", angle = 90))+

theme(axis.title.x = element_text(size = 10, angle = 0))) #+stat_compare_means()

Pchao<-Pchao + aes(x = fct_inorder(Cropping_Type)) + theme(legend.position = "none") + xlab("Cropping Type & Sample Type") + ylab("Chao1")

Pchao

**####Chao1 diversity estimates based on sampling location**

chao1 <- diversity %>% select(sample_id, chao1)

chao_edited <- merge(chao1, sdata1, by = "sample_id", all = TRUE)

#chao_edited <- chao_edited[c(1:18, 62:86, 19:61),]

**###confirming whether the chao1 indices are normally distributed**

shapiro.test(chao_edited$chao1)

**###Plotting chao1 distribution boxplot**

Pchao_l <- ggboxplot(chao_edited, "Locations","chao1",

color = "Locations", palette =c("#F52100","#1B9E77", "#0079FF", "#F4CE14"),

add = "jitter", linetype = "solid", Family = "Palatino Linotype", add.params = list(),

error.plot = "pointrange", legand = NULL, size = NULL, width = 0.7, notch = FALSE, outlier.shape = 20, facet.by = NULL,

panel.labs = NULL, short.panel.labs = TRUE,bxp.errorbar = FALSE, bxp.errorbar.width = 0.4, ggtheme = theme_pubr())+

theme(axis.text.x = element_text(angle = 0, hjust = 0.5)) +

theme(legend.text = element_text(size = 10, colour = "black", face = "italic"), legend.text.align = 0)+

theme(axis.text.y = element_text(angle = 0, vjust = 0.5, size = 10))+

theme(axis.text = element_text(colour = "black", size = 10))+

theme(axis.line = element_line())+

theme(panel.background = element_rect(fill = "white"),plot.margin = margin(0.5, 0.5, 0.5, 0.5, "cm"), plot.background = element_rect(colour = NULL, size = 1))+

theme(axis.ticks.length.y = unit(.25, "cm"), axis.ticks.length.x = unit(.25, "cm"), axis.text.x = element_text(margin = margin(t = .3, unit = "cm")))+

theme(legend.justification = "top")+

theme(legend.position = "right")+

theme(legend.key = element_rect(fill = "white"))+

theme(legend.title = element_text(face = NULL, size = 10))+theme(panel.background = element_blank(), axis.text = element_blank())+

theme(axis.text = element_text(colour = "black", size = 10)+

theme(axis.line = element_line())+

theme(panel.background = element_rect(fill = "white"),plot.margin = margin(0.5, 0.5, 0.5, 0.5, "cm"), plot.background = element_rect(colour = "grey"))+

theme(axis.ticks.length.y = unit(.25, "cm"), axis.ticks.length.x = unit(.25, "cm"), axis.text.x = element_text(margin = margin(t = .3, unit = "cm")))+

theme(axis.title.y = element_text(size = 10, face = "plain", angle = 90))+

theme(axis.title.x = element_text(size = 10, angle = 0)))#+stat_compare_means()

Pchao_l<-Pchao_l + aes(x = fct_inorder(Locations)) + theme(legend.position = "none") + xlab("Locations") + ylab("Chao1")

Pchao_l

**#####Shannon diversity estimates**

**###Extracting the shannon diversity index**

shannon <- diversity %>% dplyr::select(sample_id, diversity_shannon)

shannon_edited <- merge(shannon, sdata1, by = "sample_id", all = TRUE)

**###Confirming whetherthe Shannon indices are normally distributed**

shapiro.test(shannon_edited$diversity_shannon)

**###plotting the boxplots for the shannon index data**

P <- ggboxplot(shannon_edited, "Cropping_Type","diversity_shannon",

color = "Cropping_Type", palette =c("#F52100","#1B9E77", "#0079FF"),

add = "jitter", linetype = "solid", Family = "Palatino Linotype", add.params = list(),

error.plot = "pointrange", legand = NULL, size = NULL, width = 0.7, notch = FALSE, outlier.shape = 20, facet.by = NULL,

panel.labs = NULL, short.panel.labs = TRUE,bxp.errorbar = FALSE, bxp.errorbar.width = 0.4, ggtheme = theme_pubr())+

theme(axis.text.x = element_text(angle = 0, hjust = 0.5))+

theme(legend.text = element_text(size = 10, colour = "black", face = "italic"), legend.text.align = 0)+

theme(axis.text.y = element_text(angle = 0, vjust = 0.5, size = 10))+

theme(axis.text = element_text(colour = "black", size = 10))+

theme(axis.line = element_line())+

theme(panel.background = element_rect(fill = "white"),plot.margin = margin(0.5, 0.5, 0.5, 0.5, "cm"), plot.background = element_rect(colour = NULL, size = 1))+

theme(axis.ticks.length.y = unit(.25, "cm"), axis.ticks.length.x = unit(.25, "cm"), axis.text.x = element_text(margin = margin(t = .3, unit = "cm")))+

theme(legend.justification = "top")+

theme(legend.position = "right")+

theme(legend.key = element_rect(fill = "white"))+

theme(legend.title = element_text(face = NULL, size = 10))+theme(panel.background = element_blank(), axis.text = element_blank())+

theme(axis.text = element_text(colour = "black", size = 10)+

theme(axis.line = element_line())+

theme(panel.background = element_rect(fill = "white"),plot.margin = margin(0.5, 0.5, 0.5, 0.5, "cm"), plot.background = element_rect(colour = "grey"))+

theme(axis.ticks.length.y = unit(.25, "cm"), axis.ticks.length.x = unit(.25, "cm"), axis.text.x = element_text(margin = margin(t = .3, unit = "cm")))+

theme(axis.title.y = element_text(size = 10, face = "plain", angle = 90))+

theme(axis.title.x = element_text(size = 10, angle = 0))) #+stat_compare_means()

Pshan<-P + aes(x = fct_inorder(Cropping_Type)) + theme(legend.position = "none") + xlab("Cropping Type") + ylab("Shannon")

Pshan

**### Shannon diversity based on sampling locations**

**###Extracting the shannon diversity index**

shannon <- diversity %>% dplyr::select(sample_id, diversity_shannon)

shannon_edited <- merge(shannon, sdata1, by = "sample_id", all = TRUE)

#shannon_edited <- shannon_edited[c(1:18, 62:86, 19:61),]

**###Confirming whether the shannon indices are normally distributed**

shapiro.test(shannon_edited$diversity_shannon

**#Plotting the boxplots for the shannon index data**

P <- ggboxplot(shannon_edited, "Locations","diversity_shannon",

color = "Locations", palette =c("#F52100","#1B9E77", "#0079FF", "#F4CE14"),

add = "jitter", linetype = "solid", Family = "Palatino Linotype", add.params = list(),

error.plot = "pointrange", legand = NULL, size = NULL, width = 0.7, notch = FALSE, outlier.shape = 20, facet.by = NULL,

panel.labs = NULL, short.panel.labs = TRUE,bxp.errorbar = FALSE, bxp.errorbar.width = 0.4, ggtheme = theme_pubr())+

theme(axis.text.x = element_text(angle = 0, hjust = 0.5))+

theme(legend.text = element_text(size = 10, colour = "black", face = "italic"), legend.text.align = 0)+

theme(axis.text.y = element_text(angle = 0, vjust = 0.5, size = 10))+

theme(axis.text = element_text(colour = "black", size = 10))+

theme(axis.line = element_line())+

theme(panel.background = element_rect(fill = "white"),plot.margin = margin(0.5, 0.5, 0.5, 0.5, "cm"), plot.background = element_rect(colour = NULL, size = 1))+

theme(axis.ticks.length.y = unit(.25, "cm"), axis.ticks.length.x = unit(.25, "cm"), axis.text.x = element_text(margin = margin(t = .3, unit = "cm")))+

theme(legend.justification = "top")+

theme(legend.position = "right")+

theme(legend.key = element_rect(fill = "white"))+

theme(legend.title = element_text(face = NULL, size = 10))+theme(panel.background = element_blank(), axis.text = element_blank())+

theme(axis.text = element_text(colour = "black", size = 10)+

theme(axis.line = element_line())+

theme(panel.background = element_rect(fill = "white"),plot.margin = margin(0.5, 0.5, 0.5, 0.5, "cm"), plot.background = element_rect(colour = "grey"))+

theme(axis.ticks.length.y = unit(.25, "cm"), axis.ticks.length.x = unit(.25, "cm"), axis.text.x = element_text(margin = margin(t = .3, unit = "cm")))+

theme(axis.title.y = element_text(size = 10, face = "plain", angle = 90))+

theme(axis.title.x = element_text(size = 10, angle = 0))) #+stat_compare_means()

Pshan_1<-P + aes(x = fct_inorder(Locations)) + theme(legend.position = "none") + xlab("Locations") + ylab("Shannon")

Pshan_1

**#####Evenness metrics**

**####Extracting the evenness metrics**

even <- diversity %>% dplyr::select(sample_id, rarity_rare_abundance)

even

even_edited <- merge(even, sdata1, by = "sample_id", all = TRUE)

**####Confirming whether Evenness indices are normally distributed**

shapiro.test(even_edited$rarity_rare_abundance)

**####Plotting the boxplots for the evenness data**

P <- ggboxplot(even_edited, "Cropping_Type","rarity_rare_abundance",

color = "Cropping_Type", palette =c("#F52100","#1B9E77", "#0079FF"),

add = "jitter", linetype = "solid", Family = "Palatino Linotype", add.params = list(),

error.plot = "pointrange", legand = NULL, size = NULL, width = 0.7, notch = FALSE, outlier.shape = 20, facet.by = NULL,

panel.labs = NULL, short.panel.labs = TRUE,bxp.errorbar = FALSE, bxp.errorbar.width = 0.4, ggtheme = theme_pubr())+

theme(axis.text.x = element_text(angle = 0, hjust = 0.5))+

theme(legend.text = element_text(size = 10, colour = "black", face = "italic"), legend.text.align = 0)+

theme(axis.text.y = element_text(angle = 0, vjust = 0.5, size = 10))+

theme(axis.text = element_text(colour = "black", size = 10))+

theme(axis.line = element_line())+

theme(panel.background = element_rect(fill = "white"),plot.margin = margin(0.5, 0.5, 0.5, 0.5, "cm"), plot.background = element_rect(colour = NULL, size = 1))+

theme(axis.ticks.length.y = unit(.25, "cm"), axis.ticks.length.x = unit(.25, "cm"), axis.text.x = element_text(margin = margin(t = .3, unit = "cm")))+

theme(legend.justification = "top")+

theme(legend.position = "right")+

theme(legend.key = element_rect(fill = "white"))+

theme(legend.title = element_text(face = NULL, size = 10))+theme(panel.background = element_blank(), axis.text = element_blank())+

theme(axis.text = element_text(colour = "black", size = 10)+

theme(axis.line = element_line())+

theme(panel.background = element_rect(fill = "white"),plot.margin = margin(0.5, 0.5, 0.5, 0.5, "cm"), plot.background = element_rect(colour = "grey"))+

theme(axis.ticks.length.y = unit(.25, "cm"), axis.ticks.length.x = unit(.25, "cm"), axis.text.x = element_text(margin = margin(t = .3, unit = "cm")))+

theme(axis.title.y = element_text(size = 10, face = "plain", angle = 90))+

theme(axis.title.x = element_text(size = 10, angle = 0))) #+stat_compare_means()

Peven<-P + aes(x = fct_inorder(Cropping_Type)) + theme(legend.position = "none") + xlab("Cropping Type") + ylab("Evenness")

Peven

**######Evenness based on sampling location**

**####Extracting the Evenness**

even <- diversity %>% dplyr::select(sample_id, ACE)

even_edited <- merge(even, sdata1, by = "sample_id", all = TRUE)

**####Confirming whether the Evenness are normally distributed**

shapiro.test(even_edited$rarity_rare_abundance)

**####Plotting the boxplots for the Evenness data**

P <- ggboxplot(even_edited, "Locations","ACE",

color = "Locations", palette =c("#F52100","#1B9E77", "#0079FF", "#F4CE14"),

add = "jitter", linetype = "solid", Family = "Palatino Linotype", add.params = list(),

error.plot = "pointrange", legand = NULL, size = NULL, width = 0.7, notch = FALSE, outlier.shape = 20, facet.by = NULL,

panel.labs = NULL, short.panel.labs = TRUE,bxp.errorbar = FALSE, bxp.errorbar.width = 0.4, ggtheme = theme_pubr())+

theme(axis.text.x = element_text(angle = 0, hjust = 0.5))+

theme(legend.text = element_text(size = 10, colour = "black", face = "italic"), legend.text.align = 0)+

theme(axis.text.y = element_text(angle = 0, vjust = 0.5, size = 10))+

theme(axis.text = element_text(colour = "black", size = 10))+

theme(axis.line = element_line())+

theme(panel.background = element_rect(fill = "white"),plot.margin = margin(0.5, 0.5, 0.5, 0.5, "cm"), plot.background = element_rect(colour = NULL, size = 1))+

theme(axis.ticks.length.y = unit(.25, "cm"), axis.ticks.length.x = unit(.25, "cm"), axis.text.x = element_text(margin = margin(t = .3, unit = "cm")))+

theme(legend.justification = "top")+

theme(legend.position = "right")+

theme(legend.key = element_rect(fill = "white"))+

theme(legend.title = element_text(face = NULL, size = 10))+theme(panel.background = element_blank(), axis.text = element_blank())+

theme(axis.text = element_text(colour = "black", size = 10)+

theme(axis.line = element_line())+

theme(panel.background = element_rect(fill = "white"),plot.margin = margin(0.5, 0.5, 0.5, 0.5, "cm"), plot.background = element_rect(colour = "grey"))+

theme(axis.ticks.length.y = unit(.25, "cm"), axis.ticks.length.x = unit(.25, "cm"), axis.text.x = element_text(margin = margin(t = .3, unit = "cm")))+

theme(axis.title.y = element_text(size = 10, face = "plain", angle = 90))+

theme(axis.title.x = element_text(size = 10, angle = 0))) #+stat_compare_means()

Peven_1<-P + aes(x = fct_inorder(Locations)) + theme(legend.position = "none") + xlab("Locations") + ylab("Evenness")

Peven_1

ggarrange(Pchao,Pchao_l,

Pshan,Pshan_1,

Peven,Peven_1,

labels = c("A", "B", "C", "D", "E", "F"),

ncol = 3, nrow = 2)

ggsave("C:/Users/user/Desktop/RESULTS/Study group 2/ITS_General_Alpha_Diversity.jpeg",

width = 12, height = 12, dpi = 600)

ggsave("C:/Users/user/Desktop/RESULTS/Study group 2/ITS_General_Alpha_Diversity.png",

width = 12, height = 12, dpi = 600)

ggsave("C:/Users/user/Desktop/RESULTS/Study group 2/ITS_General_Alpha_Diversity.svg",

width = 12, height = 12, dpi = 600)

ggsave("C:/Users/user/Desktop/RESULTS/Study group 2/ITS_General_Alpha_Diversity.tiff",

width = 12, height = 12, dpi = 600)

**#####PERMANOVA based on samples type**

distme <- get_dist(ps4, distmethod ="bray", method="hellinger")

sampleda <- data.frame(sample_data(ps4), check.names=TRUE)

sampleda <- sampleda[match(colnames(as.matrix(distme)),rownames(sampleda)),,drop=FALSE]

sampleda$ Cropping_Type <- factor(sampleda$ Cropping_Type)

set.seed(9242)

adores <- adonis2(distme ~ Cropping_Type, data=sampleda, permutation=9999)

perm<-data.frame(adores$aov.tab)

write.csv(perm, "C:/Users/user/Desktop/RESULTS/Study group 2/ITS_Permutational_Multivariate_Analysis_Sampling_type.csv")

**#######PERMANOVA based on sampling location**

distme <- get_dist(ps4, distmethod ="bray", method="hellinger")

sampleda <- data.frame(sample_data(ps4), check.names=FALSE)

sampleda <- sampleda[match(colnames(as.matrix(distme)),row.names(sampleda)),,drop=FALSE]

sampleda$Locations <- factor(sampleda$Locations)

set.seed(1024)

sampleda

adores <- adonis2(distme~Locations, data=sampleda, permutation=9999)

distme

aov.tab

perm<-data.frame(adores$aov.tab)

write.csv(perm, " C:/Users/user/Desktop/RESULTS/Study group 2/ITS_Permutational_Multivariate_Analysis_Location.csv")

**####Beta diversity**

**####We now create a third object called random for merging with the other three object**

random_tree <- rtree(ntaxa(ps4), rooted = TRUE, tip.label = taxa_names(ps3))

plot(random_tree) ####visualize the rooted tree

**####Merging the preceding 3 objects and ploting the PCoA**

ps5 <- merge_phyloseq(ps4,random_tree)

ps5

ordu = phyloseq::ordinate(ps5, "PCoA", "unifrac", weighted = TRUE)

pa <- plot_ordination(ps5, ordu, color="Cropping_Type")+ geom_point(size=2) +

scale_color_manual(values = myPalette) +

theme(axis.text.x = element_text(angle = 0, hjust = 1))+

theme(legend.justification = "top")+

theme(legend.position = "right")+

theme(legend.key = element_rect(fill = "white"))+

theme(legend.text = element_text(size = rel(1), colour = "black"))+

theme(legend.title = element_text(face = NULL))+theme(panel.background = element_blank(), axis.text = element_blank())+

theme(axis.text = element_text(colour = "black", size = rel(1)))+

theme(axis.line = element_line())+

theme(panel.background = element_rect(fill = "white"),plot.margin = margin(0.5, 0.5, 0.5, 0.5, "cm"), plot.background = element_rect(colour = "grey"))

bA1<-pa + stat_ellipse() + theme(text = element_text(size = 14))

bA1

**####Beta diversity based on sampling location (PCoA)**

pb <- plot_ordination(ps5, ordu, color="Locations")+ geom_point(size=2) +

scale_color_manual(values = myPalette) +

theme(axis.text.x = element_text(angle = 0, hjust = 1))+

theme(legend.justification = "top")+

theme(legend.position = "right")+

theme(legend.key = element_rect(fill = "white"))+

theme(legend.text = element_text(size = rel(1), colour = "black"))+

theme(legend.title = element_text(face = NULL))+theme(panel.background = element_blank(), axis.text = element_blank())+

theme(axis.text = element_text(colour = "black", size = rel(1)))+

theme(axis.line = element_line())+

theme(panel.background = element_rect(fill = "white"),plot.margin = margin(0.5, 0.5, 0.5, 0.5, "cm"), plot.background = element_rect(colour = "grey"))

bB1<-pb + stat_ellipse() + theme(text = element_text(size = 14))

bB1

**#####Plotting the Venn diagram**

ps5

table(meta(ps5)$Cropping_Type)

**######Convert to relative abundances**

pseq.rel <- microbiome::transform(ps5, "compositional")

disease_states <- unique(as.character(meta(pseq.rel)$Cropping_Type))

print(disease_states)

list_core <- c() # an empty object to store information

for (n in disease_states){ # for each variable n in DiseaseState

#print(paste0("Identifying Core Taxa for ", n))

ps.sub <- subset_samples(pseq.rel, Cropping_Type == n) # Choose sample from DiseaseState by n

core_m <- core_members(ps.sub, # ps.sub is phyloseq selected with only samples from g

detection = 0.001, # 0.001 in atleast 90% samples

prevalence = 0.75)

print(paste0("No. of core taxa in ", n, " : ", length(core_m))) # print core taxa identified in each DiseaseState.

list_core[[n]] <- core_m # add to a list core taxon for each group.

**###Print core list**

print(list_core)

**####Specify colors and plot venn**

mycols<-c("GLD"="#CD9BCD", "SLD"="#6DDE88", "AID"="#3876BF")

venn_ct<-plot(venn(list_core),

fills = mycols,

#main="A",

legend = TRUE)

venn_ct

**###Venn diagrams based on sampling locations**

table(meta(ps5)$Locations)

# convert to relative abundances

pseq.rel <- microbiome::transform(ps5, "compositional")

disease_states <- unique(as.character(meta(pseq.rel)$Locations))

print(disease_states)

**####Print core list**

list_core <- c() # an empty object to store information

for (n in disease_states){ # for each variable n in DiseaseState

#print(paste0("Identifying Core Taxa for ", n))

ps.sub <- subset_samples(pseq.rel, Locations == n) # Choose sample from DiseaseState by n

core_m <- core_members(ps.sub, # ps.sub is phyloseq selected with only samples from g

detection = 0.001, # 0.001 in atleast 90% samples

prevalence = 0.75)

print(paste0("No. of core taxa in ", n, " : ", length(core_m))) # print core taxa identified in each DiseaseState.

list_core[[n]] <- core_m # add to a list core taxa for each group.

print(list_core)

**####Specify colors and plot venn**

mycols<-c("Vihiga"="#F7C8E0", "Siaya"="#DFFFD8", "Homabay"="#B4E4FF", "Kisumu"="#95BDFF")

venn_lc<-plot(venn(list_core),

fills = mycols,

#main="A",

legend = TRUE)

venn_lc

**###Combine the PCoA and Venn diagrams in one image**

ggarrange(bA1,bB1,venn_ct, venn_lc,

labels = c("A", "B", "C"),

ncol = 2, nrow = 2)

C:/Users/user/Desktop/RESULTS/Study group 2/ITS_

ggsave("C:/Users/user/Desktop/RESULTS/Study group 2/ITS_Beta.jpeg",

width = 12, height = 12, dpi = 600)

ggsave("C:/Users/user/Desktop/RESULTS/Study group 2/ITS_Beta.png",

width = 12, height = 12, dpi = 600)

ggsave("C:/Users/user/Desktop/RESULTS/Study group 2/ITS_Beta.svg",

width = 12, height = 12, dpi = 600)

ggsave("C:/Users/user/Desktop/RESULTS/Study group 2/ITS_Beta.tiff",

width = 12, height = 12, dpi = 600)
