# Supplementary material for: Comparative microbiome diversity in root-nodules of three Desmodium species used in push-pull cropping system
Source: Front Microbiol. 2024 Jun 20;15:1395811. doi: 10.3389/fmicb.2024.1395811 (PMC11222577; doi:10.3389/fmicb.2024.1395811)
Supplement: Supplementary file 3 [file Table_3.docx]

**Comparative Microbiome Diversity in Root-Nodules of Three *Desmodium* Species Used in Push-Pull Cropping System**

Isack H. Adan^1^**^,^**^2^, George Ochieng Asudi^2^, Saliou Niassy^3^, Abdul A. Jalloh^1,4^, Johnstone Mutiso Mutua^1^, Frank Chidawanyika^1,6^, Fathiya Khamis^1^, Zeyaur Khan^1^, Sevgan Subramanian^1^, Thomas Dubois^1^, Daniel Munyao Mutyambai^1,5^**^*^**

^1^International Centre of Insect Physiology and Ecology, P.O. Box 30772-00100 Nairobi, Kenya.

^2^Department of Biochemistry, Microbiology and Biotechnology, Kenyatta University, P.O. Box 43844-00100 Nairobi, Kenya.

^3^Inter-African Phytosanitary Council of the African Union, P.O Box 4170, Yaoundé, Cameroon.

^4^Department of Zoology and Entomology, University of Pretoria, Pretoria Private Bag x20 Hatfield, Pretoria, South Africa.

^5^Department of Life Sciences, South Eastern Kenya University, P.O. Box 170-90200 Kitui, Kenya.

^6^Department of Zoology and Entomology, University of Free State, Bloemfontein, South Africa.

*****Corresponding author: [dmutyambai@icipe.org](mailto:dmutyambai@icipe.org)

**Additional files**

**Supplementary figures**


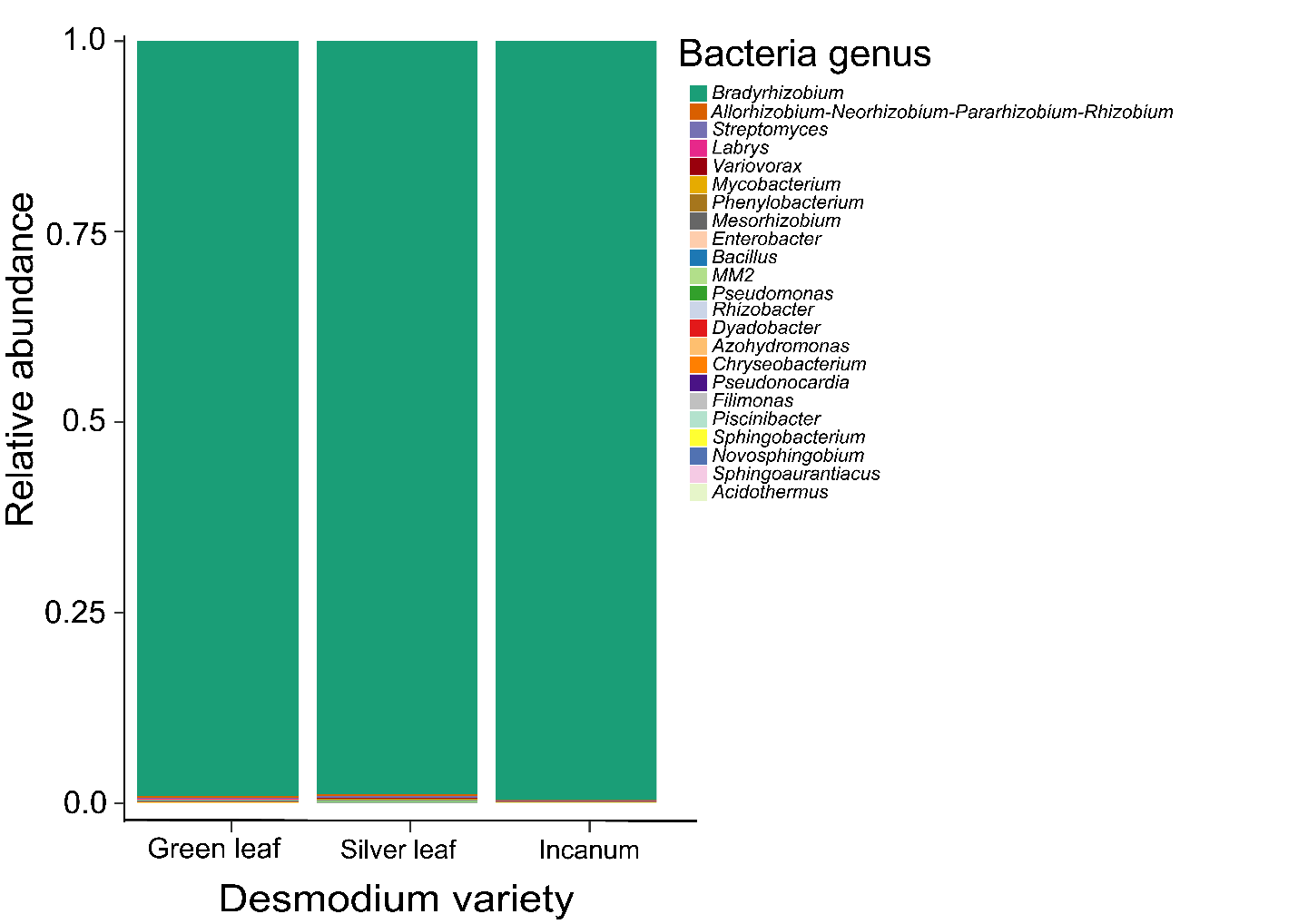


**Figure S1:** Barplots representing the relative abundance of the predominant bacterial genera observed across all the three *Desmodium* species used in push-pull cropping systems. *Desmodium intortum* (Greenleaf, GLD), *Desmodium uncinatum* (Silverleaf, SLD) and *Desmodium incanum* (incanum, AID).


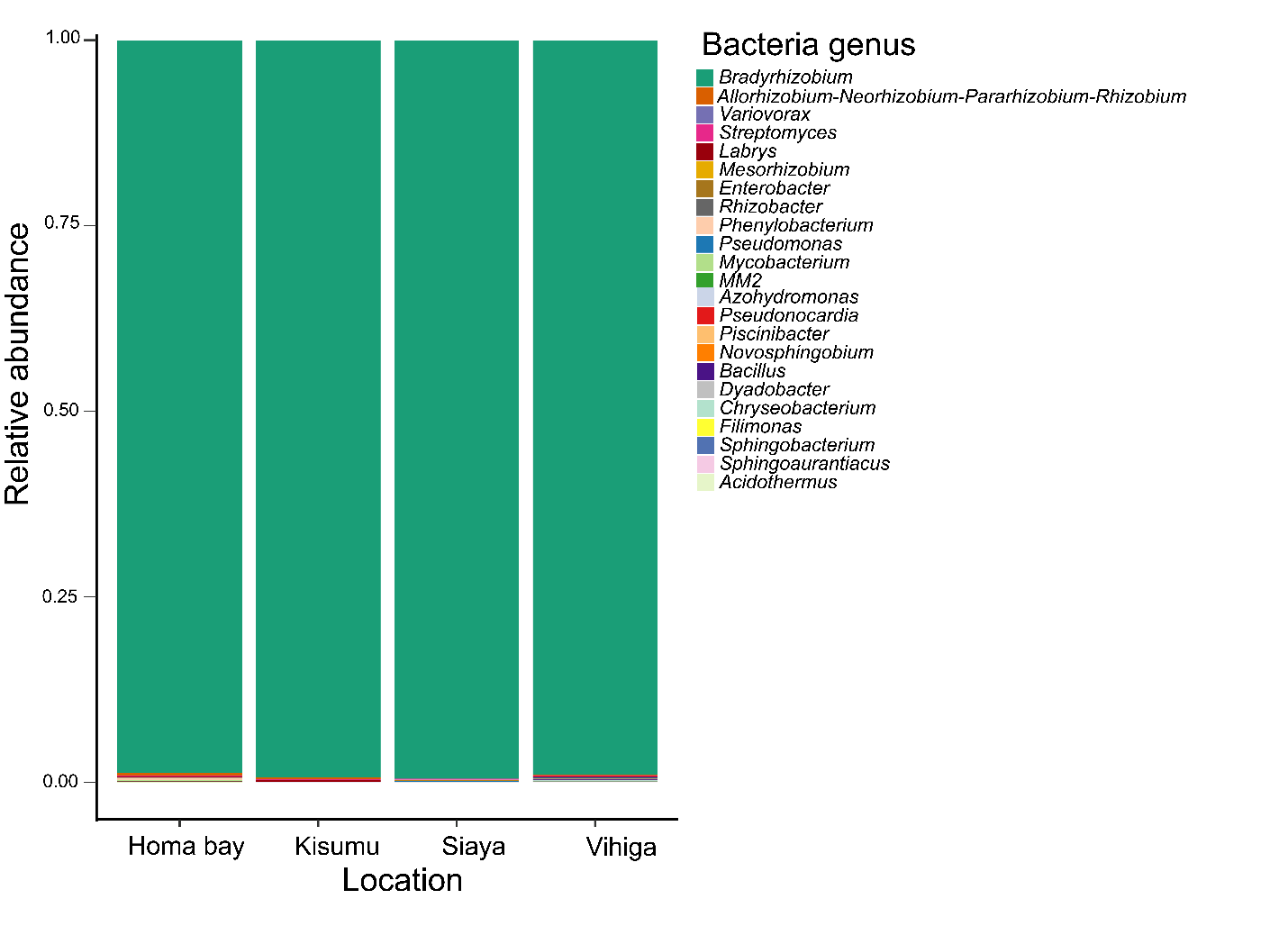


**Figure S2:** Barplots representing the relative abundance of the predominant bacterial amplicon sequence variants (ASVs) at the genus level observed across all the four sampling locations.


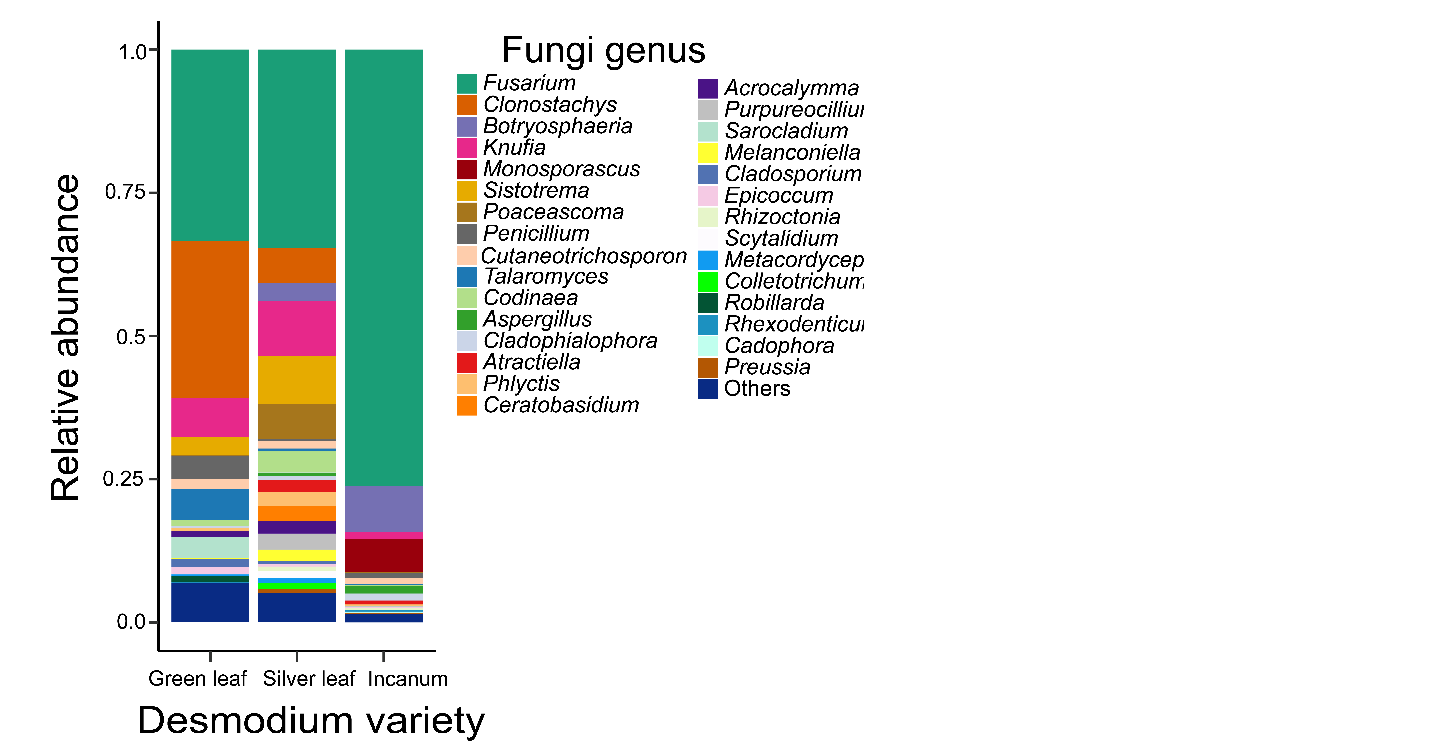


**Figure S3:** Barplots representing the relative abundance of the predominant fungal ASVs at the genus level observed across all the three *Desmodium* species used in push-pull cropping systems. *Desmodium intortum* (Greenleaf, GLD), *Desmodium uncinatum* (Silverleaf, SLD) and *Desmodium incanum* (incanum, AID).


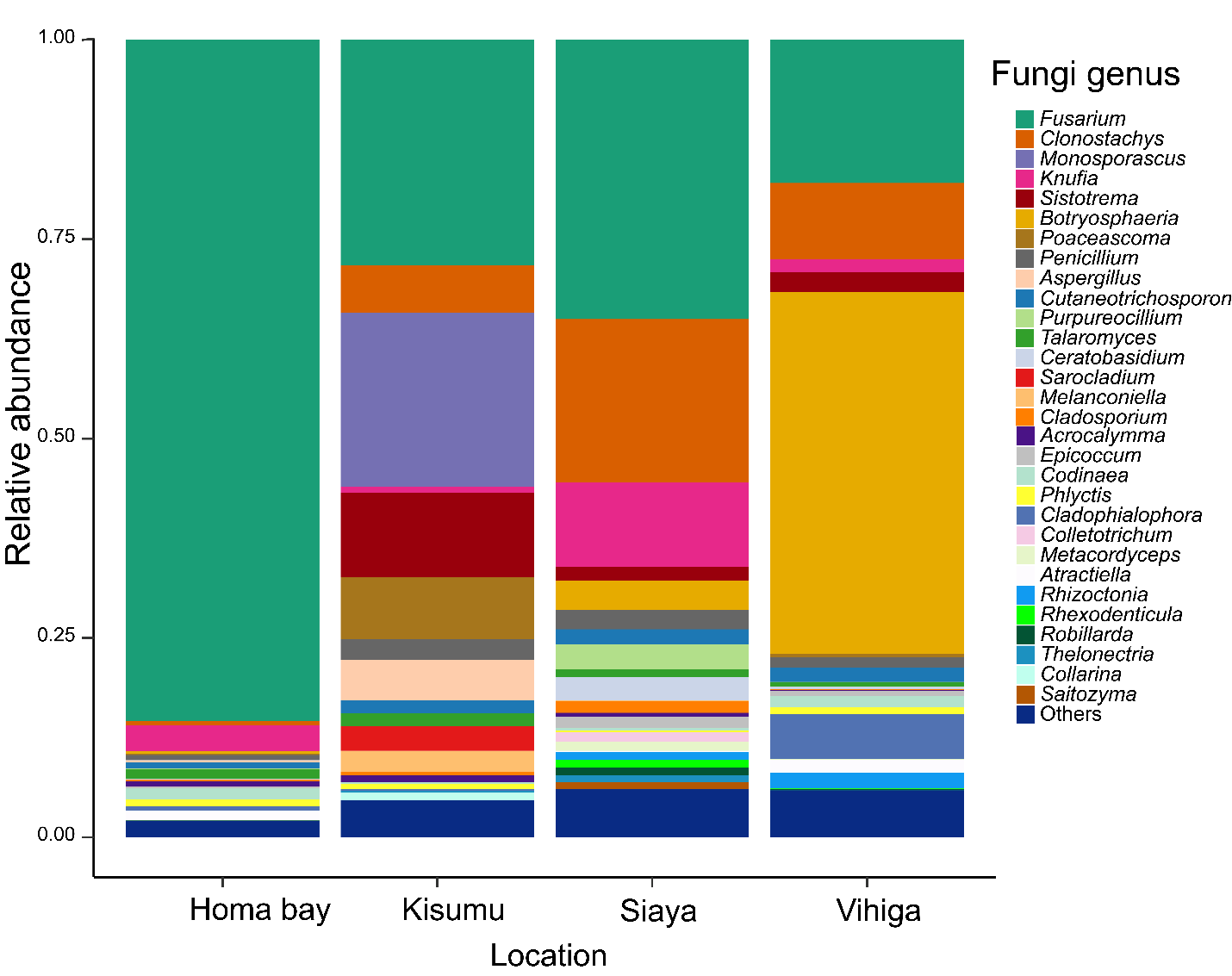


**Figure S4:** Barplots representing the relative abundance of the predominant fungal amplicon sequence variants (ASVs) at the genus level observed across all the four sampling locations.

**Supplementary tables**

**Table S1**: Global positioning system coordinates of the sampling locations in Vihiga, Siaya, Kisumu and Homabay counties in Western Kenya

| **Sample** | **County** | **Latitude** | **longitude** | **Elevation (m)** | **Variety** | **Relative humidity** | **Soil temperature** | **Date of collection** |
| --- | --- | --- | --- | --- | --- | --- | --- | --- |
| 1 | Vihiga | 0.081058 | 34.56054 | 1403.0 | GLD | 75.6125 | 22.1536 | 7/11/2022 |
| 2 | Vihiga | 0.040469 | 34.57405 | 1500.8 | AID | 76.5893 | 21.4222 | 7/11/2022 |
| 3 | Siaya | 0.054792 | 34.28757 | 1319.0 | GLD | 77.1896 | 22.2574 | 4/11/2022 |
| 4 | Kisumu | -0.05045 | 34.63425 | 1387.6 | SLD | 74.8423 | 22.2760 | 6/11/2022 |
| 5 | Vihiga | 0.071732 | 34.5779 | 1519.5 | SLD | 75.3942 | 21.6142 | 7/11/2022 |
| 6 | Siaya | 0.188639 | 34.3608 | 1306.4 | AID | 76.8223 | 21.5277 | 4/11/2022 |
| 7 | Homabay | -0.38229 | 34.17545 | 1137.6 | AID | 70.4724 | 19.0910 | 2/12/2022 |
| 8 | Homabay | -0.53816 | 34.33304 | 1221.5 | GLD | 72.8167 | 23.6028 | 2/11/2022 |
| 9 | Homabay | -0.56169 | 34.31144 | 1166.5 | GLD | 72.8729 | 23.9538 | 2/11/2022 |
| 10 | Vihiga | 0.011884 | 34.59165 | 1519.0 | GLD | 76.3987 | 21.4828 | 7/11/2022 |
| 11 | Siaya | -0.00028 | 34.27183 | 1253.0 | SLD | 78.2644 | 22.5504 | 4/11/2022 |
| 12 | Siaya | 0.027154 | 34.3086 | 1363.0 | AID | 77.1553 | 22.0104 | 4/11/2022 |
| 13 | Siaya | 0.000061 | 34.27034 | 1253.4 | GLD | 77.2517 | 22.7059 | 4/11/2022 |
| 14 | Homabay | -0.43228 | 34.20838 | 1153.0 | SLD | 74.9047 | 24.4120 | 2/11/2022 |
| 15 | Siaya | 0.027336 | 34.3091 | 1319.0 | GLD | 77.1553 | 22.0104 | 4/11/2022 |
| 16 | Kisumu | -0.04551 | 34.6378 | 1372.0 | AID | 74.8677 | 22.4450 | 6/11/2022 |
| 17 | Siaya | 0.0408 | 34.30542 | 1336.2 | SLD | 77.1734 | 22.1404 | 4/11/2022 |
| 18 | Siaya | -0.0008 | 34.27038 | 1253.3 | AID | 78.2722 | 22.6089 | 4/11/2022 |
| 19 | Siaya | 0.027652 | 34.30997 | 1361.0 | GLD | 77.1544 | 22.0039 | 4/12/2022 |
| 20 | Siaya | 0.02833 | 34.31009 | 1263.0 | SLD | 77.1526 | 21.9909 | 4/11/2022 |
| 21 | Kisumu | 0.003787 | 34.60012 | 1529.4 | AID | 76.3896 | 21.4178 | 6/11/2022 |
| 22 | Siaya | 0.18865 | 34.3609 | 1306.6 | AID | 76.8205 | 21.5147 | 4/11/2022 |
| 23 | Kisumu | -0.03239 | 34.61042 | 1436.5 | SLD | 74.8032 | 22.0160 | 6/11/2022 |
| 24 | Kisumu | -0.04651 | 34.64606 | 1353.0 | SLD | 74.8765 | 22.5035 | 6/11/2022 |

Silverleaf desmodium (SLD), *Desmodium uncinatum*; Green leaf desmodium (GLD), *Desmodium intortum*; and African desmodium (AID), *Desmodium incanum*; m, meter.

**Table S2:** Relative percentage abundance of bacterial genera in the root-nodules of the three *Desmodium* species

| **G/NO** | **Genus** | **GLD** | **SLD** | **AID** |
| --- | --- | --- | --- | --- |
| 1 | *Acidothermus* | 0.00 | 0.00 | 0.00 |
| 2 | *Allorhizobium-Neorhizobium-Pararhizobium-Rhizobium* | 0.20 | 0.20 | 0.10 |
| 3 | *Azohydromonas* | 0.00 | 0.00 | 0.00 |
| 4 | *Bacillus* | 0.10 | 0.00 | 0.00 |
| 5 | *Bradyrhizobium* | 99.10 | 98.90 | 99.60 |
| 6 | *Chryseobacterium* | 0.00 | 0.00 | 0.00 |
| 7 | *Dyadobacter* | 0.00 | 0.00 | 0.00 |
| 8 | *Enterobacter* | 0.00 | 0.10 | 0.00 |
| 9 | *Filimonas* | 0.00 | 0.00 | 0.00 |
| 10 | *Labrys* | 0.20 | 0.00 | 0.10 |
| 11 | *Mesorhizobium* | 0.10 | 0.00 | 0.00 |
| 12 | *MM2* | 0.00 | 0.10 | 0.00 |
| 13 | *Mycobacterium* | 0.10 | 0.10 | 0.00 |
| 14 | *Novosphingobium* | 0.00 | 0.00 | 0.00 |
| 15 | *Phenylobacterium* | 0.00 | 0.00 | 0.10 |
| 16 | *Piscinibacter* | 0.00 | 0.00 | 0.00 |
| 17 | *Pseudomonas* | 0.00 | 0.00 | 0.00 |
| 18 | *Pseudonocardia* | 0.00 | 0.00 | 0.00 |
| 19 | *Rhizobacter* | 0.00 | 0.00 | 0.00 |
| 20 | *Sphingoaurantiacus* | 0.00 | 0.00 | 0.00 |
| 21 | *Sphingobacterium* | 0.00 | 0.00 | 0.00 |
| 22 | *Streptomyces* | 0.10 | 0.20 | 0.00 |
| 23 | *Variovorax* | 0.00 | 0.20 | 0.00 |
|  | **Total** | **100.00** | **100.00** | **100.00** |

Silverleaf desmodium (SLD), *Desmodium* *uncinatum*; Greenleaf desmodium (GLD), *Desmodium* *intortum*; and African desmodium (AID), *Desmodium incanum*.

**Table S3:** Relative abundance of bacterial genera in the root-nodules of the three *Desmodium* species as influenced by the sampling locations

| **G/NO** | **Genus** | **Homabay** | **Kisumu** | **Siaya** | **Vihiga** |
| --- | --- | --- | --- | --- | --- |
| 1 | *Acidothermus* | 0.10 | 0.00 | 0.00 | 0.00 |
| 2 | *Allorhizobium-Neorhizobium-Pararhizobium-Rhizobium* | 0.40 | 0.30 | 0.10 | 0.20 |
| 3 | *Azohydromonas* | 0.00 | 0.10 | 0.00 | 0.00 |
| 4 | *Bacillus* | 0.10 | 0.00 | 0.00 | 0.10 |
| 5 | *Bradyrhizobium* | 98.70 | 99.40 | 99.40 | 98.90 |
| 6 | *Chryseobacterium* | 0.00 | 0.00 | 0.00 | 0.10 |
| 7 | *Dyadobacter* | 0.00 | 0.00 | 0.00 | 0.10 |
| 8 | *Enterobacter* | 0.00 | 0.00 | 0.10 | 0.10 |
| 9 | *Filimonas* | 0.00 | 0.00 | 0.00 | 0.10 |
| 10 | *Labrys* | 0.20 | 0.20 | 0.00 | 0.10 |
| 11 | *Mesorhizobium* | 0.00 | 0.00 | 0.10 | 0.00 |
| 12 | *MM2* | 0.10 | 0.00 | 0.00 | 0.00 |
| 13 | *Mycobacterium* | 0.10 | 0.00 | 0.00 | 0.10 |
| 14 | *Novosphingobium* | 0.00 | 0.00 | 0.00 | 0.00 |
| 15 | *Phenylobacterium* | 0.30 | 0.00 | 0.00 | 0.00 |
| 16 | *Piscinibacter* | 0.00 | 0.00 | 0.00 | 0.00 |
| 17 | *Pseudomonas* | 0.00 | 0.00 | 0.00 | 0.10 |
| 18 | *Pseudonocardia* | 0.00 | 0.00 | 0.00 | 0.00 |
| 19 | *Rhizobacter* | 0.00 | 0.00 | 0.00 | 0.00 |
| 20 | *Sphingoaurantiacus* | 0.00 | 0.00 | 0.00 | 0.10 |
| 21 | *Sphingobacterium* | 0.00 | 0.00 | 0.00 | 0.10 |
| 22 | *Streptomyces* | 0.10 | 0.10 | 0.10 | 0.10 |
| 23 | *Variovorax* | 0.00 | 0.00 | 0.10 | 0.00 |
|  | **Total** | **100.00** | **100.00** | **100.00** | **100.00** |

**Table S4:** Relative percentage abundance of bacterial species in the root-nodules of the three *Desmodium* species

| **S/No** | **Species** | **GLD** | | **SLD** | | **AID** | |
| --- | --- | --- | --- | --- | --- | --- | --- |
| 1 | *Allorhizobium-Neorhizobium-Pararhizobium-Rhizobium phaseoli* | 0.00 | | 0.10 | | 0.00 | |
| 2 | *Allorhizobium-Neorhizobium-Pararhizobium-Rhizobium* spp | 0.20 | | 0.20 | | 0.10 | |
| 3 | *Azohydromonas spp* | 0.00 | | 0.00 | | 0.00 | |
| 4 | *Bacillus* spp | 0.10 | | 0.00 | | 0.00 | |
| 5 | *Bradyrhizobium elkanii* | 33.70 | | 38.20 | | 10.00 | |
| 6 | *Bradyrhizobium japonicum* | 3.30 | | 4.40 | | 5.70 | |
| 7 | *Bradyrhizobium liaoningense* | 16.60 | | 23.00 | | 27.70 | |
| 8 | *Bradyrhizobium* spp | 32.10 | | 30.90 | | 44.10 | |
| 9 | *Bradyrhizobium yuanmingense* | 13.40 | | 2.30 | | 12.20 | |
| 10 | *Chryseobacterium indologenes* | 0.00 | | 0.00 | | 0.00 | |
| 11 | *Dyadobacter fermentans* | 0.00 | | 0.00 | | 0.00 | |
| 12 | *Enterobacter kobei* | 0.00 | | 0.10 | | 0.00 | |
| 13 | *Filimonas* spp | 0.00 | | 0.00 | | 0.00 | |
| 14 | *Labrys neptuniae* | 0.20 | | 0.00 | | 0.00 | |
| 15 | *Labrys* spp | 0.00 | | 0.00 | | 0.10 | |
| 16 | *Mesorhizobium plurifarium* | 0.00 | | 0.00 | | 0.00 | |
| 17 | *Mesorhizobium* spp | 0.00 | | 0.00 | | 0.00 | |
| 18 | *MM2* spp | 0.00 | | 0.10 | | 0.00 | |
| 19 | *Mycobacterium moriokaense* | 0.00 | | 0.00 | | 0.00 | |
| 20 | *Mycobacterium neoaurum* | 0.00 | | 0.10 | | 0.00 | |
| 21 | *Mycobacterium* spp | 0.00 | | 0.00 | | 0.00 | |
| 22 | *Phenylobacterium* spp | 0.00 | | 0.00 | | 0.10 | |
| 23 | *Piscinibacter* spp | 0.00 | | 0.00 | | 0.00 | |
| 24 | *Pseudomonas* spp | 0.00 | | 0.00 | | 0.00 | |
| 25 | *Pseudonocardia dioxanivorans* | 0.00 | | 0.00 | | 0.00 | |
| 26 | *Rhizobacter* spp | 0.00 | | 0.00 | | 0.00 | |
| 27 | *Sphingobacterium multivorum* | 0.00 | | 0.00 | | 0.00 | |
| 28 | *Streptomyces griseorubiginosus* | 0.00 | | 0.10 | | 0.00 | |
| 29 | *Streptomyces* spp | 0.00 | | 0.00 | | 0.00 | |
| 30 | *Variovorax paradoxus* | 0.00 | | 0.20 | | 0.00 | |
|  | Others | | 0.10 | | 0.00 | | 0.00 |
|  | **Total** | **100.00** | | **100.00** | | **100.00** | |

Silverleaf desmodium (SLD), *Desmodium* *uncinatum*; Green leaf desmodium (GLD), *Desmodium* *intortum*; and African desmodium (AID), *Desmodium incanum*.

**Table S5:** Relative percentage abundance of bacterial species in the root-nodules of the three *Desmodium* species as influenced by sampling location

| **S/No** | **Species** | **Homabay** | **Kisumu** | **Siaya** | **Vihiga** |
| --- | --- | --- | --- | --- | --- |
| 1 | *Allorhizobium-Neorhizobium-Pararhizobium-Rhizobium phaseoli* | 0.00 | 0.00 | 0.10 | 0.00 |
| 2 | *Allorhizobium-Neorhizobium-Pararhizobium-Rhizobium* spp | 0.40 | 0.30 | 0.00 | 0.20 |
| 3 | *Azohydromonas* spp | 0.00 | 0.10 | 0.00 | 0.00 |
| 4 | *Bacillus* spp | 0.10 | 0.00 | 0.00 | 0.10 |
| 5 | *Bradyrhizobium elkanii* | 36.20 | 8.70 | 25.80 | 50.40 |
| 6 | *Bradyrhizobium japonicum* | 3.70 | 3.00 | 5.50 | 3.60 |
| 7 | *Bradyrhizobium liaoningense* | 18.40 | 16.20 | 27.40 | 17.30 |
| 8 | *Bradyrhizobium* spp | 38.50 | 34.10 | 39.20 | 24.20 |
| 9 | *Bradyrhizobium yuanmingense* | 2.00 | 37.40 | 1.50 | 3.50 |
| 10 | *Chryseobacterium indologenes* | 0.00 | 0.00 | 0.00 | 0.10 |
| 11 | *Dyadobacter fermentans* | 0.00 | 0.00 | 0.00 | 0.10 |
| 12 | *Enterobacter kobei* | 0.00 | 0.00 | 0.10 | 0.00 |
| 13 | *Filimonas spp* | 0.00 | 0.00 | 0.00 | 0.10 |
| 14 | *Labrys neptuniae* | 0.00 | 0.20 | 0.00 | 0.10 |
| 15 | *Labrys* spp | 0.20 | 0.00 | 0.00 | 0.00 |
| 16 | *Mesorhizobium plurifarium* | 0.00 | 0.00 | 0.00 | 0.00 |
| 17 | *Mesorhizobium* spp | 0.00 | 0.00 | 0.00 | 0.00 |
| 18 | *MM2* spp | 0.10 | 0.00 | 0.00 | 0.00 |
| 19 | *Mycobacterium neoaurum* | 0.10 | 0.00 | 0.00 | 0.00 |
| 20 | *Mycobacterium* spp | 0.00 | 0.00 | 0.00 | 0.10 |
| 21 | *Novosphingobium aromaticivorans* | 0.00 | 0.00 | 0.00 | 0.00 |
| 23 | *Phenylobacterium* spp | 0.30 | 0.00 | 0.00 | 0.00 |
| 24 | *Piscinibacter* spp | 0.00 | 0.00 | 0.00 | 0.00 |
| 25 | *Pseudomonas* spp | 0.00 | 0.00 | 0.00 | 0.10 |
| 26 | *Rhizobacter* spp | 0.00 | 0.00 | 0.00 | 0.00 |
| 27 | *Sphingobacterium multivorum* | 0.00 | 0.00 | 0.00 | 0.10 |
| 28 | *Streptomyces griseorubiginosus* | 0.10 | 0.00 | 0.10 | 0.00 |
| 29 | *Streptomyces* spp | 0.00 | 0.10 | 0.00 | 0.10 |
| 30 | *Variovorax paradoxus* | 0.00 | 0.00 | 0.10 | 0.00 |
|  | Others | 0.10 | 0.00 | 0.00 | 0.10 |
|  | **Total** | **100.00** | **100.00** | **100.00** | **100.00** |

**Table S6**: Pairwise total sum scaling (TSS, relative abundance) log2 linear regression (*P* value) of top five bacterial genera in the root-nodules of the three *Desmodium* species

| **Genus** | **AID vs SLD** | **AID vs GLD** | **GLD vs SLD** |
| --- | --- | --- | --- |
| *Bradyrhizobium* | 0.412 | 0.694 | 0.881 |
| *Bacillus* | 0.852 | 0.154 | 0.225 |
| *Enterobacter* | 0.804 | 0.334 | 0.334 |
| *Labrys* | 0.923 | 0.811 | 0.933 |
| *mesorhizobium* | 0.663 | 0.633 | 0.948 |
| *mycobacterium* | 0.366 | 0.423 | 0.930 |
| *phenylobacterium* | 0.137 | 0.106 | 0.854 |
| *streptomyces* | 0.237 | 0.453 | 0.547 |
| *variovorax* | 1 | N/A | 0.1516 |

Silverleaf desmodium (SLD), *Desmodium uncinatum*; Green leaf desmodium (GLD), *Desmodium intortum*; and African desmodium (AID), *Desmodium incanum*; N/A, negligible.

**Table S7**: Pairwise total sum scaling (TSS, relative abundance) log2 linear regression (*P* value) of top five bacterial species in the root-nodules of the three *Desmodium* species

| **Species** | **AID vs SLD** | **AID vs GLD** | **GLD vs SLD** |
| --- | --- | --- | --- |
| *Bradyrhizobium elkanii* | 0.070 | 0.213 | 0.616 |
| *Bradyrhizobium* spp | 0.208 | 0.359 | 0.568 |
| *Bradyrhizobium liaoningense* | 0.308 | 0.796 | 0.356 |
| *Bradyrhizobium yuanmingense* | 0.199 | 0.279 | 0.809 |
| *Bradyrhizobium japonicum* | 0.381 | 0.891 | 0.378 |

Silverleaf desmodium (SLD), *Desmodium uncinatum*; Green leaf desmodium (GLD), *Desmodium intortum*; and African desmodium (AID), *Desmodium incanum*.

**Table S8**: Pairwise total sum scaling (TSS, relative abundance) log2 linear regression (*P* value) of top five bacterial genera based on sampling locations

| **Genus** | **Homabay vs Kisumu** | **Homabay vs Siaya** | **Homabay vs Vihiga** | **Kisumu vs Siaya** | **Kisumu vs Vihiga** | **Siaya vs Vihiga** |
| --- | --- | --- | --- | --- | --- | --- |
| *Bradyrhizobium* | 0.073 | 0.601 | 0.018***** | 0.601 | 0.451 | 0.331 |
| *Allorhizobium-Neorhizobium-Pararhizobium-Rhizobium* | 0.293 | 0.387 | 0.224 | 0.534 | 0.927 | 0.430 |
| *Phenylobacterium* | 0.389 | 0.990 | 0.292 | 0.167 | 0.091 | 0.168 |
| *Labrys* | 0.828 | 0.580 | 0.093 | 0.341 | 0.012 | 0.075 |
| *Bacillus* | N/A | 0.172 | N/A | 0.173 | N/A | 0.128 |
| *Azohydromonas* | N/A | N/A | 0.4071 | N/A | 0.407 | 0.143 |
| *Streptomyces* | 0.198 | 0.195 | 0.015***** | 0.526 | 0.292 | 0.052 |
| *Enterobacter* | N/A | 0.406 | N/A | 0.406 | N/A | 0.351 |
| *Mesorhizobium* | 0.134 | 0.133 | 0.089 | 0.407 | N/A | 0.356 |
| *Variovorax* | 0.356 | 0.098 | 0.587 | N/A | 0.193 | 0.024 |
| *Chryseobacterium* | 0.356 | 0.890 | 0.292 | 0.566 | 0.566 | 0.519 |
| *Dyadobacter* | N/A | 0.566 | N/A | 0.566 | N/A | 0.519 |
|  |  |  |  |  |  |  |

N/A, negligible; Asterisk represents a significant difference (* *P* < 0.005)

**Table S9**: Pairwise total sum scaling (TSS, relative abundance) log2 linear regression (*P* value) of top five bacterial species based on sampling location (county)

| **Species** | **Homabay vs Kisumu** | **Homabay vs Siaya** | **Homabay vs Vihiga** | **Kisumu vs Siaya** | **Kisumu vs Vihiga** | **Siaya vs Vihiga** |
| --- | --- | --- | --- | --- | --- | --- |
| *Bradyrhizobium elkanii* | 0.353 | 0.015 | 0.157 | 0.202 | 0.579 | 0.616 |
| *Bradyrhizobium spp* | 0.009 | 0.676 | 0.108 | 0.275 | 0.123 | 0.669 |
| *Bradyrhizobium liaoningense* | 0.011 | 0.0002***** | 0.005***** | 0.207 | 0.379 | 0.355 |
| *Bradyrhizobium yuanmingense* | 0.117 | 0.024 | 0.227 | 0.917 | 0.617 | 0.524 |
| *Bradyrhizobium japonicum* | 0.007 | 0.0002***** | 0.004***** | 0.204 | 0.254 | 0.445 |

Asterisk represents a significant difference (* *P* < 0.005)

**Table S10:** Relative percentage abundance of fungal genera in the root-nodules of the three *Desmodium* species

| **G/NO** | **Genus** | **GLD** | **SLD** | **AID** |
| --- | --- | --- | --- | --- |
| 1 | *Acrocalymma* | 1.00 | 2.20 | 0.00 |
| 2 | *Aspergillus* | 0.00 | 0.70 | 1.30 |
| 3 | *Atractiella* | 0.00 | 2.10 | 0.70 |
| 4 | *Botryosphaeria* | 0.00 | 3.20 | 8.00 |
| 5 | *Cadophora* | 0.00 | 0.00 | 0.30 |
| 6 | *Ceratobasidium* | 0.00 | 2.60 | 0.10 |
| 7 | *Cladophialophora* | 0.30 | 0.70 | 1.20 |
| 8 | *Cladosporium* | 1.50 | 0.60 | 0.10 |
| 9 | *Clonostachys* | 27.40 | 6.10 | 0.00 |
| 10 | *Codinaea* | 1.10 | 3.70 | 0.10 |
| 11 | *Colletotrichum* | 0.00 | 1.00 | 0.00 |
| 12 | *Cutaneotrichosporon* | 1.80 | 1.40 | 1.00 |
| 13 | *Epicoccum* | 1.30 | 0.40 | 0.20 |
| 14 | *Fusarium* | 33.40 | 34.60 | 76.10 |
| 15 | *Knufia* | 6.90 | 9.60 | 1.30 |
| 16 | *Melanconiella* | 0.20 | 2.00 | 0.00 |
| 17 | *Metacordyceps* | 0.20 | 0.80 | 0.00 |
| 18 | *Monosporascus* | 0.00 | 0.00 | 5.70 |
| 19 | *Penicillium* | 4.00 | 0.30 | 1.00 |
| 20 | *Phlyctis* | 0.50 | 2.50 | 0.20 |
| 21 | *Poaceascoma* | 0.30 | 6.00 | 0.10 |
| 22 | *Preussia* | 0.00 | 0.70 | 0.10 |
| 23 | *Purpureocillium* | 0.00 | 2.80 | 0.00 |
| 24 | *Rhexodenticula* | 0.20 | 0.00 | 0.30 |
| 25 | *Rhizoctonia* | 0.00 | 0.80 | 0.30 |
| 26 | *Robillarda* | 1.10 | 0.10 | 0.00 |
| 27 | *Sarocladium* | 3.60 | 0.00 | 0.00 |
| 28 | *Scytalidium* | 0.00 | 1.30 | 0.00 |
| 29 | *Sistotrema* | 3.10 | 8.40 | 0.00 |
| 30 | *Talaromyces* | 5.40 | 0.50 | 0.20 |
|  | Others | 6.90 | 5.10 | 1.50 |
|  | **Total** | **100.00** | **100.00** | **100.00** |

Silverleaf desmodium (SLD), *Desmodium* *uncinatum*; Green leaf desmodium (GLD), *Desmodium* *intortum*; and African desmodium (AID), *Desmodium incanum*.

**Table S11:** Relative percentage abundance of fungal genera in the root-nodules of the three *Desmodium* species as influenced by sampling locations

| **G/NO** | **Genus** | **Homabay** | **Kisumu** | **Siaya** | **Vihiga** |
| --- | --- | --- | --- | --- | --- |
| 1 | *Acrocalymma* | 0.70 | 0.90 | 0.50 | 0.10 |
| 2 | *Aspergillus* | 0.30 | 5.10 | 0.00 | 0.00 |
| 3 | *Atractiella* | 1.20 | 0.00 | 0.20 | 1.70 |
| 4 | *Botryosphaeria* | 0.30 | 0.00 | 3.70 | 45.40 |
| 5 | *Ceratobasidium* | 0.10 | 0.00 | 2.90 | 0.40 |
| 6 | *Cladophialophora* | 0.50 | 0.20 | 0.10 | 5.60 |
| 7 | *Cladosporium* | 0.20 | 0.50 | 1.40 | 0.10 |
| 8 | *Clonostachys* | 0.50 | 5.90 | 20.50 | 9.60 |
| 9 | *Codinaea* | 1.40 | 0.30 | 0.20 | 1.40 |
| 10 | *Collarina* | 0.00 | 1.00 | 0.00 | 0.00 |
| 11 | *Colletotrichum* | 0.00 | 0.00 | 1.20 | 0.00 |
| 12 | *Cutaneotrichosporon* | 0.80 | 1.60 | 1.90 | 1.80 |
| 13 | *Epicoccum* | 0.10 | 0.00 | 1.50 | 0.60 |
| 14 | *Fusarium* | 85.40 | 28.30 | 35.00 | 17.90 |
| 15 | *Knufia* | 3.20 | 0.80 | 10.60 | 1.60 |
| 16 | *Melanconiella* | 0.00 | 2.60 | 0.10 | 0.00 |
| 17 | *Metacordyceps* | 0.00 | 0.00 | 1.10 | 0.10 |
| 18 | *Monosporascus* | 0.00 | 21.80 | 0.00 | 0.00 |
| 19 | *Penicillium* | 0.70 | 2.60 | 2.40 | 1.30 |
| 20 | *Phlyctis* | 0.90 | 0.60 | 0.20 | 0.90 |
| 21 | *Poaceascoma* | 0.20 | 7.70 | 0.00 | 0.40 |
| 22 | *Purpureocillium* | 0.10 | 0.00 | 3.10 | 0.10 |
| 23 | *Rhexodenticula* | 0.00 | 0.00 | 1.00 | 0.10 |
| 24 | *Rhizoctonia* | 0.00 | 0.00 | 0.90 | 2.00 |
| 25 | *Robillarda* | 0.00 | 0.00 | 1.00 | 0.20 |
| 26 | *Saitozyma* | 0.00 | 0.00 | 0.90 | 0.00 |
| 27 | *Sarocladium* | 0.00 | 3.10 | 0.00 | 0.00 |
| 28 | *Sistotrema* | 0.00 | 10.60 | 1.70 | 2.50 |
| 29 | *Talaromyces* | 1.20 | 1.60 | 0.90 | 0.50 |
| 30 | *Thelonectria* | 0.00 | 0.10 | 0.80 | 0.00 |
|  | Others | 2.10 | 4.70 | 6.00 | 5.90 |
|  | **Total** | **100.00** | **100.00** | **100.00** | **100.00** |

**Table S12:** Relative percentage abundance of fungal species in the root-nodules of the three *Desmodium* species

| **S/No** | **Species** | **GLD** | **SLD** | **AID** |  |
| --- | --- | --- | --- | --- | --- |
| 1 | *Acrocalymma pterocarpi* | 0.70 | 2.20 | 0.00 |  |
| 2 | *Aspergillus aureoterreus* | 0.00 | 0.00 | 1.20 |  |
| 3 | *Atractiella rhizophila* | 0.00 | 2.10 | 0.70 |  |
| 4 | *Botryosphaeria* spp | 0.00 | 3.10 | 8.00 |  |
| 5 | *Ceratobasidium ramicola* | 0.00 | 2.60 | 0.00 |  |
| 6 | *Cladophialophora* spp | 0.30 | 0.70 | 1.20 |  |
| 7 | *Cladosporium subuliforme* | 1.50 | 0.60 | 0.10 |  |
| 8 | *Clonostachys* spp | 27.30 | 6.10 | 0.00 |  |
| 9 | *Codinaea acaciae* | 1.10 | 3.70 | 0.10 |  |
| 10 | *Cutaneotrichosporon debeurmannianum* | 1.80 | 1.40 | 1.00 |  |
| 11 | *Epicoccum sorghinum* | 1.30 | 0.20 | 0.20 |  |
| 12 | *Fusarium chlamydosporum* | 0.30 | 0.00 | 0.60 |  |
| 13 | *Fusarium sacchari* | 15.40 | 17.20 | 10.90 |  |
| 14 | *Fusarium solani* | 4.50 | 12.60 | 62.50 |  |
| 15 | *Fusarium sporotrichioides* | 0.40 | 2.70 | 0.00 |  |
| 16 | *Fusarium* spp | 12.20 | 2.20 | 1.70 |  |
| 17 | *Knufia* spp | 6.90 | 9.60 | 1.30 |  |
| 18 | *Melanconiella spp* | 0.20 | 2.00 | 0.00 |  |
| 19 | *Monosporascus* spp | 0.00 | 0.00 | 5.70 |  |
| 20 | *Penicillium rubidurum* | 0.00 | 0.00 | 0.80 |  |
| 21 | *Penicillium* spp | 1.80 | 0.20 | 0.10 |  |
| 22 | *Phlyctis speirea* | 0.50 | 2.50 | 0.20 |  |
| 23 | *Poaceascoma* spp | 0.30 | 6.00 | 0.10 |  |
| 24 | *Purpureocillium* spp | 0.00 | 2.80 | 0.00 |  |
| 25 | *Rhizoctonia* spp | 0.00 | 0.80 | 0.30 |  |
| 26 | *Sarocladium kiliense* | 3.00 | 0.00 | 0.00 |  |
| 27 | *Scytalidium* spp | 0.00 | 1.30 | 0.00 |  |
| 28 | *Sistotrema* spp | 3.10 | 8.40 | 0.00 |  |
| 29 | *Metacordyceps chlamydosporia* | 0.20 | 0.00 | 0.40 |  |
| 30 | *Talaromyces spp* | 5.00 | 0.20 | 0.10 |  |
|  | Others | 12.40 | 9.10 | 2.60 |  |
|  | **Total** | **100.00** | **100.00** | **100.00** |  |

Silverleaf desmodium (SLD), *Desmodium* *uncinatum*; Green leaf desmodium (GLD), *Desmodium* *intortum*; and African desmodium (AID), *Desmodium incanum*.

**Table S13:** Relative percentage abundance of fungal species in the root-nodules of the three *Desmodium* species as influenced by sampling location

| **S/No** | **Species** | **Homabay** | **Kisumu** | **Siaya** | **Vihiga** |
| --- | --- | --- | --- | --- | --- |
| 1 | *Acrocalymma pterocarpi* | 0.70 | 0.50 | 0.50 | 0.10 |
| 2 | *Aspergillus aureoterreus* | 0.00 | 4.70 | 0.00 | 0.00 |
| 3 | *Atractiella rhizophila* | 1.20 | 0.00 | 0.20 | 1.70 |
| 4 | *Botryosphaeria* spp | 0.30 | 0.00 | 3.60 | 45.90 |
| 5 | *Ceratobasidium ramicola* | 0.10 | 0.00 | 2.90 | 0.00 |
| 6 | *Cladophialophora spp* | 0.50 | 0.20 | 0.10 | 5.70 |
| 7 | *Cladosporium subuliforme* | 0.20 | 0.50 | 1.40 | 0.10 |
| 8 | *Clonostachys* spp | 0.50 | 6.00 | 20.50 | 9.80 |
| 9 | *Codinaea acaciae* | 1.40 | 0.30 | 0.20 | 1.50 |
| 10 | *Colletotrichum nupharicola* | 0.00 | 0.00 | 1.20 | 0.00 |
| 11 | *Cutaneotrichosporon debeurmannianum* | 0.80 | 1.60 | 1.90 | 1.80 |
| 12 | *Epicoccum sorghinum* | 0.10 | 0.00 | 1.30 | 0.60 |
| 13 | *Fusarium chlamydosporum* | 0.00 | 0.00 | 2.20 | 0.00 |
| 14 | *Fusarium sacchari* | 13.60 | 2.10 | 20.10 | 15.10 |
| 15 | *Fusarium solani* | 71.70 | 9.60 | 5.00 | 2.50 |
| 16 | *Fusarium sporotrichioides* | 0.00 | 0.10 | 3.20 | 0.50 |
| 17 | *Fusarium* spp | 0.20 | 16.50 | 4.20 | 0.10 |
| 18 | *Knufia* spp | 3.20 | 0.80 | 10.60 | 1.60 |
| 19 | *Melanconiella spp* | 0.00 | 2.60 | 0.10 | 0.00 |
| 20 | *Metacordyceps chlamydosporia* | 0.00 | 0.00 | 1.10 | 0.10 |
| 21 | *Monosporascus* spp | 0.00 | 21.80 | 0.00 | 0.00 |
| 22 | *Penicillium rubidurum* | 0.00 | 2.60 | 0.00 | 1.00 |
| 23 | *Penicillium* spp | 0.30 | 0.00 | 1.20 | 0.30 |
| 24 | *Phlyctis speirea* | 0.90 | 0.60 | 0.20 | 0.90 |
| 25 | *Poaceascoma* spp | 0.20 | 7.80 | 0.00 | 0.40 |
| 26 | *Purpureocillium* spp | 0.10 | 0.00 | 3.10 | 0.10 |
| 27 | *Rhizoctonia* spp | 0.00 | 0.00 | 0.90 | 2.00 |
| 28 | *Sarocladium kiliense* | 0.00 | 3.10 | 0.00 | 0.00 |
| 29 | *Sistotrema* spp | 0.00 | 10.60 | 1.70 | 2.50 |
| 30 | *Talaromyces* spp | 1.20 | 1.30 | 0.30 | 0.40 |
|  | Others | 2.70 | 6.60 | 12.30 | 5.40 |
|  | **Total** | **100.00** | **100.00** | **100.00** | **100.00** |

**Table S14**: Pairwise total sum scaling (TSS, relative abundance) log2 linear regression (*P* value) of top five fungal genera in the root-nodules of the three *Desmodium* species

| **Genus** | **AID vs SLD** | **AID vs GLD** | **GLD vs SLD** |
| --- | --- | --- | --- |
| *Fusarium* | 0.896 | 0.965 | 0.885 |
| *Clonostachys* | 0.002***** | 0.003***** | 0.673 |
| *Knufia* | 0.789 | 0.641 | 0.497 |
| *Talaromyces* | 0.939 | 0.806 | 0.164 |
| *Penicillin* | 0.548 | 0.605 | 0.542 |
| *Sistotrema* | 0.152 | 0.700 | 0.931 |
| *Poaceascoma* | 0.469 | 0.869 | 0.452 |
| *Botryosphaeria* | 0.998 | 0.186 | 0.015***** |
| *Monosporascus* | 0.334 | 0.334 | 1 |
| *Aspergillus* | 0.894 | 0.870 | 0.887 |

Silverleaf desmodium (SLD), *Desmodium uncinatum*; Green leaf desmodium (GLD), *Desmodium intortum*; and African desmodium (AID), *Desmodium incanum*; Asterisk represents a significant difference (* *P* < 0.005)

**Table S15**: Pairwise total sum scaling (TSS, relative abundance) log2 linear regression (*P* value) of top five fungal species in the root-nodules of the three *Desmodium* species

| **Species** | **AID vs SLD** | **AID vs GLD** | **GLD vs SLD** |
| --- | --- | --- | --- |
| *Clonostachys* spp | 0.002***** | 0.003***** | 0.919 |
| *Fusarium sacchari* | 0.173 | 0.277 | 0.938 |
| *Fusarium* spp. | 0.807 | 0.982 | 0.865 |
| *Fusarium solani* | 0.342 | 0.648 | 0.156 |
| *Sarocladium kiliense* | N/A | 0.334 | 0.334 |
| *Knufia* spp | 0.789 | 0.641 | 0.497 |
| *Sistotrema* spp | 0.152 | 0.07 | 0.931 |
| *Poaceascoma* spp | 0.176 | 0.621 | 0.452 |
| *Botryosphaeria* spp | 0.998 | 0.186 | 0.155 |
| *Monosporascus* spp | 0.334 | 0.334 | N/A |

Silverleaf desmodium (SLD), *Desmodium uncinatum*; Green leaf desmodium (GLD), *Desmodium intortum*; and African desmodium (AID), *Desmodium incanum*; N/A, negligible; Asterisk represents a significant difference (* *P* < 0.005)

**Table S16**: Pairwise total sum scaling (TSS, relative abundance) log2 linear regression (*P* value) of top five fungal genera based on sampling locations

| **Genus** | **Homabay vs Kisumu** | **Homabay vs Siaya** | **Homabay vs Vihiga** | **Kisumu vs Siaya** | **Kisumu vs Vihiga** | **Siaya vs Vihiga** |
| --- | --- | --- | --- | --- | --- | --- |
| *Fusarium* | 0.654 | 0.735 | 0.505 | 0.931 | 0.734 | 0.614 |
| *Codinaea* | 0.212 | 0.045***** | 0.0001***** | 0.286 | 0.041***** | 0.665 |
| *Atractiella* | 0.043***** | 0.136 | 0.580 | 0.579 | 0.022***** | 0.099 |
| *Talaromyces* | 0.026 | 0.291 | 0.016 | 0.285 | 0.984 | 0.180 |
| *Phlyctis* | 0.851 | 0.965 | 0.202 | 0.886 | 0.361 | 0.176 |
| *Monosporascus,* | 0.356 | 0.098 | 0.292 | N/A | N/A | N/A |
| *Sistotrema* | 0.356 | 0.201 | N/A | 0.978 | 0.292 | 0.156 |
| *Poaceascoma* | 0.186 | 0.430 | 0.196 | 0.179 | 0.962 | 0.156 |
| *Clonostachys* | 0.849 | 0.141 | 0.134 | 0.183 | 0.175 | 0.494 |
| *Knufia* | 0.991 | 0.715 | 0.816 | 0.703 | 0.811 | 0.816 |
| *Botryosphaeria* | 0.699 | 0.797 | 0.292 | 0.838 | 0.292 | 0.362 |
| *Purpureocillium* | 0.799 | 0.517 | 0.932 | 0.824 | 0.630 | 0.313 |
| *Cladophialophora* | 0.219 | 0.249 | 0.865 | 0.841 | 0.305 | 0.333 |

N/A, negligible; Asterisk represents a significant difference (* *P* < 0.005)

**Table S17**: Pairwise total sum scaling (TSS, relative abundance) log2 linear regression (*P* value) of top five fungal species based on sampling location

| **Species** | **Homabay vs Kisumu** | **Homabay vs Siaya** | **Homabay vs Vihiga** | **Kisumu vs Siaya** | **Kisumu vs Vihiga** | **Siaya vs Vihiga** |
| --- | --- | --- | --- | --- | --- | --- |
| *Fusarium solani* | 0.337 | 0.537 | 0.634 | 0.093 | 0.093 | 0.203 |
| *Fusarium sacchari* | 0.127 | 0.304 | 0.552 | 0.466 | 0.127 | 0.633 |
| *Knufia* spp. | 0.991 | 0.715 | 0.814 | 0.703 | 0.811 | 0.816 |
| *Codinaea acaciae* | 0.212 | 0.046 | 0.0001***** | 0.286 | N/A | 0.665 |
| *Atractiella rhizophila* | 0.043 | 0.135 | N/A | 0.579 | 0.022***** | 0.099 |
| *Monosporascus* spp | 0.356 | 0.098 | 0.292 | N/A | N/A | N/A |
| *Fusarium* spp | 0.003***** | 0.022***** | 0.0005***** | 0.330 | 0.113 | 0.113 |
| *Sistotrema* spp | 0.356 | 0.201 | N/A | 0.978 | 0.292 | 0.156 |
| *Poaceascoma* spp | 0.186 | 0.566 | 0.196 | 0.048 | 0.196 | 0.043 |
| *Clonostachys* spp | 0.977 | 0.264 | 0.134 | 0.252 | 0.144 | 0.319 |
| *Botryosphaeria* spp | 0.699 | 0.797 | 0.292 | 0.838 | 0.292 | 0.362 |
| *Cladophialophora* spp | 0.037***** | 0.072 | 0.407 | 0.841 | 0.305 | 0.333 |

N/A, negligible; Asterisk represents a significant difference (* *P* < 0.005)

**Table S18:** Alpha diversity metrics (*P* value) of the root-nodule bacteria ASVs form three *Desmodium* species

| **Treatment comparison** | ***Chao1*** | ***Shannon* index** | ***Observe*** | ***Pielou*** |
| --- | --- | --- | --- | --- |
| AID vs SLD | 0.86 | 0.73 | 0.82 | 0.6 |
| AID vs GLD | 0.36 | 0.31 | 0.33 | 0.31 |
| GLD vs SLD | 0.44 | 0.44 | 0.47 | 0.50 |

Silverleaf desmodium (SLD), *Desmodium* *uncinatum*; Green leaf desmodium (GLD), *Desmodium* *intortum*; and African desmodium (AID), *Desmodium incanum*.

**Table S19:** Alpha diversity metrics (*P* value) of the root-nodule bacterial ASVs from three *Desmodium* species, based on sampling location

| **Sampling location comparison** | ***Chao1*** | ***Shannon* index** | ***Observe*** | ***Pielou*** |
| --- | --- | --- | --- | --- |
| Homabay vs Kisumu | 0.19 | 0.24 | 0.19 | 0.88 |
| Homabay vs Siaya | 0.039* | 0.039* | 0.027* | 0.053 |
| Homabay vs Vihiga | 0.18 | 0.22 | 0.18 | 0.71 |
| Kisumu vs Siaya | 0.35 | 0.35 | 0.35 | 0.29 |
| Kisumu vs Vihiga | 0.80 | 1.00 | 1.00 | 1.00 |
| Siaya vs Vihiga | 0.15 | 0.15 | 0.15 | 0.12 |

Asterisk represents a significant difference (* *P* < 0.005)

**Table S20:** The influence of *Desmodium* species and sampling location on bacterial communities according to PERMANOVA test results.

| **Compartment (bacteria)** | ***df*** | ***R*^2^** | **SS** | ***F* value** | ***P* value** |
| --- | --- | --- | --- | --- | --- |
| *Desmodium* species | 2 | 0.299 | 0.084 | 0.963 | 0.456 |
| Residuals | 21 | 3.265 | 0.916 | - | - |
|  | 23 | 3.564 | 1 | - | - |
| Sampling location | 3 | 1.676 | 0.470 | 5.915 | 0.003* |
| Residuals | 20 | 1.889 | 0.530 | - | - |
|  | 23 | 3.564 | 1 | - | - |

Degrees of freedom (*df*); sum of squares (SS). Asterisk (*) represents a significant difference (*P* < 0.005) based on 999 permutations.

**Table S21:** Alpha diversity (*P* value) metrics of the root-nodule fungal ASVs from three *Desmodium* species

| **Treatment comparison** | ***Chao1*** | ***Shannon* index** | ***Observe*** | ***Pielou*** |
| --- | --- | --- | --- | --- |
| AID vs SLD | 0.15 | 0.61 | 0.56 | 0.75 |
| AID vs GLD | 0.75 | 0.68 | 0.91 | 0.69 |
| GLD vs SLD | 0.31 | 0.24 | 0.24 | 0.28 |

Silverleaf desmodium (SLD), *Desmodium* *uncinatum*; Greenleaf desmodium (GLD), *Desmodium* *intortum*; and African desmodium (AID), *Desmodium incanum*.

**Table S22:** Alpha diversity metrics (*P* value) of the root-nodule fungal ASVs from three *Desmodium* species, based on sampling location

| **Sampling location comparison** | ***Chao1*** | ***Shannon* index** | ***Observe*** | ***Pielou*** |
| --- | --- | --- | --- | --- |
| Homabay vs Kisumu | 0.081 | 0.56 | 0.28 | 1.00 |
| Homabay vs Siaya | 0.330 | 0.85 | 0.47 | 0.64 |
| Homabay vs Vihiga | 1.00 | 0.56 | 1.00 | 0.41 |
| Kisumu vs Siaya | 1.00 | 0.85 | 0.95 | 0.84 |
| Kisumu vs Vihiga | 0.18 | 0.41 | 0.25 | 1.00 |
| Siaya vs Vihiga | 0.36 | 0.53 | 0.49 | 1.00 |

**Table S23:** The influence of *Desmodium* species and sampling location on fungal communities according to PERMANOVA test results.

| **Compartment (fungi)** | ***df*** | ***R*^2^** | **SS** | ***F* value** | ***P* value** |
| --- | --- | --- | --- | --- | --- |
| *Desmodium* species | 2 | 0.908 | 0.107 | 1.263 | 0.091 |
| Residuals | 21 | 7.548 | 0.892 | - | - |
|  | 23 | 8.456 | 1 | - | - |
| Sampling location | 3 | 1.277 | 0.151 | 1.186 | 0.118 |
| Residuals | 20 | 7.179 | 0.849 | - | - |
|  | 23 | 8.456 | 1 | - | - |

Degrees of freedom (*df*); sum of squares (SS)
